# Bridging the gap: returning genetic results to indigenous communities in Latin America

**DOI:** 10.3389/fgene.2023.1304974

**Published:** 2023-11-28

**Authors:** Epifanía Arango-Isaza, María José Aninao, Roberto Campbell, Felipe I. Martínez, Kentaro K. Shimizu, Chiara Barbieri

**Affiliations:** ^1^ Department of Evolutionary Biology and Environmental Studies, University of Zurich, Zurich, Switzerland; ^2^ Center for the Interdisciplinary Study of Language Evolution, University of Zurich, Zurich, Switzerland; ^3^ Pontifical Catholic University of Peru, Lima, Peru; ^4^ Escuela de Antropología, Facultad de Ciencias Sociales, Pontificia Universidad Católica de Chile, Santiago, Chile; ^5^ Center for Intercultural and Indigenous Research, Santiago, Chile; ^6^ Department of Life and Environmental Sciences, University of Cagliari, Cagliari, Italy

**Keywords:** Latin America, indigenous communities, genomics, Global South, ethics, Chile

## Abstract

In response to inequality in access to genomics research, efforts are underway to include underrepresented minorities, but explicit (and enforcing) guidelines are mostly targeted toward the Global North. In this work, we elaborate on the need to return scientific results to indigenous communities, reporting the actions we have taken in a recent genomic study with Mapuche communities in Chile. Our approach acknowledged the social dynamics perpetuating colonial hierarchies. We framed genetic results to empower indigenous knowledge and communities’ history and identities. A fundamental step in our strategy has been sharing the results with the communities before publishing the scientific paper, which allowed us to incorporate community perspectives. We faced the challenge of translating genetic concepts like admixture, emphasizing the distinction between identity and biology. To reach a broad and diverse audience, we disseminated the study results to single community members, cultural representatives, and high schools, highlighting the importance of the history of the region before the European contact. To facilitate results dissemination, we prepared didactic material and a report in Spanish written in non-specialized language, targeting a wider Latin American readership. This work illustrates the benefits of discussing scientific findings with indigenous communities, demonstrating that a collaborative and culturally sensitive approach fosters knowledge sharing and community empowerment and challenges power dynamics in genetic research. Bridging the gap between academia and indigenous communities promotes equity and inclusion in scientific endeavors.

## 1 Introduction

Genomic research has revolutionized our understanding of human history, ancestry, and the genetic basis of diseases. However, it is essential to acknowledge that this field has not been exempt from the pervasive issue of inequality. The past and present of genomic research urgently need to address disparities and foster a more inclusive approach (Popejoy and Fullerton, 2016; Atkinson et al., 2022; Fatumo et al., 2022; Villanea and Witt, 2022). After decades of analyzing cohorts of (almost exclusively) European descent, geneticists recognized the importance of increasing diversity and representation in genomic studies, particularly among minorities and indigenous communities (Oh et al., 2015; Peterson et al., 2019; Dajani et al., 2022). Initiatives are underway to expand the sampling pool and include populations historically underrepresented in research efforts. However, these initiatives often face unique challenges and complexities, starting from the position of indigenous communities who harbor mistrust towards the academic world due to past practices of exploitation that are still vivid in their collective memory (Garrison et al., 2019; Mc Cartney et al., 2022). While ethical guidelines regarding genomic research have gained considerable attention in high-income countries like the United States, New Zealand, and Australia (Claw et al., 2018; Collier-Robinson et al., 2019; Garrison et al., 2019; 2020; Caron et al., 2020; Hudson et al., 2020; Tsosie et al., 2020), a striking lack of literature addresses the specific context and challenges researchers encounter in the Global South (Zavala, 2013; Australian Institute of Aboriginal and Torres Strait Islander Studies, 2020; Silva et al., 2022; Ávila-Arcos et al., 2022). In a recently published study on the genetic history of Mapuche groups in Chile (Arango-Isaza et al., 2023), we incorporated several elements to foster inclusivity, transparency, and best ethical practices when working with indigenous groups. Reporting on particular challenges and procedures from specific research studies provides solutions to implement and adapt to the circumstances of other similar research studies (Rodriguez et al., 2022). With the present paper, we want to describe our research strategies, reflecting on actions that positively impacted the communities, the genetic study, and aspects that could be improved.

When researching within or about countries located in the Global South, scientists need to be aware of the local context to avoid past mistakes. These challenges are not exclusive to Latin America but are the focal point of this particular publication. In Latin America, indigenous communities and other marginalized groups, such as Afro-descendants, confront significant challenges associated with land conflicts and structural racism. Power dynamics of political, economic, and cultural pressure reinforce existing inequality structures inherited by colonialism in what is referred to as Neocolonialism (Maldonado-Torres, 2016). Estimates derived from a census in 2015 indicate that self-identified indigenous people constituted approximately 8%–10% of the population in the Caribbean and Latin America by 2018, totaling around 60 million people, a consistently growing number (CEPAL, 2020; Oliva Martínez, 2022; Flores et al., 2023). These indigenous groups endure ongoing social, economic, and political marginalization rooted in historical power imbalances and systemic inequalities (Cusicanqui, 2012). The scientific discourse has inadvertently contributed to this exoticization of indigenous groups by perpetuating the idea of a “pristine” indigenous identity (indigenous purism) and linking it to specific genetic profiles (an aspect of essentialism or genetic determinism). This concept erroneously connects indigenous identity or tribal affiliation to DNA and implies that one’s genome dictates one’s characteristics as an individual or collective (Nordgren and Juengst, 2009; Blanchard et al., 2019). Indigenous communities are diverse and dynamic, comprising various cultures, languages, and traditions (Ryan-Davis and Scalice, 2022). Like the broader society, indigenous communities have changed over time, challenging the misconception of their static nature (Makaran and Gaussens, 2020). Cultural and political discourse has often portrayed indigenous groups as static, rural communities serving as custodians of the land and traditional knowledge. However, this narrow depiction fails to capture the true diversity of indigenous identities today and, notably, overlooks a significant demographic: descendants of indigenous people who have emigrated into urban centers. By reinforcing the stereotype of distant “others”, the prevailing narrative excludes the majority of the indigenous population and decreases their capacity for substantial influence and the potential to shape the trajectory of the state (Cusicanqui, 2012). While there is no precise data on the indigenous population in urban areas, it is estimated that around 50% of the indigenous population lived in Latin American cities by 2010 (Del Popolo, 2018). This urban migration often results in individuals of indigenous descent occupying the lowest social strata and engaging in informal labor (Oliva Martínez, 2022).

The trajectory towards ethical and considerate conduct in genomic research hinges on the scientific community’s approach. Scientists are called to embrace cultural humility, meticulously eschewing broad generalizations and detrimental stereotypes that perpetuate damaging narratives or undermine the autonomous agency of indigenous peoples and their descendants, irrespective of their residential context (Pappas, 2020). An earnest recognition of the historical and persistent marginalization experienced by these communities needs to proactively elevate the ethical standards governing research conducted within these regions (Zavala, 2013; Schwartz-Marin and Fiske, 2022; Silva et al., 2022).

Conversely, genomic initiatives in Latin America contrast these forms of indigenous purism with a narrative referred to as the “mestizo rhetoric” (Séguin et al., 2008; Wade et al., 2014b; Kent et al., 2015). This rhetoric, aimed at constructing a unified political identity, has been explored by numerous scholars who seek to group diverse populations under the label of “mestizo”. However, this endeavor marginalizes other minority groups (Telles and Bailey, 2013; Wade et al., 2015; Rodríguez Mega, 2021; Silva Gallardo and González Zarzar, 2023). Paradoxically, this homogenizing effort has failed to eliminate disparities, even among those self-identified as mestizos. Individuals with mixed indigenous and non-indigenous heritage also bear the consequences of internalized colonialism, illustrating that the legacy of oppression extends beyond just minority communities but also those with diverse backgrounds (Fanon, 1961). The mestizo rhetoric idealizes a vision that fails to address socioeconomic disparities and conceals cultural and political privileges deriving from early colonialism (Cusicanqui, 2012). In several Latin American countries, there is a positive correlation between the percentage of European ancestry and socioeconomic status, in contrast to the Native American/Amerindian ancestry (Avena et al., 2012; Campbell et al., 2012; Bonilla et al., 2015; Barozet et al., 2021). This is a compelling illustration of how colonial historical legacies influence contemporary societal structures, shedding light on the intricate relationships between genetics, identity, and privilege in these regions. Addressing these multifaceted challenges requires comprehensive efforts to dismantle oppressive systems, promote inclusivity, and ensure equal rights and opportunities for all individuals, regardless of their background or ethnicity (González Casanova, 2006). It is worth noting that many Latin American countries have not had meaningful conversations regarding their stance on genetic research (Wade et al., 2015). The discussion surrounding genomic research in the Latin American context, encompassing the mestizo rhetoric and indigenous purism, must be examined alongside the pressing issues faced by indigenous communities, Afro-descendants, and mestizos. By considering the historical and current challenges and prioritizing ethical considerations and inclusivity, researchers can work towards meaningful and equitable engagement with these communities in genetic research endeavors (Silva et al., 2022).

A key factor in opening scientific research to indigenous representation and abandoning neocolonialist practices is transparency in research protocols and community participation. The involvement of participants and the community in a broader sense is essential to guarantee respect for cultural settings, benefit for the participants, and enrichment of the scientific study. The need for the involvement of participants has been highlighted by indigenous representatives and indigenous geneticists and recognized by scientists from Western institutions (Claw et al., 2018; Garrison et al., 2019). This can be done from the beginning of the study design to the discussion of the scientific results. Returning the genetic result of a study has been largely neglected in the past. Until today, it is often conducted out of the spontaneous initiative of the geneticists responsible for the project and is not enforced by the institute’s best scientific practice. In recent years, there have been examples of studies that included the return and discussion of the results prior to the drafting of the scientific publication as a requirement in the research agreement (Rodriguez et al., 2022).

This paper illustrates and contextualizes our experience conducting a project on the genetic history of Mapuche populations in Chile. The majority of the researchers involved in the primary genetic study and the main authors of the current paper are located in the Global North; this factor is recognized as a limitation and potential source of bias, as our positionality and cultural background can influence our perspectives and interpretations. Our specific goal is to ascertain the qualitative impact of our collaborative and participatory efforts, illustrating the potential for constructive engagement with local communities. By recognizing the importance of community engagement and meaningful participation, we aimed to make research findings accessible, culturally sensitive, and beneficial to the community. Moreover, we focus on how these collaborative endeavors have impacted the scientific knowledge generated by our research. Finally, we provide a list of actions and recommendations in line with developing guidelines for genetic research in Latin America. Through sharing our experiences and lessons learned, we hope to foster dialogue, collaboration, and critical reflection within the scientific community, ultimately contributing to more inclusive and respectful research practice.

## 2 Challenges and threats to indigenous identity and genetic legislation in Chile

The Chilean population, estimated at 19.5 million, is predominantly of mixed genetic origin, like the population of most Latin American countries. Genetic analysis methods recognize two main ancestral components: the Amerindian, usually referred to as “Native American”, and the European (Fuentes et al., 2014; Barozet et al., 2021). Current legislation recognizes 10 Indigenous groups in Chile (with an 11th group in the process of being recognized), of which the Mapuche constitute the largest group (Sandoval et al., 2023). However, despite their significant presence, indigenous communities, including the Mapuche, face socio-economic challenges such as higher poverty levels, indigence, and lower life expectancy than non-indigenous populations (Agostini et al., 2010; Sandoval et al., 2022). During the 20th century, the Mapuche population was involved in an intense internal migration from the rural areas to the cities (Antileo, 2014). Of the Mapuche population, 62.4% live in the cities, of which around 30% are in the capital city of Santiago (Reynoso and Sánchez, 2020). While the majority of Mapuche people and their descendants reside in Santiago, their original territory, known as Wallmapu, encompassed central and southern Chile, spanning from the Atlantic to the Pacific coast in Argentina (Zúñiga, 2006; Vitar, 2010). Since the 19th century, with the occupation of Araucanía, this territory has been threatened by the Chilean state with a consistent pattern of land appropriation that closely mirrors settler colonialism’s historical practices (Aylwin Azócar et al., 2003; Wolfe, 2006; Nahuelpan Moreno and Antimil Caniupán, 2019; Correa Cabrera, 2021; Martínez Navarrete, 2021). Over the past 3 decades, Mapuche land has been reduced by approximately 510,000 ha due to the privatization of indigenous territories since 1973 (Foerster, 2002; Klubock, 2014; Gómez-Barris, 2017; Nahuelpan Moreno and Antimil Caniupán, 2019). Many of these areas have been converted into monocultures, particularly in the Biobío region, with non-native plants like eucalyptus and radiata pine replacing the local forests (Lara et al., 2012; Martínez Navarrete, 2021).

The 1980 constitution in Chile does not recognize the existence of indigenous people. Currently, the sole legal framework offering limited protection is the Indigenous Law from 1993 (Law 19253). This legislation paved the way for the establishment of CONADI (Corporación Nacional de Desarrollo de los Pueblos Indígenas), responsible for implementing policies and programs to promote the development and wellbeing of indigenous peoples in Chile. Between 2001 and 2003, the “Comisión Verdad Histórica y Nuevo Trato con los Pueblos Indígenas “(Historical Truth and New Deal with Indigenous Peoples Commission) aimed to address historical issues and demands of indigenous peoples. This commission recognized the diversity of indigenous cultures and identified past injustices. Its recommendations aimed for a new relationship between the Chilean state and indigenous people, including land restitution, cultural recognition, and consultation on matters affecting indigenous rights (Aylwin Azócar et al., 2003). In 2004, the Indigenous Law was completed by introducing the “New Treatment politics”, an attempt to establish a new relationship with the indigenous communities. Although Chile has adhered to the UN Declaration on the Rights of Indigenous Peoples and the International Labour Organization 169 Convention since 2009, evidence suggests inadequate compliance with these international laws (Lucic and Guzmán, 2018). At the moment, none of these laws (or the creation of CONADI) have managed to solve the territorial conflict in Chile. The land claims remain one of the priorities for the indigenous political movement in Chile (Oliva Martínez, 2022).

In the field of genetic research, studies conducted in Chile have contributed to understanding the genetic history and population dynamics of various groups, including the Mapuche (Acuña P. et al., 2000; De Saint Pierre et al., 2012; Fuentes et al., 2014; De la Fuente et al., 2015; Eyheramendy et al., 2015; De la Fuente et al., 2018; Verdugo et al., 2020; Barozet et al., 2021; Arango-Isaza et al., 2023). The existing Law 20.120, in force since September 2006, focuses on scientific research in human beings and their genome while prohibiting human cloning. This law requires authorization from the relevant authorities and a favorable report from an accredited ethical scientific committee before conducting the study. It also established the National Bioethics Commission and emphasized the importance of informed consent (Vargas Catalán and Millán Klüsse, 2016). Nevertheless, the current legal framework does not explicitly and specifically safeguard indigenous communities’ genetic privacy and rights (Silva et al., 2022).

## 3 Methodology

### 3.1 Data collection and availability

The genetic study of the Mapuche ancestry in Southern Chile was framed with interdisciplinary perspectives from anthropology, archaeology, and linguistics. For the study, genetic data was retrieved with a new sample collection from individuals who either self-identified as having Mapuche ancestry or resided in regions with a historically attested Mapuche presence. The research project and sample collection were approved by the Unidad de Ética y Seguridad de Investigación of the Pontificia Universidad Católica de Chile’s Institutional Review Board (project #171009001, decree #1520863561038). All project stages adhered to the principles of the Declaration of Helsinki Association (World Medical Association, 2013).

Sampling was conducted in early 2019 by two project members, a geneticist and a linguist of Mapuche descent. The sampling trip took place in the Araucanía region and on the island of Chiloé. Before and/or during the sampling process, consultation took place with local authorities such as municipalities, cultural centers, and *lonkos* (traditional leaders of Mapuche communities). This collaborative approach ensured respectful engagement and adherence to local protocols. The precise sampling locations were not disclosed to protect the privacy of participants. Extensive time (minimum one hour, usually three to four hours) was dedicated to explain the project to a potential participant and building reciprocal trust. The conversations were held in Spanish, with the two project members proposing a colloquial atmosphere. The potential participants were not pressured into participating in the project. After receiving extensive explanations of the project’s aims and conditions, the 67 participants who voluntarily agreed to donate a sample could sign an informed consent and receive a copy for them to keep. Samples consisted of 2 mL of saliva collected in Oragene tubes from DNAgenotek and stored with an anonymous code. A second random anonymization step was carried out before processing the samples in the laboratory so neither the participants nor the geneticist who collected the sample could link the sample code to a specific person. This extra step was performed in case they could remember the sample code associated with a participant.

The data generated (SNP chip genotypes, with ∼600,000 SNPs typed per individual) were deposited in the European Genome-phenome Archive (EGA; https://ega-archive.org/) with the accession number EGA: EGAS00001007200. The data is not publicly available but under access control, restricted to research purposes related to human genetic history. Access to the data is granted by a Data Access Committee composed of principal authors from the University of Zurich and the Universidad Católica de Chile, subject to the conditions outlined in the Data Access Agreement Form, which is available upon request.

### 3.2 Returning the genetic results

After finalizing the data analysis, we returned the research results to the participants and the broader local population. We took several steps to disseminate the results effectively, making the findings accessible to both the participants and the general public in an inclusive manner and distancing from the exclusiveness of the scientific publication in English, written in academic jargon.

To begin with, we organized a return expedition trip in early 2022 with three project members: the Ph.D. student responsible for the genetic analysis, the genetic principal investigator who conceptualized the study, and the main local researcher with extensive field experience in the area and a vital local network, being the last two also the ones who collected the data in 2019. During this expedition, our main objective was to ensure effective communication. We recognized the importance of Mapudungun, the local Mapuche language, which is the subject of revitalization practices with dedicated courses at school, but we realized that Spanish is universally spoken in the region, so we decided to use Spanish to translate the scientific results from English, the language of the scientific publication. The results were organized in a slide presentation, a common medium for scientific dissemination. Recognizing that not everyone had access to screens or video projectors, and to add material value to our dissemination aids, we turned the slide display into a printed version in A3 format that could be conveniently displayed and understood without additional technology. The slides are available as Supplementary Material S1. 16 meetings were organized to present the results; in each session, a minimum of one person and a maximum of ∼15 people were present. In the meetings, we worked to include a very diverse audience: for example, active members of the communities engaged in keeping the culture alive, teachers, people with doctoral degrees as well as people with no state education, women and men, and children of various ages. Moreover, we actively engaged with local schools in each study area. We conducted three lectures tailored for students and teachers, one in each community area, ensuring that our findings reached the younger generation and educational professionals who play a crucial role in knowledge dissemination. For the presentations in high schools, we modified and expanded the material to provide a more comprehensive insight into genetics and stress the implications of our results for rooting the region’s history within the community.

The COVID-19 pandemic significantly impacted our plans. Our ability to travel and engage with people and communities was restricted, and our actions were contingent on ever-changing COVID-19 reports. Not only did we adhere to the quarantine periods mandated by the government upon arrival, but we also proactively implemented additional self-determined safety measures to safeguard the wellbeing of the participants, like wearing FP2 masks and carrying rapid diagnostic testing every 3 days.

Our collaboration and continuous dialogue with local stakeholders proved invaluable throughout the process. Their insights and perspectives were instrumental in framing our research questions, presenting our results in culturally appropriate ways, and maintaining sensitivity to the cultural and social context of the study areas. Notably, the return expedition was conducted before writing the genomic manuscript, enabling us to incorporate participants’ suggestions and feedback into the final publication. This approach ensured that their voices and contributions were respectfully acknowledged. As mentioned above, this particular step is only sometimes applied to genetic studies, with relevant exceptions (Rodriguez et al., 2022).

After the publication of the genomic manuscript (Arango-Isaza et al., 2023), we prepared a comprehensive report in Spanish that was distributed to both the participants and can be diffused online to the general Latin American public (Supplementary Material S2). This report serves as a platform for scientific dissemination and provides a broad understanding of molecular biology, population genetics, and the key genetic results.

## 4 Engaging mapuche indigenous communities: challenges and considerations in returning genetic scientific results in the Latin American context

### 4.1 Communicating scientific results: how to make scientific language accessible to a broad audience

**FIGURE 1:**
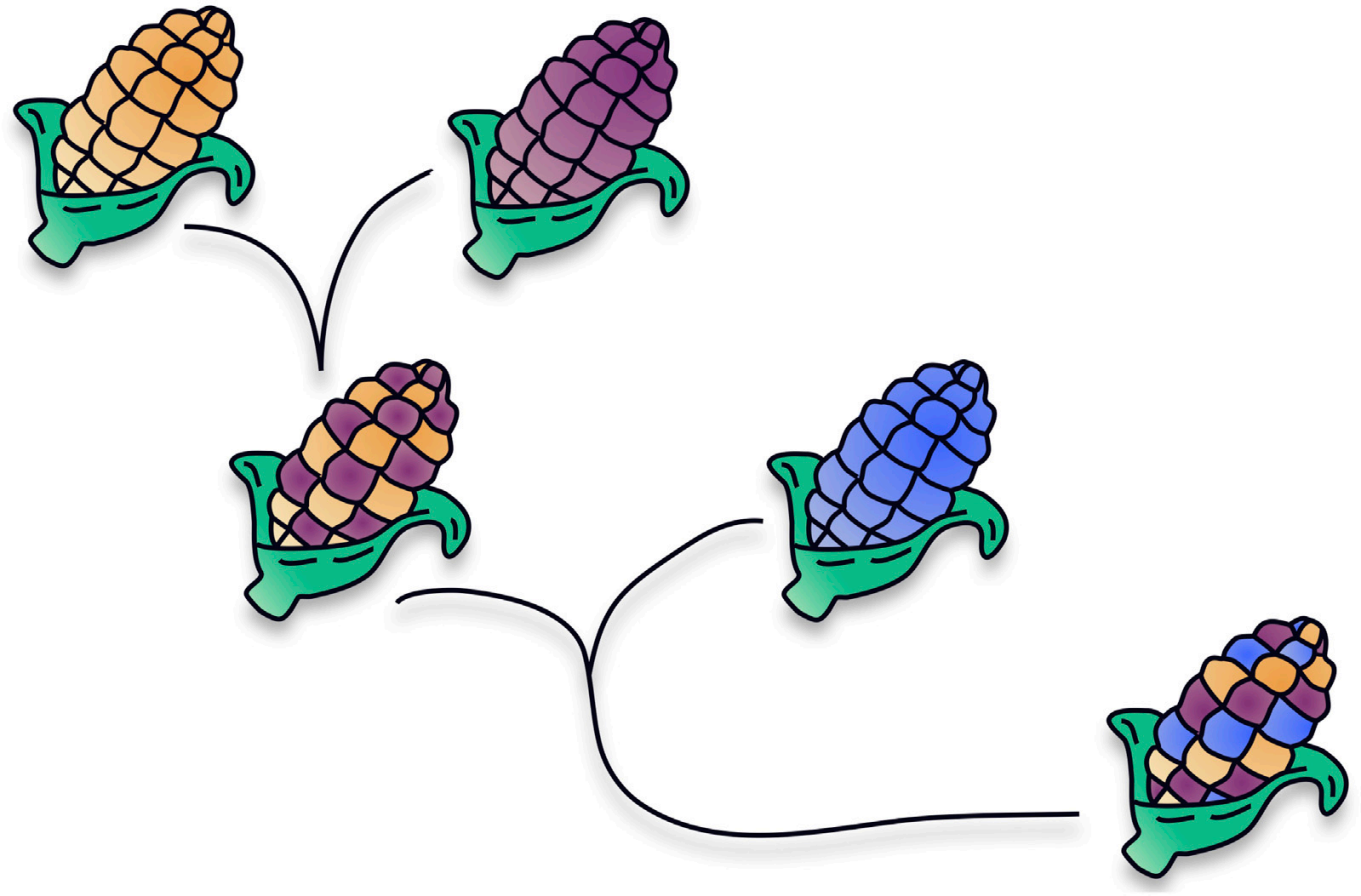
Colored corn analogy for explaining heritability from Supplementary Material S1. An analogy using colored corn was employed during our engagement with Mapuche Indigenous communities to explain heritability. Each colored kernel represented observable traits influenced by genetics, demonstrating how traits are passed down from parents to offspring.

It is a fundamental right for both the participants and the broader region audience to have access to the study results (Hudson et al., 2020). As the participants in our project and the general audience exhibit diverse knowledge on biology, we adapted the language use to communicate the study findings. Our communication strategies aimed at maintain clarity without compromising the quality of the message (Hintz and Dean, 2020). We insisted on analogies, a well-established science teaching strategy to bridge the gap between complex scientific concepts and everyday language (Aubusson et al., 2006). For example, we used the analogy of the DNA molecule as a book that stores information or the use of colored corn to explain genetic diversity and heritability (Figure 1). These analogies were relatable and accessible entry points for explaining key biological concepts to the participants.

**FIGURE 2:**
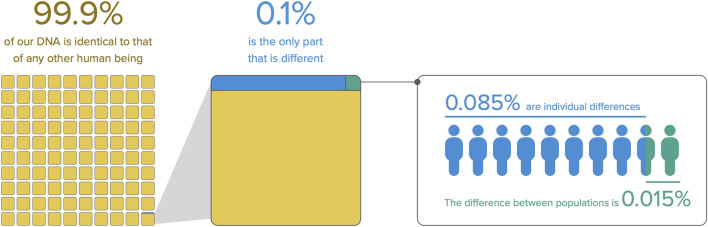
Distribution of genetic diversity among humans. This figure shows how genetic diversity is distributed among human populations. Part of Supplementary Material S2 (Lewontin, 1972; Lander et al., 2001; Hunley et al., 2016; Novembre, 2022).

During fieldwork, we encountered widespread misconceptions about genetic ancestry, and a tendency towards genetic essentialism (Graves Jr, 2002; Donovan, 2017; Donovan et al., 2019). Even among geneticists, there is a misuse of certain terms that inadvertently reinforce social and genetic profiles, suggesting a genetic basis for racial categories - even if no biological support exists for those (Wade et al., 2015; Silva Gallardo and González Zarzar, 2023). The terms “race”, ethnicity, and genetic ancestry are often interpreted as overlapping concepts in scientific and popular literature (Yudell et al., 2016). For example, our interlocutors wondered if their genetic ancestry determines physical traits and unique cultural practices and traditions, revealing a subtle yet prevalent tendency towards genetic essentialism. We explicitly addressed this misconception by explaining that our identity is not rooted in our genetic ancestry (see further notes in Section 4.4). We also showed that the genetic diversity among any human individuals is limited, and that genetic diversity is primarily found among individuals rather than between populations, as illustrated in Figure 2, from the scientific report (Supplementary Material S2) (Lewontin, 1972; Hunley et al., 2016). Additionally, we explained the process of DNA sequencing in simple terms and presented the scale of human migrations in the world and South America, highlighting the power of genetics in understanding human history (Supplementary Material S2).

Creating an environment that fostered participant engagement was crucial. We encouraged questions during and after our presentations, recognizing that open dialogue and oral communication are central to Mapuche communities (Bañales-Seguel et al., 2020). We also acknowledged that certain genetic findings might challenge traditional or religious community beliefs or perspectives (Ávila-Arcos et al., 2022). To address this sensitivity, we included a dedicated slide (Supplementary Material S1. Slide 6) and paragraph in the report (Supplementary Material S2) emphasizing that scientific knowledge is just one facet of overall knowledge and should not be seen as dominant or absolute truth.

“*Knowledge is vast and includes other ways of understanding the world, such as experience, tradition, legends and myths, among others. These types of knowledge are not mutually exclusive but can complement each other to obtain a broader vision of reality. Science is simply one of the ways of acquiring knowledge about the world.”*

We underscored the importance of fostering dialogue between knowledge systems (Escobar, 2016; Bañales-Seguel et al., 2020). During our discussions and conversations with community members, we posed questions such as “What are your thoughts on this finding?” and “Does it align with your previous notions?” or even “How do you feel about these results?” These questions encouraged open dialogue and created an inclusive atmosphere for diverse viewpoints. As we delivered and discussed scientific knowledge, participants could make informed interpretations and engage in meaningful discussions on the research findings. Through these efforts, we aimed to foster scientific literacy and promote a more informed participation in genetic research.

### 4.2 The relevance of local collaborators: ensuring cultural translation in genetic research

The research project was enriched through the involvement of local collaborators and stakeholders, most of whom identified as Mapuche, while some did not (*winka* is the Mapudungun word for non-Mapuche people). As highlighted by other scholars, the local population’s participation benefits both academia and the community (Copete et al., 2023). These connections were established during our trip and through the extensive network of the main local researcher. Their contributions were instrumental across multiple facets of the project, from study design and data collection to the critical aspects of the results dissemination and manuscript preparation. Their involvement enabled us to navigate the intricacies of cultural translation, which extended beyond language proficiency alone. Importantly, the presence of a Mapuche researcher did not exempt the rest of the team from dedicating effort to acquiring cultural competence. By listening to the concerns and perspectives of different community members during the two field trips, the team adapted terminology, phrasing, research angles, and general sensitivity to the local history and culture, cultural norms, non-spoken communication, and political dynamics to ensure effective cross-cultural communication and engagement (Hudson et al., 2020). In the field, researchers can face the initial challenge of establishing connections with participants before introducing the project’s aims and scope. Local collaborators were vital as cultural intermediaries, providing valuable insights into community dynamics, customs, and traditions that might elude outsiders. Their assistance facilitated the exchange of knowledge, ideas, and concerns between researchers and the community members, ensuring that dialogue was respectful, reciprocal, and culturally sensitive. This understanding was crucial in ensuring that the dialogue was conducted respecting the local context and contributing positively to the community’s wellbeing.

It is essential to acknowledge that our research unfolded within a challenging political context. Mapuche communities have been living in a conflictual dynamic with the state in relationship to land ownership and recognition of indigenous culture and rights (Aylwin Azócar et al., 2003). Given the sensitive nature of our work, some local researchers and stakeholders, although deeply committed to our research goal, declined to participate as DNA donors or preferred not to take the role of official coauthors. Instead, they actively engaged in discussions, providing invaluable insights and logistic support while maintaining a degree of anonymity. In a couple of circumstances, during sample collection, community members displayed hostility towards the presence of *winkas* performing genetic studies with Mapuche communities, reporting on known cases of scientific misconduct with the handling of sensitive genetic data from indigenous groups that occurred in the late 90s and early 00s (see Malhi, 2009), or negative experiences of the type “other academics came to study us, but never came back”. In these circumstances, we respected the individual perspectives and left the region or the household without collecting any data. Despite these complexities, the people involved in the step of returning the results overall expressed satisfaction with our collaborative approach, as reflected by the positive feedback obtained during all our 16 meetings. This trust was not solely due to the presence of a Mapuche researcher but also because of our team’s commitment to earning their confidence. We valued the communities’ trust and recognized that it was essential in our research. This collaborative approach allowed us to achieve our research goals effectively.

### 4.3 Engaging with schools: a focus on pre-European-contact history

The history curriculum in Latin America has predominantly focused on the last five centuries from the Spanish occupation, often neglecting the continent’s rich and diverse prehispanic heritage. There is an awareness of the need for a representation of the continent’s history that gives justice to its cultural roots before the arrival of the Europeans (Cisternas Irarrázabal, 2018). To address this historical imbalance, we emphasized how the Americas were populated long before the Spanish colonization. Throughout our field presentations, we took chances to highlight the cultural and ethnic diversity of the continent’s populations before Europeans arrived. It is important to consider how educational institutions during the 20th century served as “civilizing” entities under the pressure of Chilean elites, who promoted the narrative of Mapuche people as “second-class” citizens (Merino et al., 2007; Nahuelpan Moreno and Antimil Caniupán, 2019). Additionally, assimilationist educational policies have dominated national and international contexts. Indigenous children worldwide experienced the active eradication of their cultural identity through humiliation, psychological violence, and even physical abuse, which were part of what has been termed “cultural genocide” (Commission de Vérité et Réconciliation du Canada, 2015).

From the beginning of the colonial period, after the independence of Chile and the subsequent occupation of the territories south of the Biobío river, which were managed by Mapuche communities, Mapuche children in schools and institutions were prohibited from speaking their language and subjected to various forms of physical and psychological abuse (Reynoso and Sánchez, 2020; Dillon et al., 2022). The curriculum effectively erased indigenous history, denying Mapuche students the opportunity to connect with the heritage of their community (Manning and Harrison, 2018). This erasure created a tense dynamic between teachers and indigenous students, stemming from the educators’ lack of knowledge about local and cultural history and their limited understanding of indigenous education. These factors restricted education to a nationwide monocultural and homogeneous curriculum, perpetuating prejudices against the indigenous population (Arias-Ortega et al., 2023). Even today, the educational system has not met the local populations’ educational, social, cultural, and linguistic needs (Milne and Wotherspoon, 2023). Schools perpetuated symbolic and cultural domination over the indigenous population, removing students from their social, cultural, and spiritual foundations (Arias-Ortega and Quintriqueo, 2021). During our fieldwork experience, we had conversations with several elderly Mapuche people who explained how they consciously chose not to teach Mapudungun to their children, to protect them from discrimination. These interactions show how deeply rooted prejudice and cultural marginalization have affected Mapuche communities for generations.

In more recent times, an increasing awareness of the value of cultural and linguistic diversity, coupled with rising attention to human and indigenous rights, has enhanced the way for the implementation of intercultural and bilingual education programs in Chile such as the “Programa Orígenes” and the program for Intercultural Bilingual Education (Figueroa Burdiles et al., 2018; Aguayo et al., 2022). These initiatives have primarily been applied in rural areas and, while they still require refinement, have contributed to a stronger sense of indigenous identity among the students (Cisternas Irarrázabal, 2018).

Our presentations received significant appreciation from the teaching staff at the schools we visited. History teachers, in particular, positively received our research angle on genetic human history (and prehistory) as it provided a more comprehensive understanding of the region’s historical roots. Particularly noteworthy was the positive response from “traditional educators,” who specialize in teaching basic Mapudungun language and Mapuche indigenous traditions. Their expertise and deep connection to indigenous cultures made them particularly receptive to including prehispanic history in the curriculum. Finally, the classes have also been positively received by students becoming more aware of indigenous traditions, either as self-identifying as Mapuche, or recognizing having Mapuche ancestry, or with students who live in contact with Mapuche peers.

By acknowledging the prehispanic history and cultural diversity in the Americas, we encouraged a more inclusive and accurate narrative encompassing indigenous communities’ rich heritage, in line with the claims of diverse indigenous movements (Oliva Martínez, 2022). This approach broadened students’ perspectives, allowing them to develop a more comprehensive understanding of the continent’s history and cultural dynamics.

### 4.4 Addressing misconceptions and sensitive topics in genetics

During our fieldwork with indigenous communities, we encountered several complex and delicate topics that required careful consideration and preparation by our team. We prepared in particular for two aspects of the genetic analysis that traditionally meet sensitive reactions: the concept of Native American and European genetic admixture, and the concept of indigenous identity and genetic determinism. Despite our primary focus being on prehispanic history, our genetic analysis included an overview of the degree and timing of European admixture in the region and the continent. This type of analysis is common practice in a population genetics study and serves to contextualize the rest of the analysis, which focuses on Native American ancestry and prehispanic history. The genetic concept of European admixture is not exempt from the power dynamics and exploitation that brought European lineages into the region. First of all, it was essential to navigate the political connotations associated with terms like “genetic admixture” or “mestizaje”, not only confronting the scientific jargon to the understanding of the layperson but also confronting the use of these terms in English and their equivalents in Spanish. Given the significance of word choice, we used the term “genetic flow” instead, as it carried a less loaded meaning and conveyed our understanding without evoking negative or positive connotations. For instance, we used sentences like “The different populations exhibit a wide degree of European genetic flow.” In our on-site presentations (Supplementary Material S1) and in the final report (Supplementary Material S2), we explained that this observation had no positive or negative connotation. Instead, it simply reflected a part of the historical dynamics that Mapuche communities encountered.

“*Furthermore, it is important to emphasize that gene flow between the settlers and the Mapuche populations should not be interpreted as a measure of superiority or inferiority. Rather, it reflects the complex interactions and cultural mixtures that occurred during this historical period. These genetic interactions are a testimony to the resilience and adaptability of the Mapuche people and their culture, despite colonial influence.”*

Genetic estimates of indigenous ancestry have been associated with questions of identity, even in legal terms, as some indigenous representatives require any person who would like to join a community to apply with a DNA ancestry test to prove their indigenous ancestry (TallBear, 2013; Blanchard et al., 2019). The very delicate question “How indigenous am I?” is not uncommon and bears traces of a genetic deterministic approach that connects cultural identities to specific biological markers. To address this sensitively, we implemented two strategies. Firstly, we provided population-based results rather than individualized ones, ensuring that the focus remained on the broader context rather than individual identity. Secondly, we proactively explored the space between biology and identity with our interlocutors to reflect together on the limitations of such an approach and the nuanced relationship between these concepts (Supplementary Material S2).

*“Genetic ancestry is not equivalent to identity. Identity is constructed through variable elements such as culture, sense of belonging and other factors, and is not determined by biology, or at least not solely.”*

We suggest that geneticists can and should speak up to avoid reducing indigenous identity to mere genetic factors (Silva Gallardo and González Zarzar, 2023). Research has shown how identities can be fluid and even intersectional (Puar, 2012). Identity should not be tied to biological or phenotypical factors but to sociological and cultural elements and the subjective dimension of the self-identification (Oliva Martínez, 2022). Also, we commented on the widespread notion that indigenous people are “less advanced and not evolved”. Our historical, cultural, and biological approach provided tools to reframe colonialist practices in this sense. We stressed that such notions of progress come from power dynamics deeply rooted in the scientific discourse of the Western world and have been confronted in Anthropology since the early 20th century.

*“Another important point is that claiming that one population is more advanced or evolved than another contradicts the principles of biology and is incorrect. Each human population has evolved or developed in a unique way in response to the environment and historical circumstances, which has given rise to today's cultural diversity. No linear scale of progress or superiority can be established among human populations, as all have their own valuable knowledge, traditions, and ways of life.”*

In approaching these delicate topics, we intended to foster respectful and meaningful dialogue while being mindful of the complexities and sensitivities involved, without avoiding problematic topics. We carefully selected our terminology and focused on broader population dynamics to create a space for understanding and appreciation without diminishing community members’ individual experiences and self-identifications.

### 4.5 Moving away from neocolonial science: mitigating biocolonialism and scientific extractivism

We are aware of the limitations inherent in our study and acknowledge the influence of our cultural backgrounds on the research process. To mitigate neocolonial practices, we adopted a collaborative approach involving local scientists from diverse disciplines, ensuring inclusivity and cultural sensitivity throughout the research process. Community engagement and reciprocity played a central role, focusing on sharing the knowledge we generated with the involved communities, thereby respecting participants’ ownership (Hintz and Dean, 2020). We carefully explained our informed consent document, emphasizing clear explanations of the research purpose, potential benefits, risks, and handling of genetic data. These practices have been long enforced in basic scientific practice when dealing with human participants and are highlighted in the Declaration of Helsinki (World Medical Association, 2013). While drafting a complete and appropriate informed consent is mandatory, extra effort should be placed to ensure that the participant fully understands all these aspects. We found challenges in establishing a fully horizontal dialogue with potential participants as they were often introduced for the first time on the concept of genetic data, and they understandably lacked the foundational knowledge necessary to engage fully, especially concerning data storage and sharing. This challenge is not only due to a disparity in knowledge but also to the perception of our team as “authorities” in the field, inadvertently creating a hierarchical dynamic.

To help build a deeper understanding of the methodology in data generation, analysis, and storage, scientists could work on building long-term partnerships with the communities. Yet, practical barriers such as budget constraints, time limitations, and the disruptive impact of the COVID-19 pandemic impeded the full realization of such a long-term collaboration. One way to build long-term partnerships would be through a consortium to facilitate preliminary education, provide educational materials and resources to community members on topics related to genetics and the research process, and develop capacity building for access to research activity. This educational initiative could include workshops, seminars, and community meetings. Additionally, the sessions would emphasize the potential benefits and risks associated with the research and involve members of other communities not involved in the genomic project to appreciate the broad reach of such genetic studies. Finally, the consortium could elaborate on technical options related to data sharing, storage, and sequencing technology. Similar initiatives are not yet developed in the Global South but are designed as a gold standard in some countries, including the United States, New Zealand, Canada, and Australia (Claw et al., 2018; Garrison et al., 2019; Hudson et al., 2020; Tsosie et al., 2020). In Chile, an example of a long-term academic initiative fostering ethical and inclusive practice on genetic research in indigenous communities is the *Grupo de Estudio Ciencia y Comunidades Originarias* organized by the Center for Intercultural and Indigenous Research and the Pontificia Universidad Católica de Chile (Silva et al., 2022).

While our engagement with the indigenous communities was impactful, it lacked a long-term vision, a limitation often encountered by other scholars (TallBear, 2014; Tsosie et al., 2020). Teaching technical knowledge about genetics was challenging, especially in the absence of established structures for indigenous genomic projects in the Global South. Nonetheless, we aimed to improve the current research model, recognizing that achieving a gold standard was not feasible. Though resources for community-driven efforts have been traditionally scarce or non-existent for most institutions and research funding bodies, our approach was uniquely positioned due to the relatively substantial resources available to us, thanks to the significant funding of our institution (University of Zurich). These resources allowed us to involve local researchers and experts while including cultural protocols and sensitivities. We were able to provide schematic graphics and transparent project steps to develop open and accountable research practices. We could also take time to organize a dedicated trip and undertake the described actions towards the communities. Integrating community engagement as an intrinsic component of the original research proposal should take a higher value in our academic curricula and be supported by an appropriate timeline and budget approved by the institution’s boards. Our experience underscores the need for grant agencies to recognize the importance of such engagement initiatives and allocate resources accordingly. This recognition ensures that the benefits and insights from collaborative endeavors can be maximized for both scientific advances and community empowerment.

Interestingly, the participants often expressed curiosity and enthusiasm for expanding the scope of the study into other areas. A recurring sentiment was encapsulated in the common question, “And now, what else?”. The participants’ eagerness to research further aspects demonstrated the potential for a broader engagement aligned with their interests and questions. However, creating long-term partnerships and facilitating capacity building presented challenges, primarily attributable to the structure of scientific funding and the relative early-career stage of the researchers involved.

## 5 Discussion

To create ethical and sustainable research in the Global South, it is essential to understand the different layers of inequality and the social reality of Latin American societies. In general, academic research is inserted in power dynamics where economic and epistemological asymmetries reproduce colonial attitudes (Argüelles et al., 2022). Addressing epistemic extractivism is crucial, as it perpetuates a power dynamic that takes data and knowledge from marginalized communities without equitable collaboration or benefit-sharing, reinforcing existing inequalities and undermining autonomy (Mc Cartney et al., 2022). On the other hand, academic research can be an agent of positive change, empowering indigenous communities in countries with little or no recognition from the state (Silva et al., 2022). Genuine collaboration and meaningful engagement with local communities increases the value of local knowledge and perspectives in the research process. Creating long-term partnerships and capacity-building initiatives that empower local researchers is essential (Hudson et al., 2020). We acknowledged the limitations of our research approach, as Community-Based Participatory Research should involve continuous, long-term engagement with the community (TallBear, 2014).

More specifically, genetic research in the Global South can have repercussions on the local society and identity. It contributed to stigmatization and perpetuated harmful perspectives, such as mestizo rhetoric or indigenous purism (Garrison et al., 2019; Silva et al., 2022). Genetic admixture from different ethnic backgrounds is an undeniable reality in Latin America. However, it was not a result of voluntary choices but a violent process accompanied by subjugation and stigma, often hidden from the official historical narrative (Galindo, 2020). To move away from these harmful perspectives, we must embrace a more nuanced understanding of genetic admixture in the region (Wade et al., 2014a). Consultation with state institutions and the “mestizo” population is insufficient and will reproduce historical harm (Ávila-Arcos et al., 2022). National or international efforts in genomic research should consider the specific contexts of countries with limited investments and respect indigenous governance structures. Ethical science should be central to budget distribution systems for granting agencies, and researchers, research institutions, and indigenous communities should collaboratively develop guidelines (Argüelles et al., 2022). There is a growing number of genomic projects in Latin American countries managed by local researchers, like Latin Cells, CANDELA, DNAdoBrasil, INMEGEN, and ChileGenómico, and a growing interest in the indigenous history of the continent. However, indigenous participation is still scarce compared to North America (Claw et al., 2018; Tsosie et al., 2020).

Community engagement has been our primary focus for supporting ethical and inclusive research in our genetic study of Mapuche ancestry in Chile (Arango-Isaza et al., 2023). Here, we presented our strategies to report research to the Mapuche communities and involve their perspectives in the genetic study. Garrison et al., 2019 emphasized the importance of giving back results in genomic research when involving indigenous communities or minorities, but this action is not guaranteed enough in scientific studies. These dialogues were designed to bridge the gap between different epistemologies and change societal perspectives on issues like racism (Haverkort, 2013; Wade et al., 2015). We also evaluated the qualitative impact of our collaborative and participatory efforts to demonstrate the potential for meaningful and more reciprocal engagement with the local communities. By incorporating comments and suggestions, the scientific paper benefitted from more rigorous data interpretation. Furthermore, we addressed economic and epistemological asymmetries to build a more horizontal collaboration and enhance the integrity of the scientific knowledge production process.

Our experience of engagement with the indigenous communities can contribute to the challenging task of establishing guidelines for genomic research in Latin America. Based on the challenges we faced and on the feedback received, we can share our recommendations for effective and sustainable community partnerships.

• Understand the country’s current and past social and political context, not only from an anthropological or genetic point of view.
• Comprehend the social and power dynamics between the diverse demographics in Latin America, which may not directly align with the indigenous communities.
• Educate yourself about different knowledge systems, including indigenous ones, to appreciate the diverse perspectives and worldviews within the community.
• Develop a deep understanding of the pressing issues the communities face, such as challenges related to education, land rights, and access to essential services, to ensure that the research is contextually relevant and beneficial to the community.
• In the Latin American context, where colonial structures often persist, a non-indigenous university board’s approval of a research permit alone will not exempt a researcher from validation with local communities and stakeholders. Mere acceptance by the mestizo population is not an adequate measure of ethical engagement.
• Emphasize the importance of speaking the local language or the language most commonly used within the community.
• Provide comprehensive explanations of biological concepts during data collection and results dissemination to ensure a thorough understanding of the implications and potential risks involved. Develop teaching and scientific outreach strategies.
• Work towards long-term partnerships and empowerment of the communities.
• Acknowledge common misconceptions and prejudices to provide an educated response if these issues arise during conversation.
• Recognize the significance of local collaborators and stakeholders in ensuring culturally sensitive and meaningful research engagement.
• Create a safe and inclusive environment for discussion and exchange, taking into account the community’s communication styles and preferred methods of debate.
• Be prepared for potential negative responses and the possibility that the community may not wish for the research to be published or to disclose certain aspects of it. Approach framing and dissemination of results with sensitivity and respect.
• Establish mechanisms to deliver research findings to direct participants and a broader audience, including schools, cultural centers, or organized communities.
• Prioritize sharing research results with the community before the publication of the manuscript to ensure transparency and mutual understanding.

In conclusion, there are still several concerns for which the scientific community must actively improve the ethical standards of research, especially in the Global South. This includes fostering collaborations with local researchers, meaningful community consultation, and respecting principles of informed consent and data sovereignty. By involving indigenous communities as active partners and incorporating their perspectives, researchers can conduct genomic research that respects the communities’ rights, values, and aspirations. The international community, especially in the Global North, is responsible for approaching research respectfully, considering their inherited privilege and background.
