# Supplementary material for: Bridging the gap: returning genetic results to indigenous communities in Latin America
Source: Front Genet. 2023 Nov 28;14:1304974. doi: 10.3389/fgene.2023.1304974 (PMC10715051; doi:10.3389/fgene.2023.1304974)
Supplement: Supplementary file 1 [file Presentation1.pdf]

Rasgos genéticos y lingüísticos de la prehistoria de Chile

Universität Zürich[UZH]

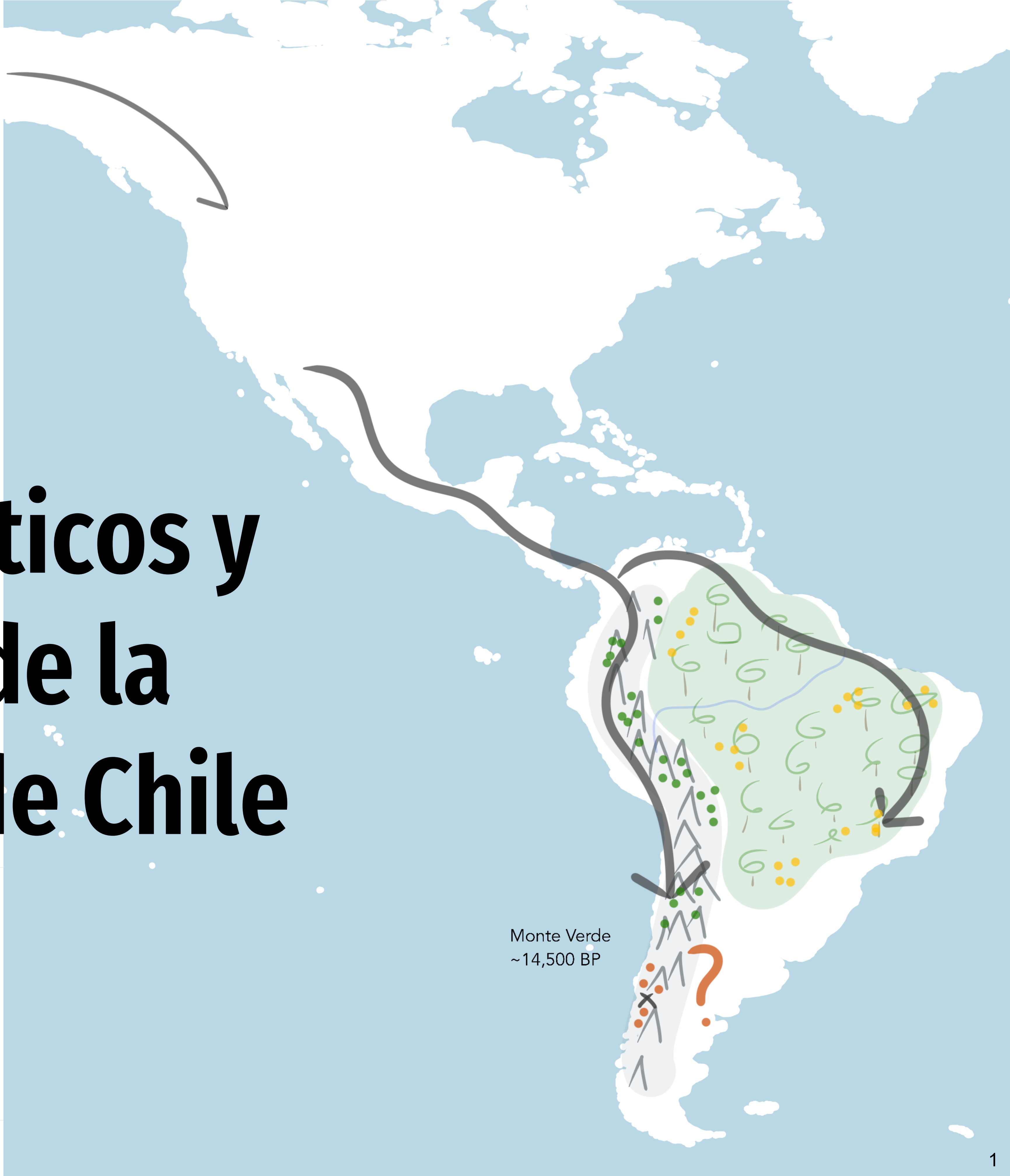

¿Qué es la genética?

- La genética es el área de estudio de la biología que busca comprender cómo se transmite la herencia biológica de generación en generación mediante el ADN.

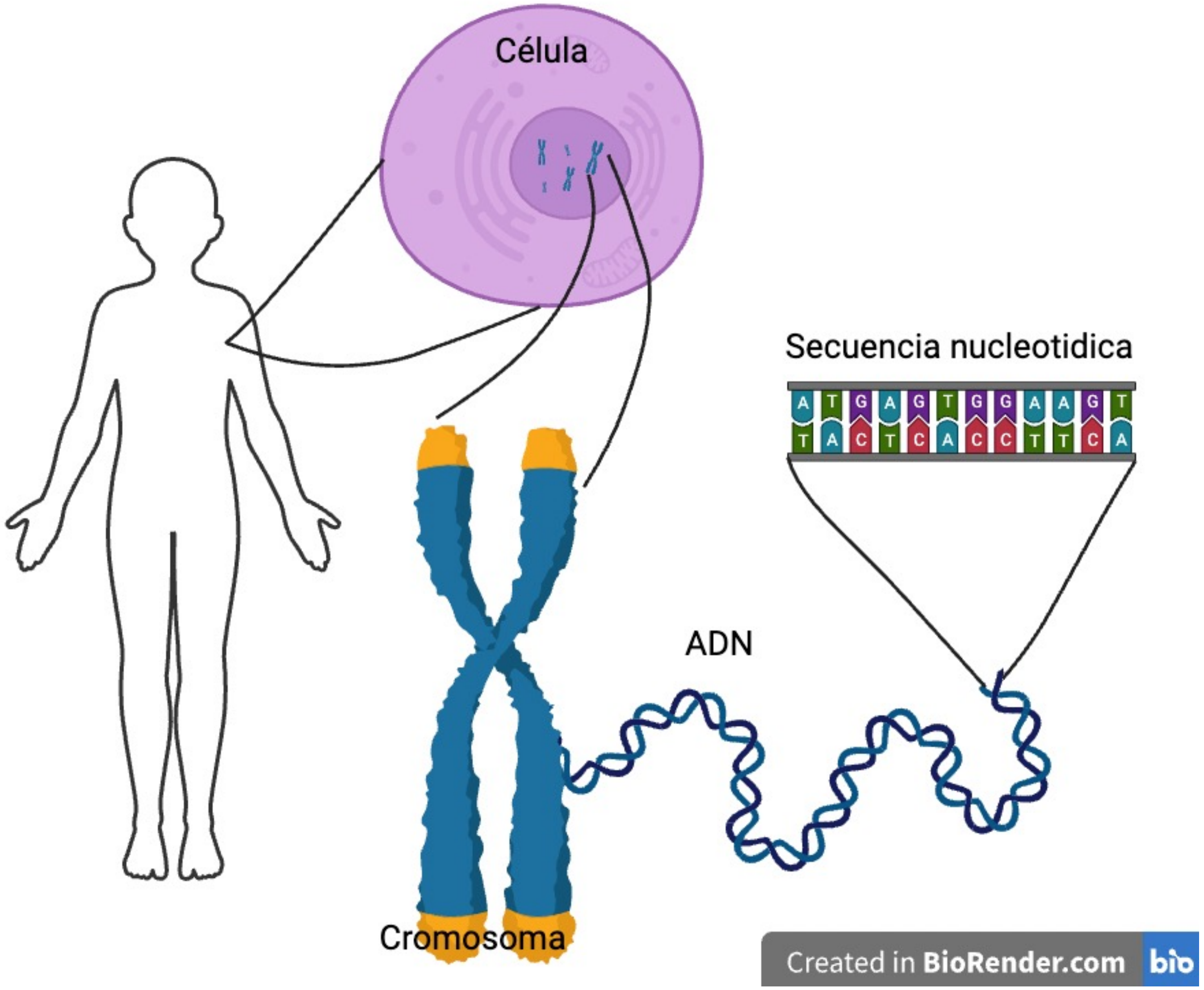

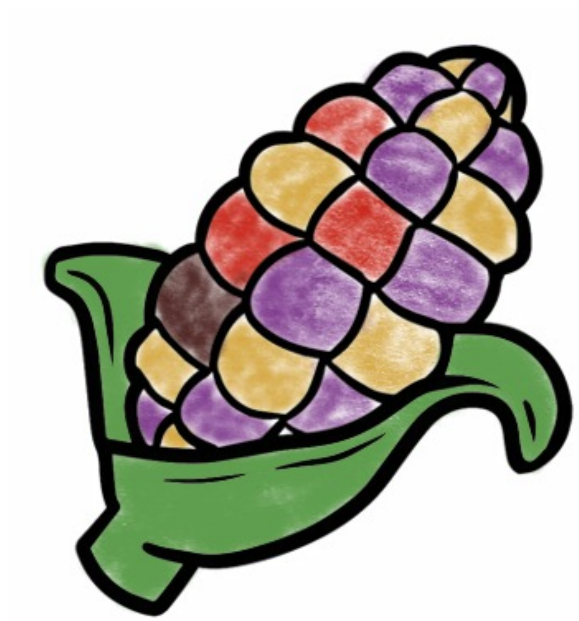

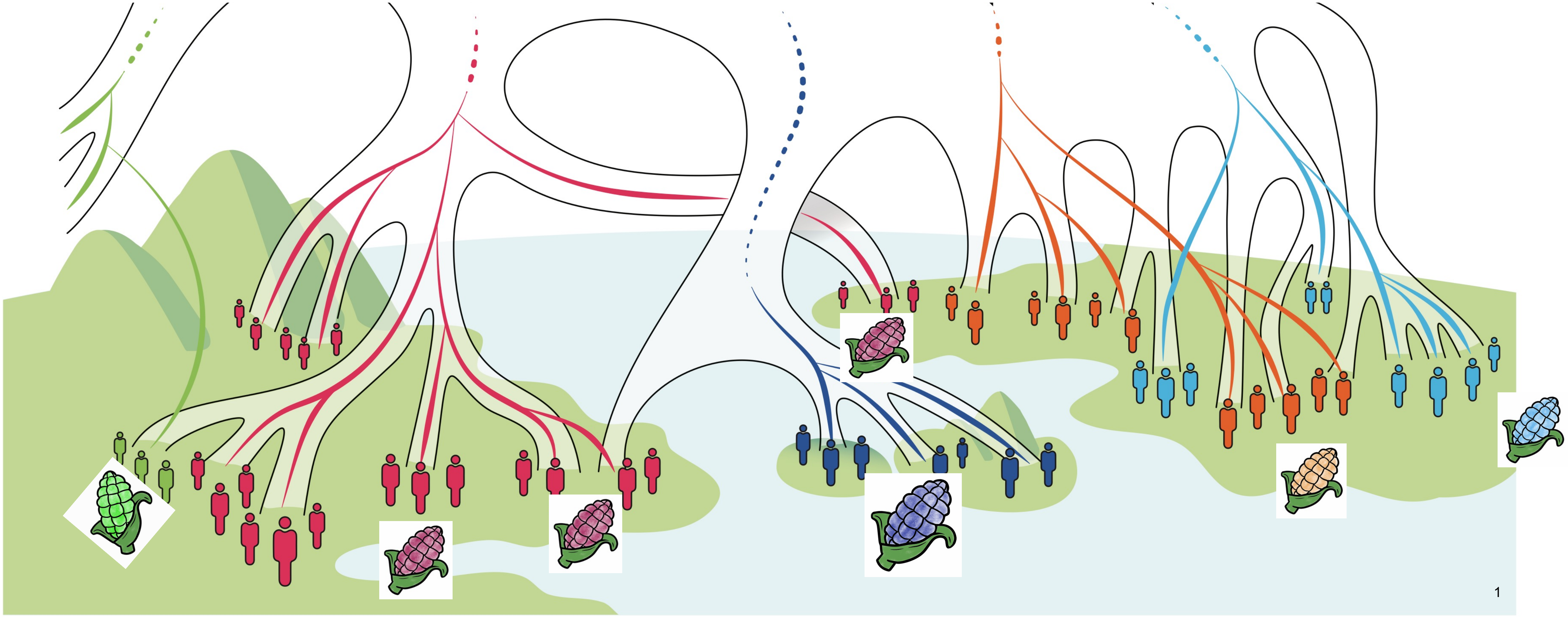
1

¿Cómo funciona el ADN?

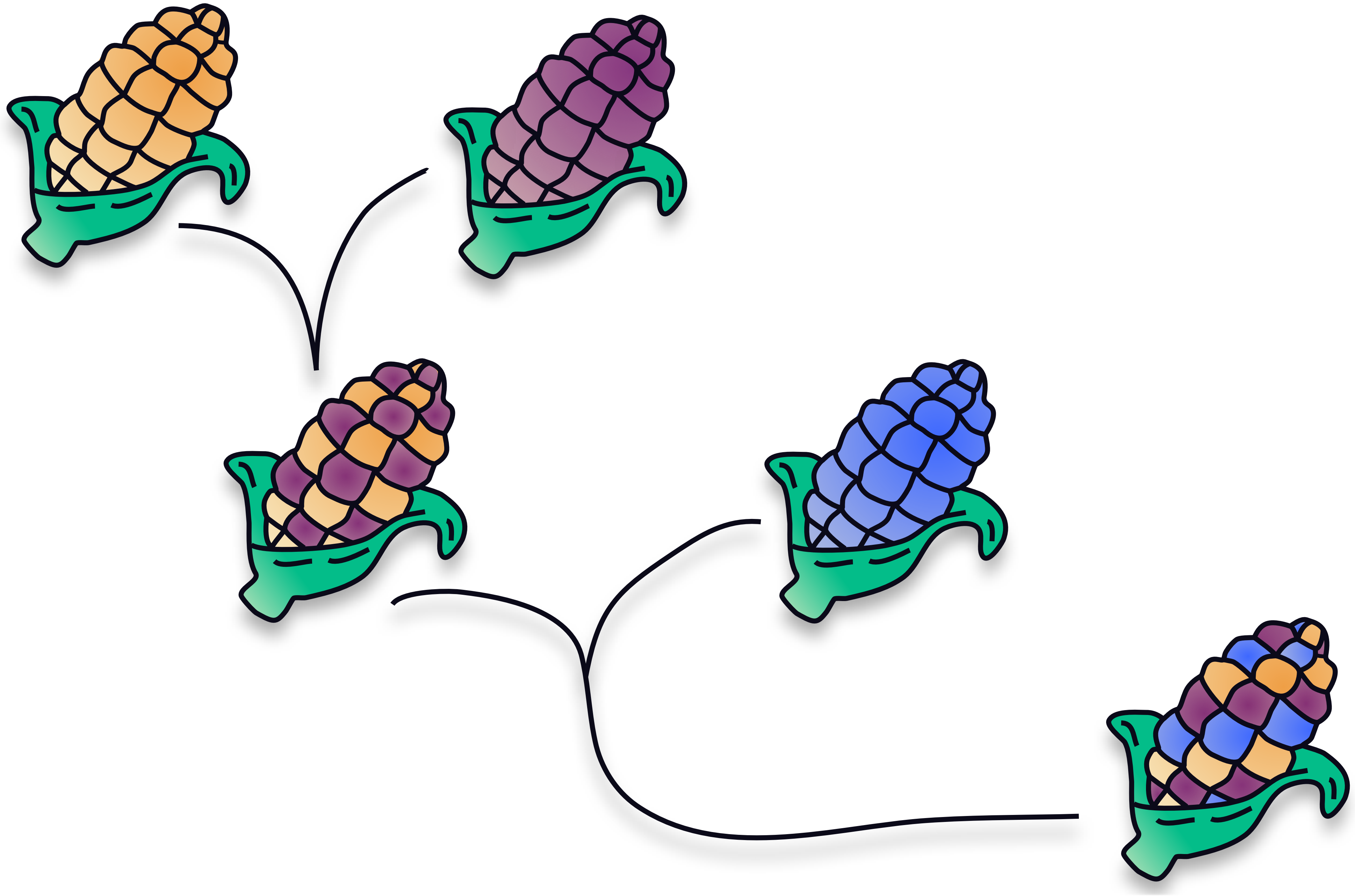

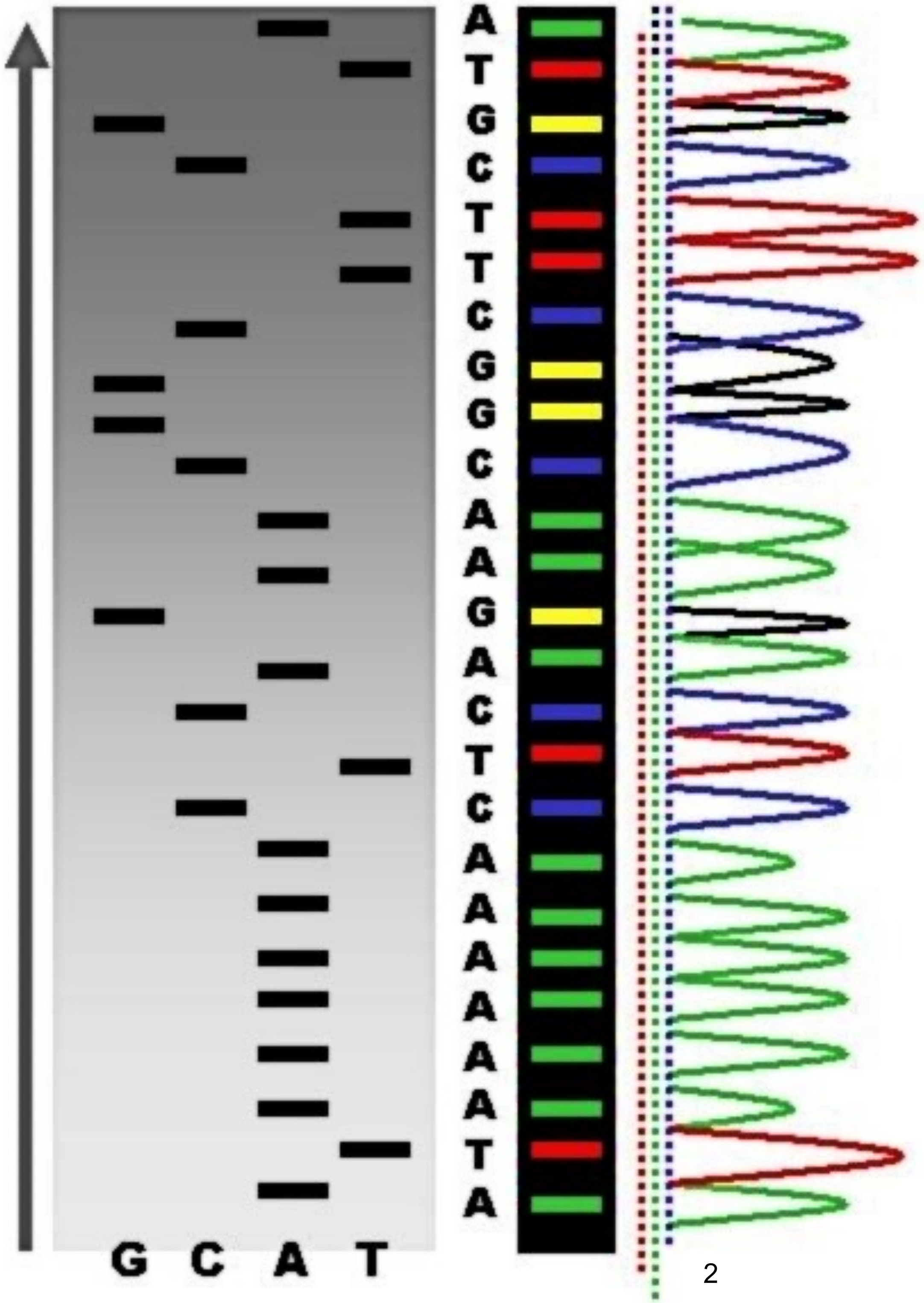

Secuenciación de ADN

Conocimiento

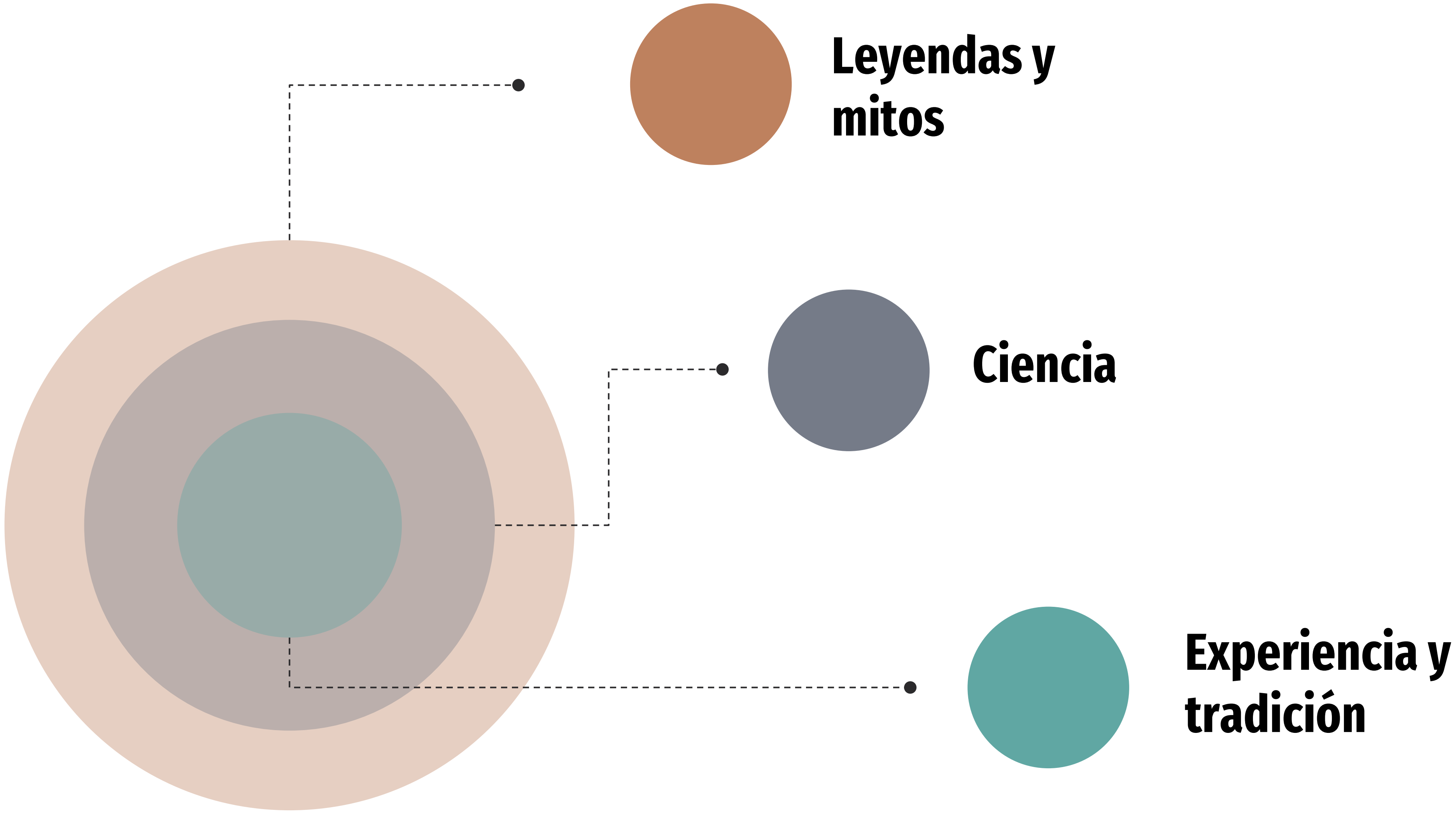

¿ Qué otras datos utlizamos para los análisis?

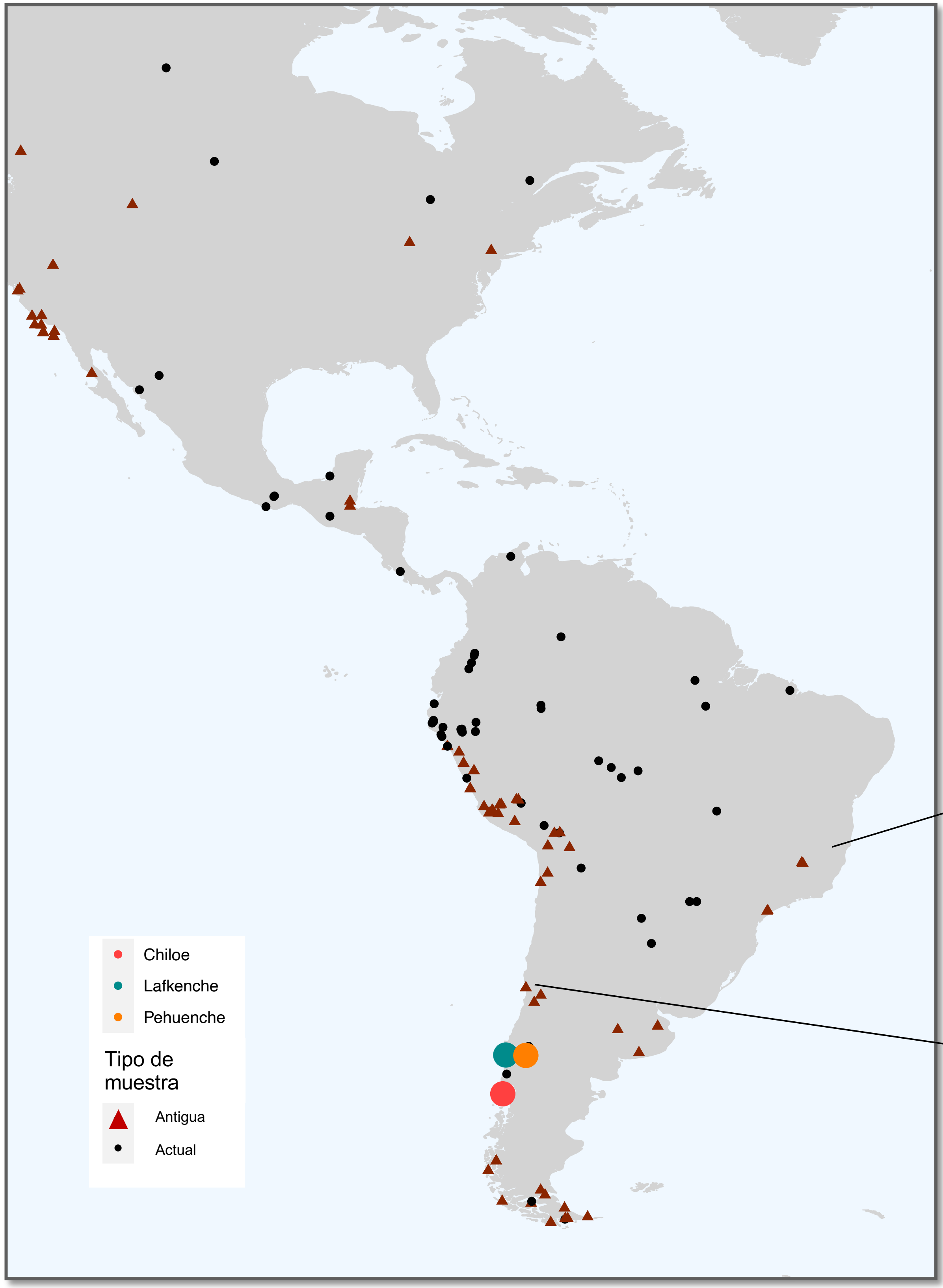

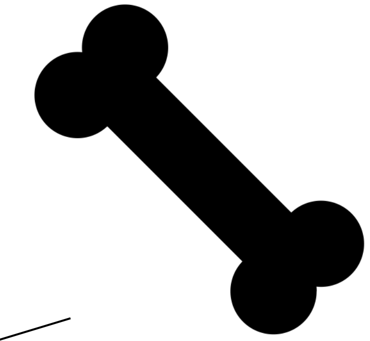

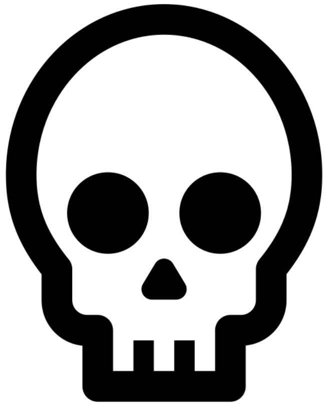

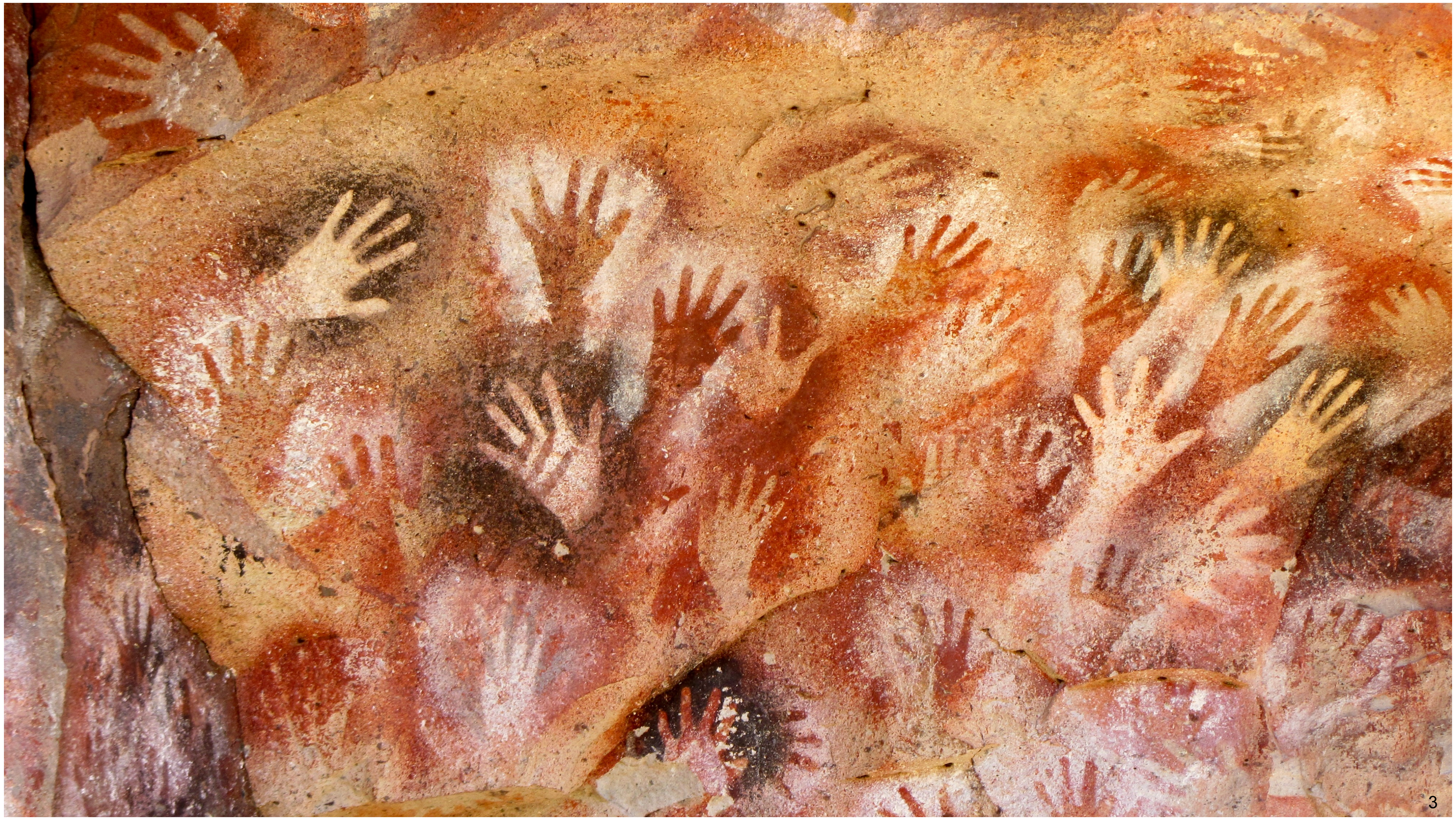

Cuevas de las Manos, Santa Cruz, Argentina

Escala temporal

01
Pleistoceno tardío/ Holoceno temprano
15,000 – 8,500 años

02
Holoceno medio
8,500 – 3,500 años

03
Holoceno tardío
3,500 años

Presente

Historia del poblamiento de las Américas

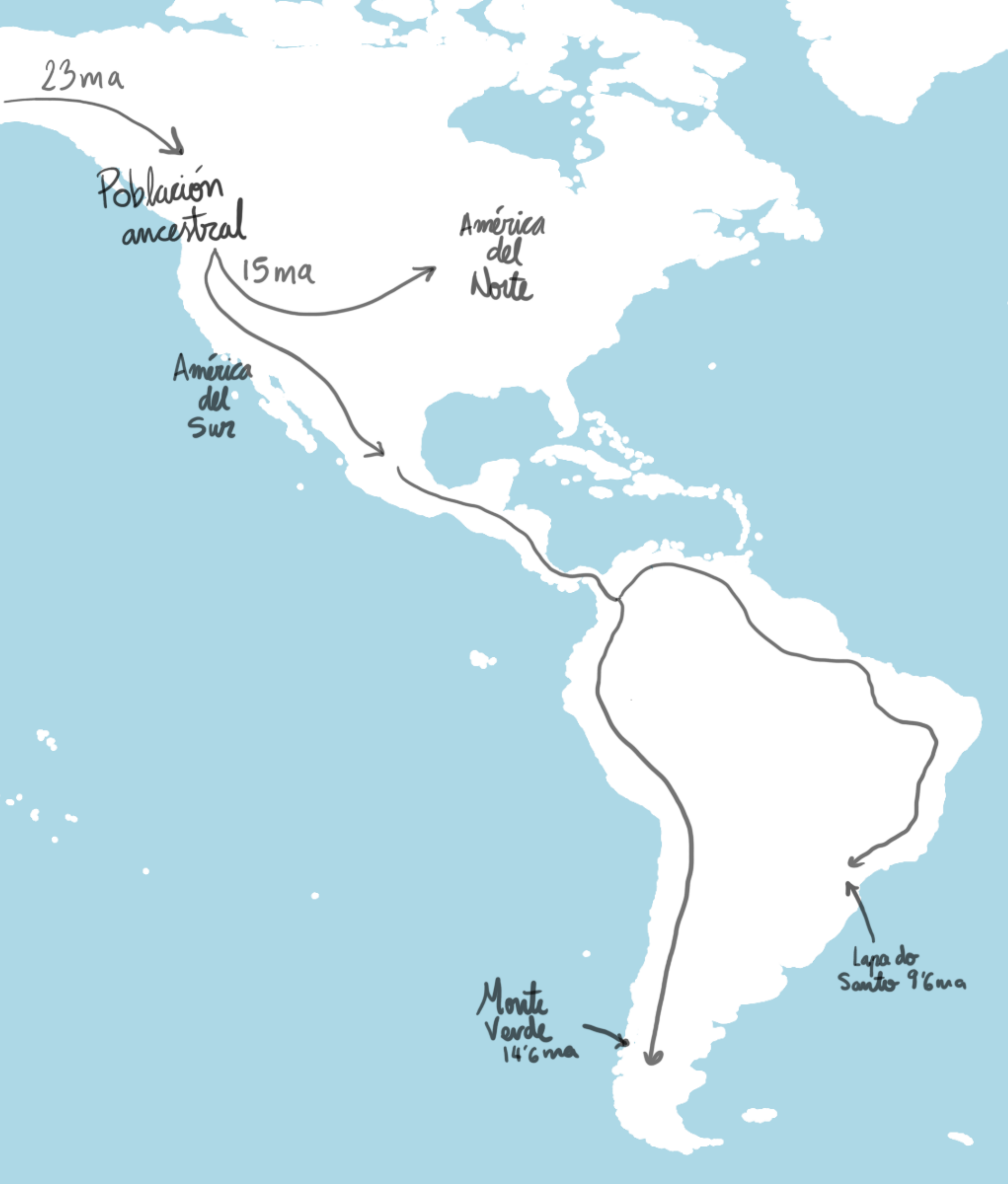

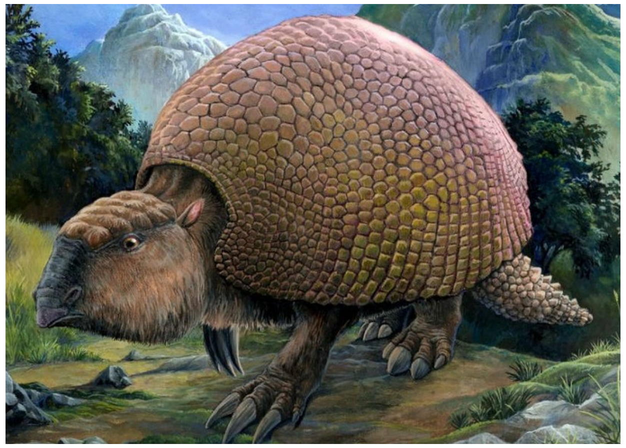
4
Gliptodonte

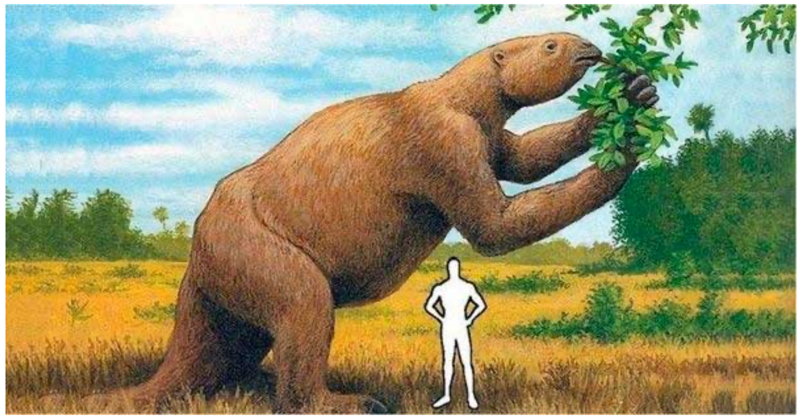
5
Perezoso gigante

01. Pleistoceno tardío/Holoceno temprano (15,000 - 8,500 años)

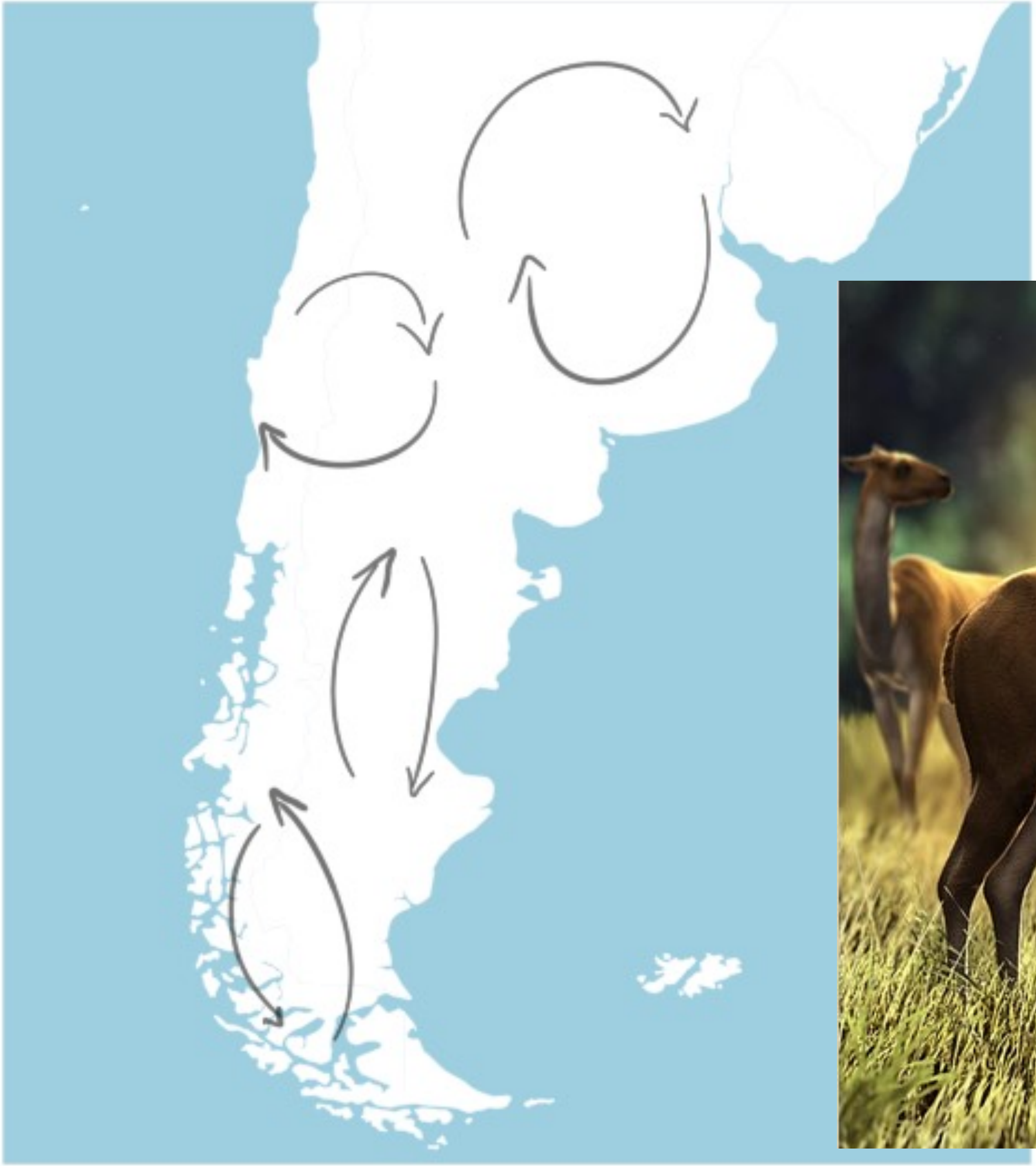

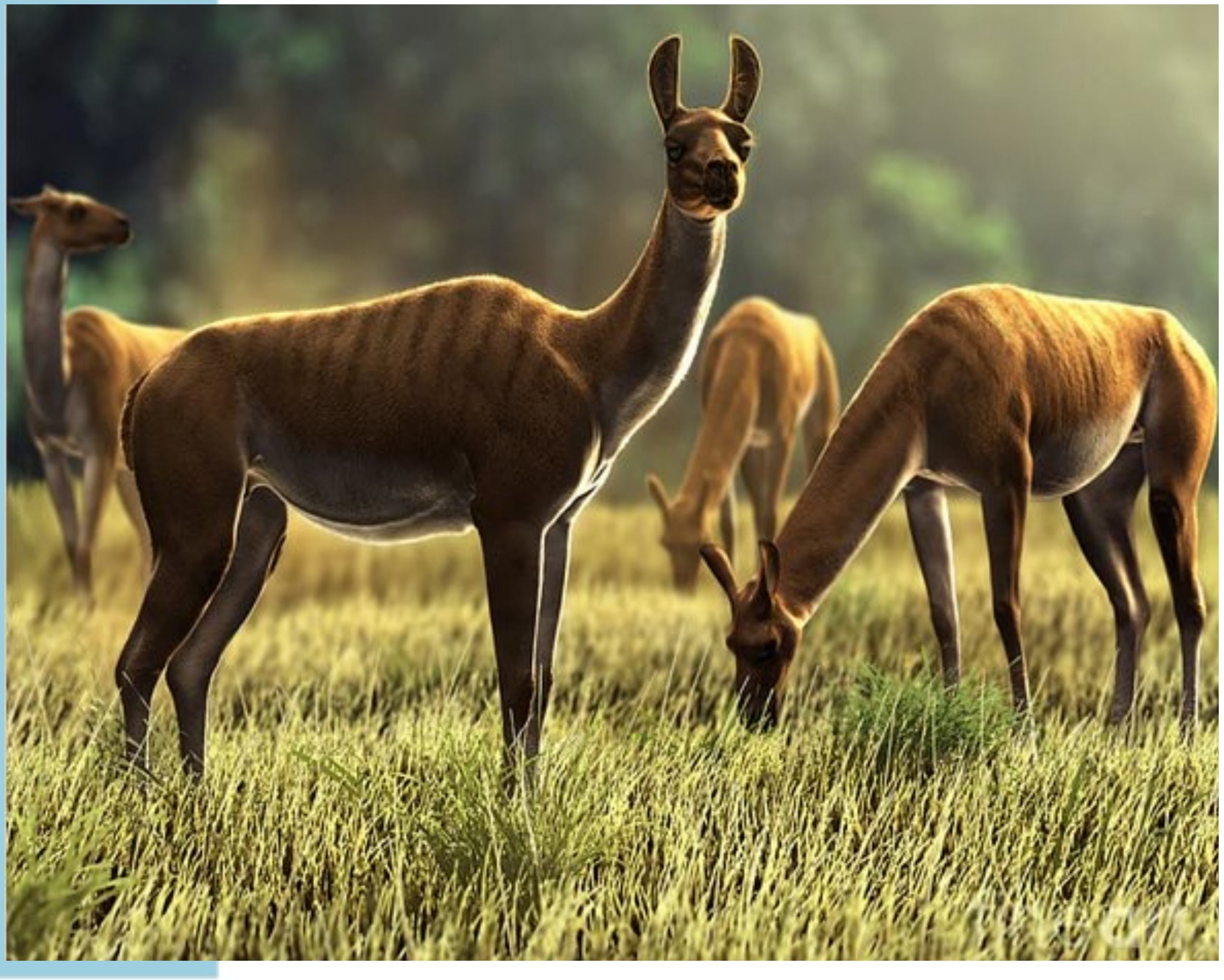

Paleolama

6

01. Pleistoceno tardío/Holoceno temprano (15,000 - 8,500 años)

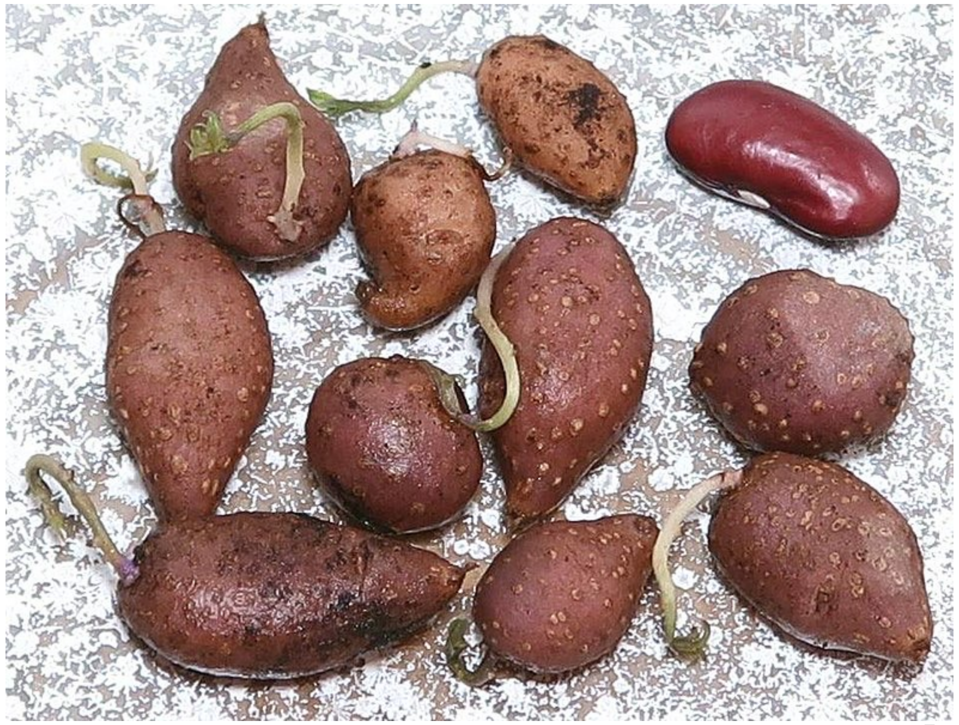

7

Papa salvaje

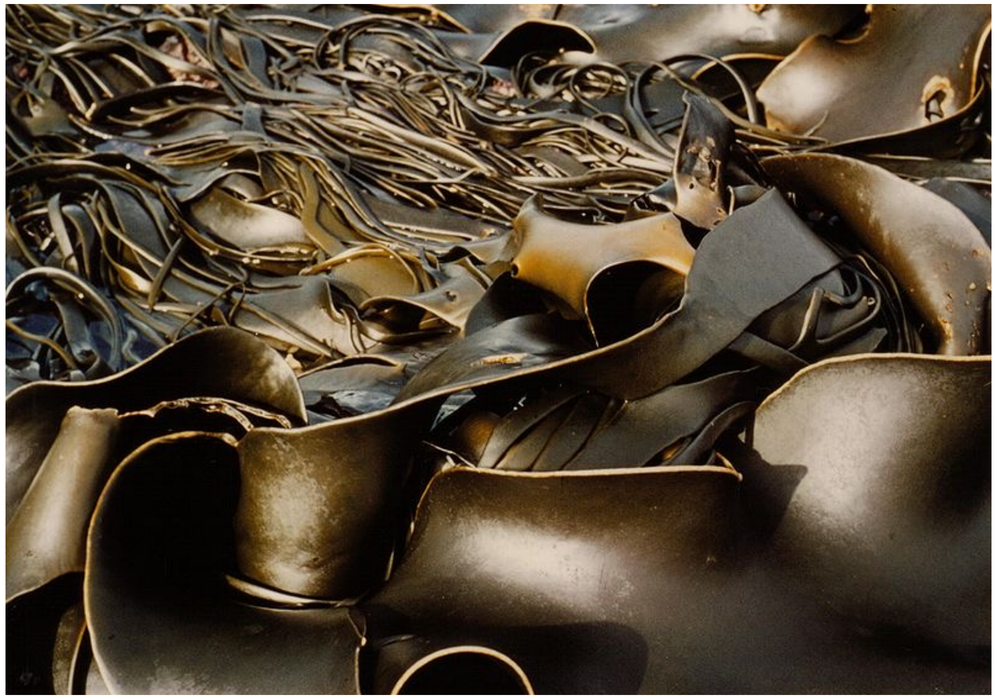

Cochayuyo

8

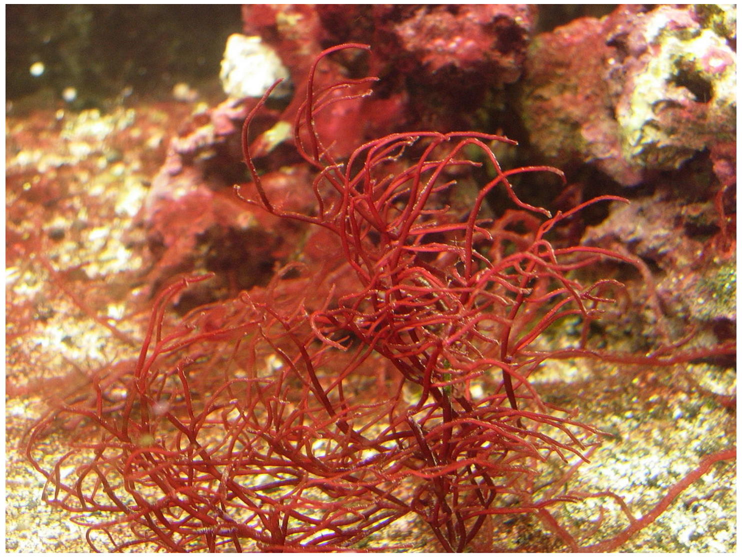

Pelillo

10

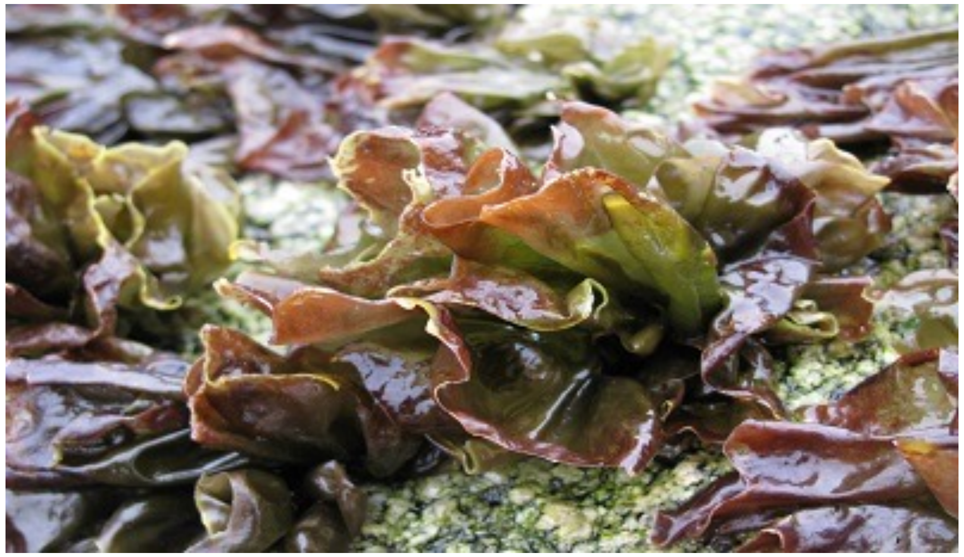

Luche

9

02. Holoceno medio (8,500 -3,500 años)

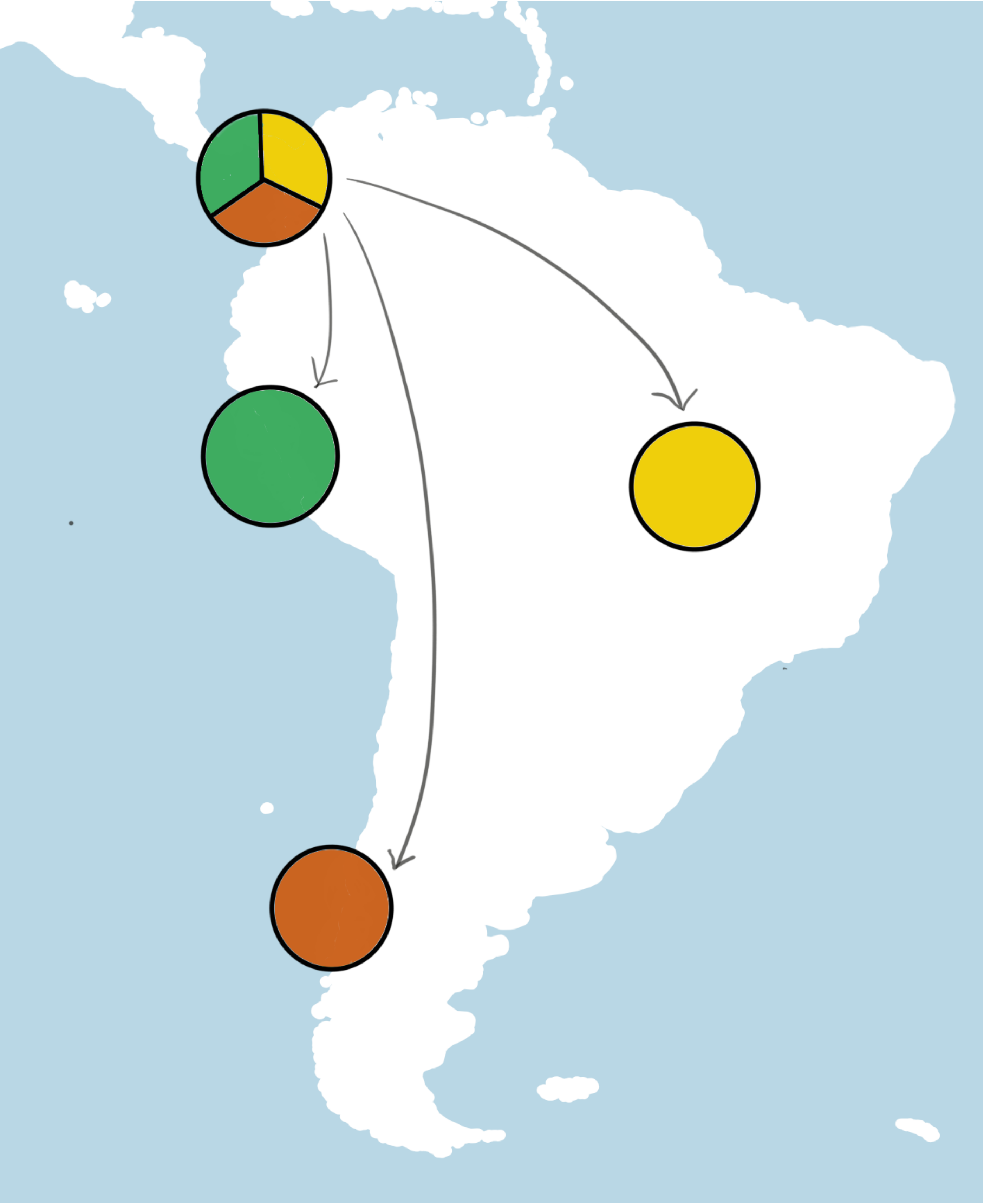

- Mejores condiciones climáticas

- Desarrollo de :
  - Lenguas
  - Cultura
  - Costumbres

¿ Y en el Cono Sur?

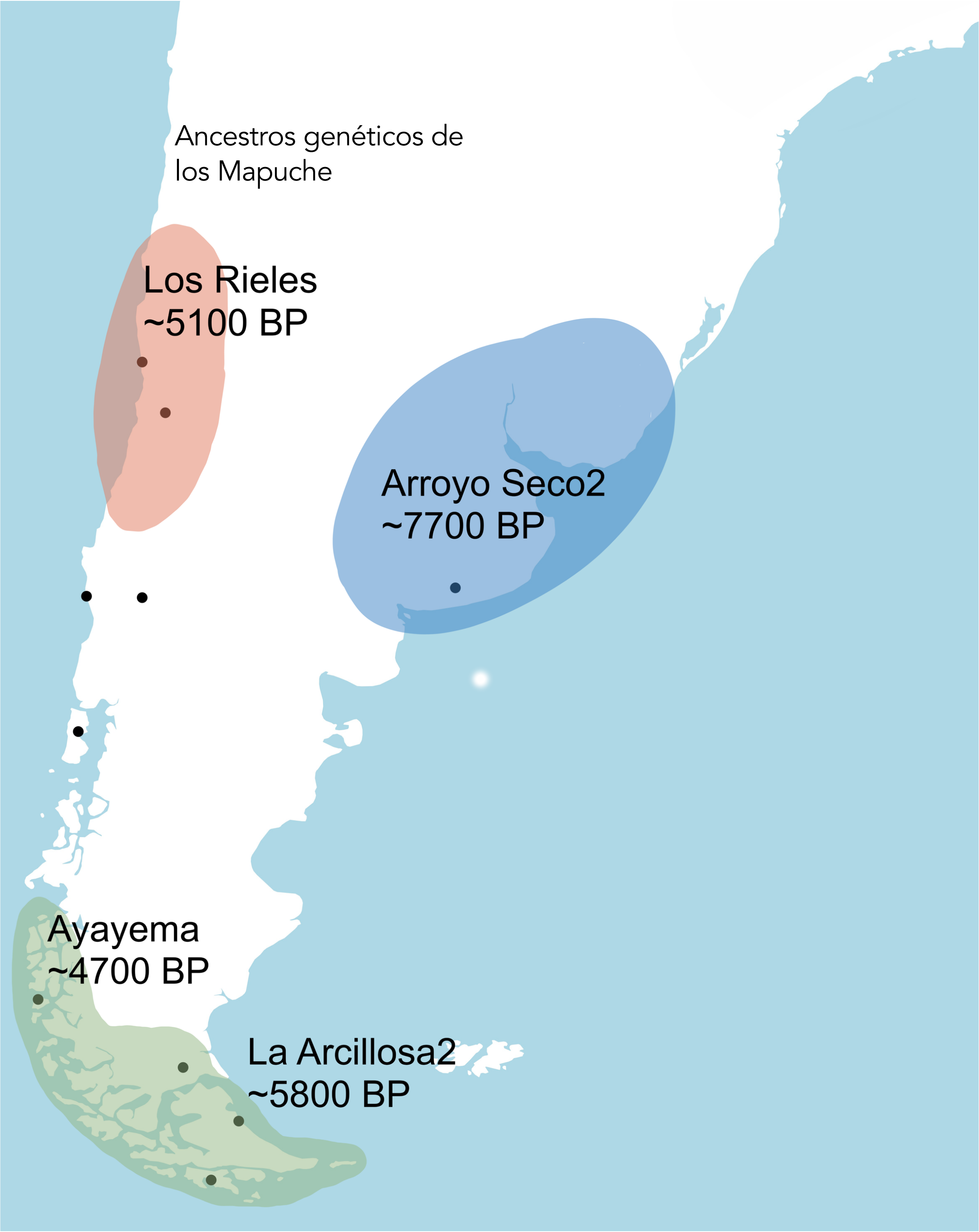

- Ya están presentes los ancestros genéticos directos de las poblaciones del cono sur

- Vinculación de las poblaciones a los diferentes territorios

Migraciones hacia el sur

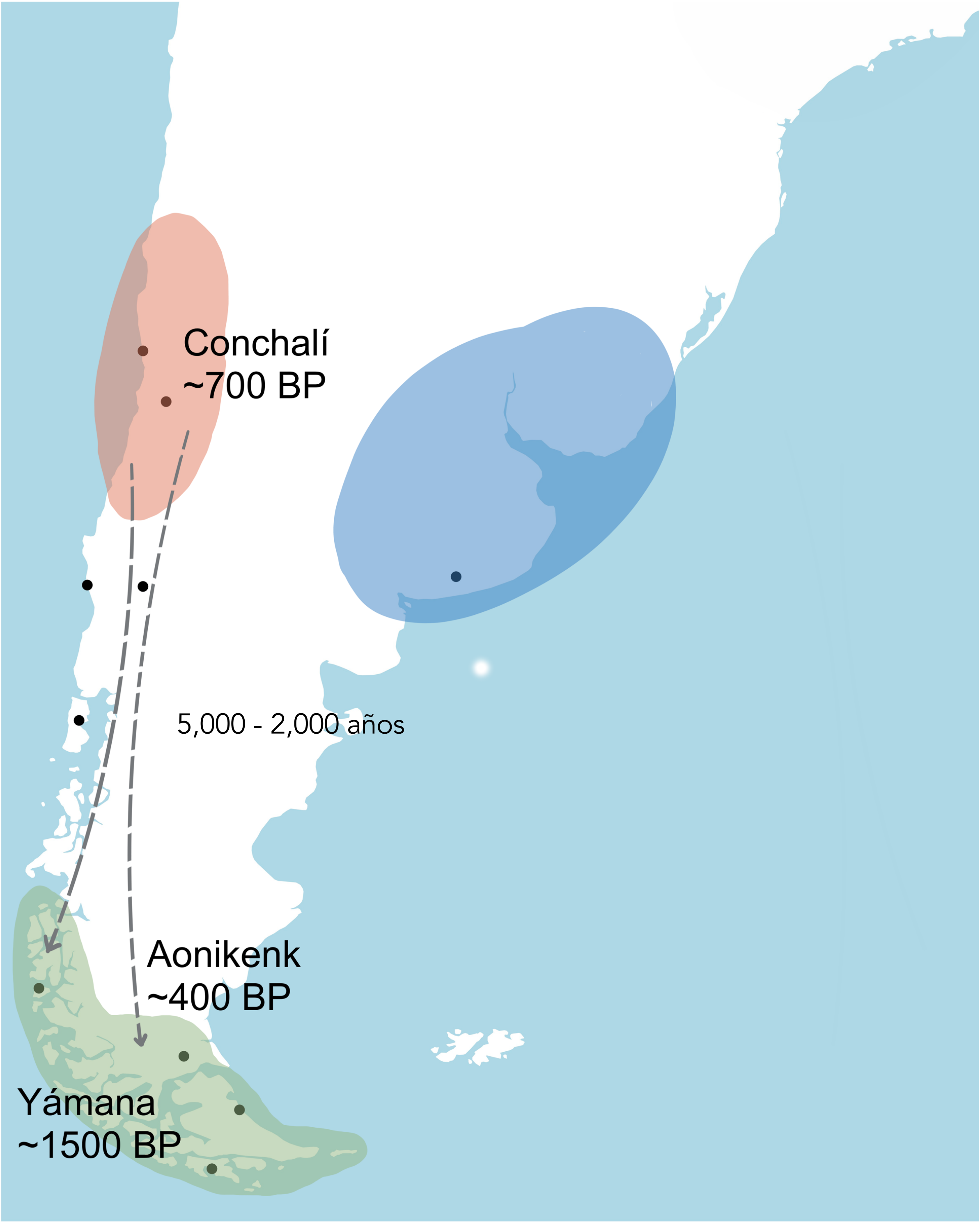

- Ancestros genéticos de los Mapuche migran hacia el sur
- Dejan su legado genético en los pueblos más al sur de la Patagonia
- No hemos registrado migracines hacia el este o hacia el norte en ese periodo de tiempo.

03. Holoceno tardío (3,500 -1,000 años)

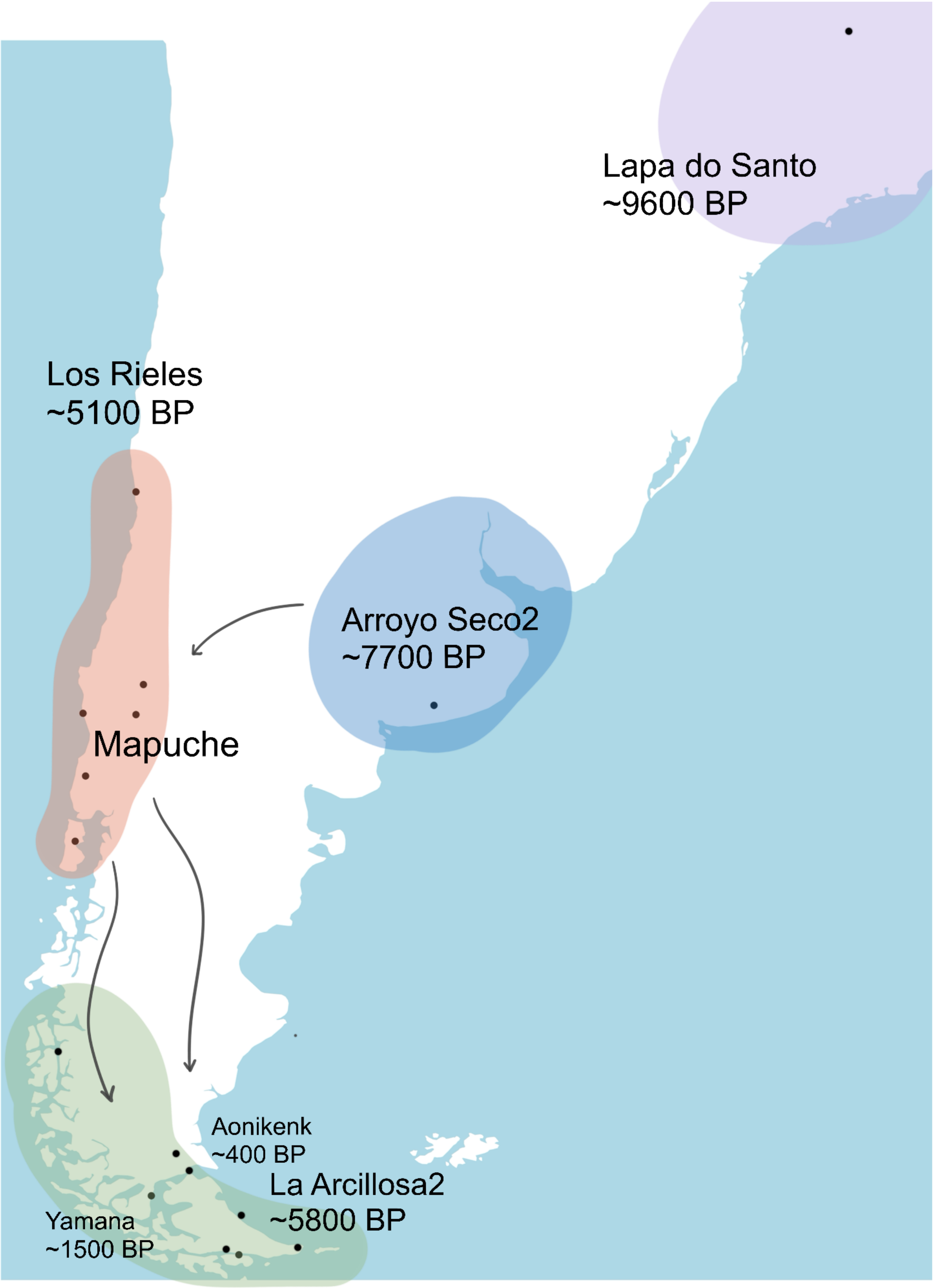

- Las poblaciones se diversifican
- Solidifican diferencias culturales, genéticas y lingüísticas
- Contacto estrecho entre población Mapuche y Tehuelche/Aonikenk
- Los grupos Mapuche a su vez se dividen en otras poblaciones que comparten lengua y costumbres parecidas

Pasado reciente

hace 1.000 años (según la genética)

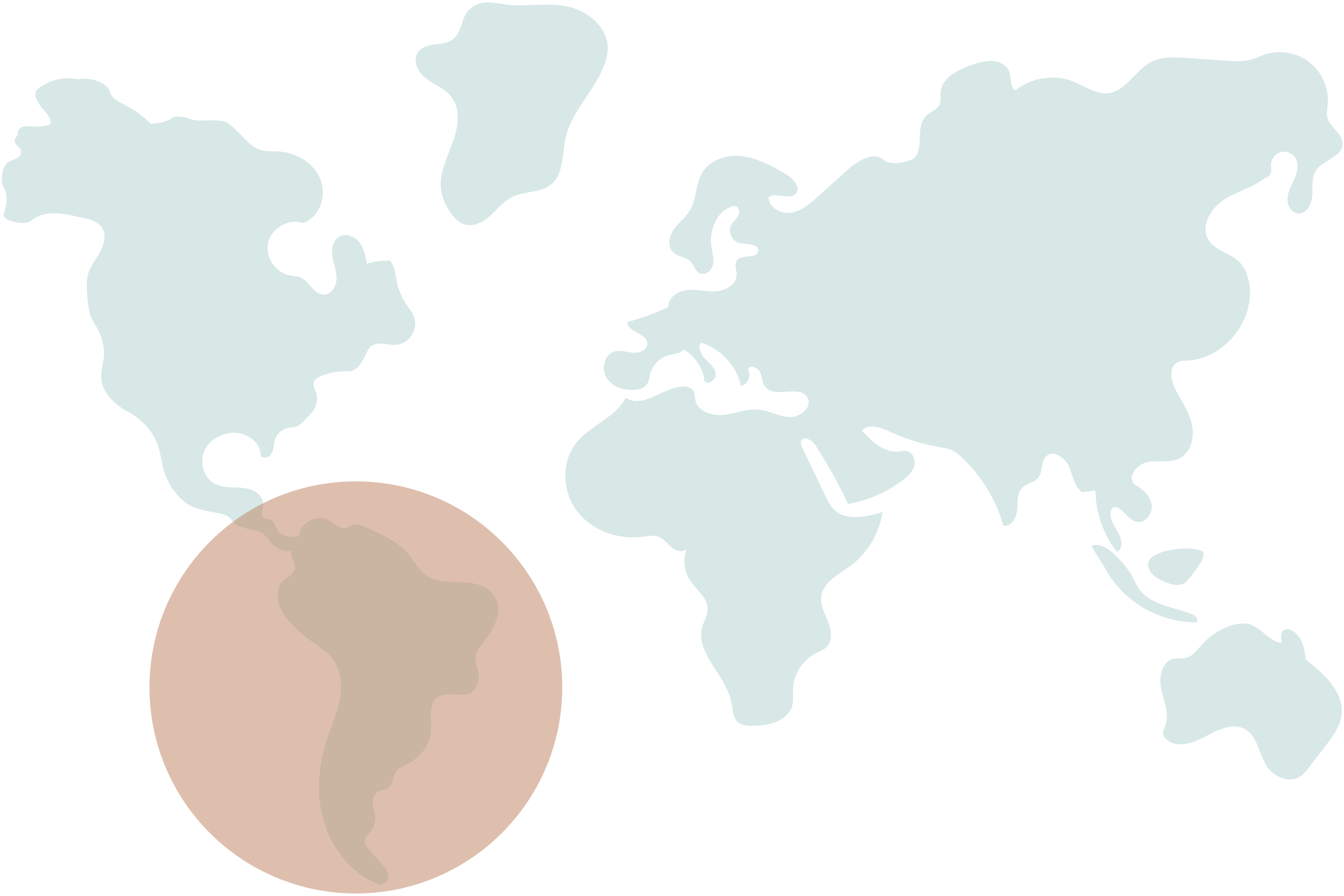

Relación con otras poblaciones suramericanas

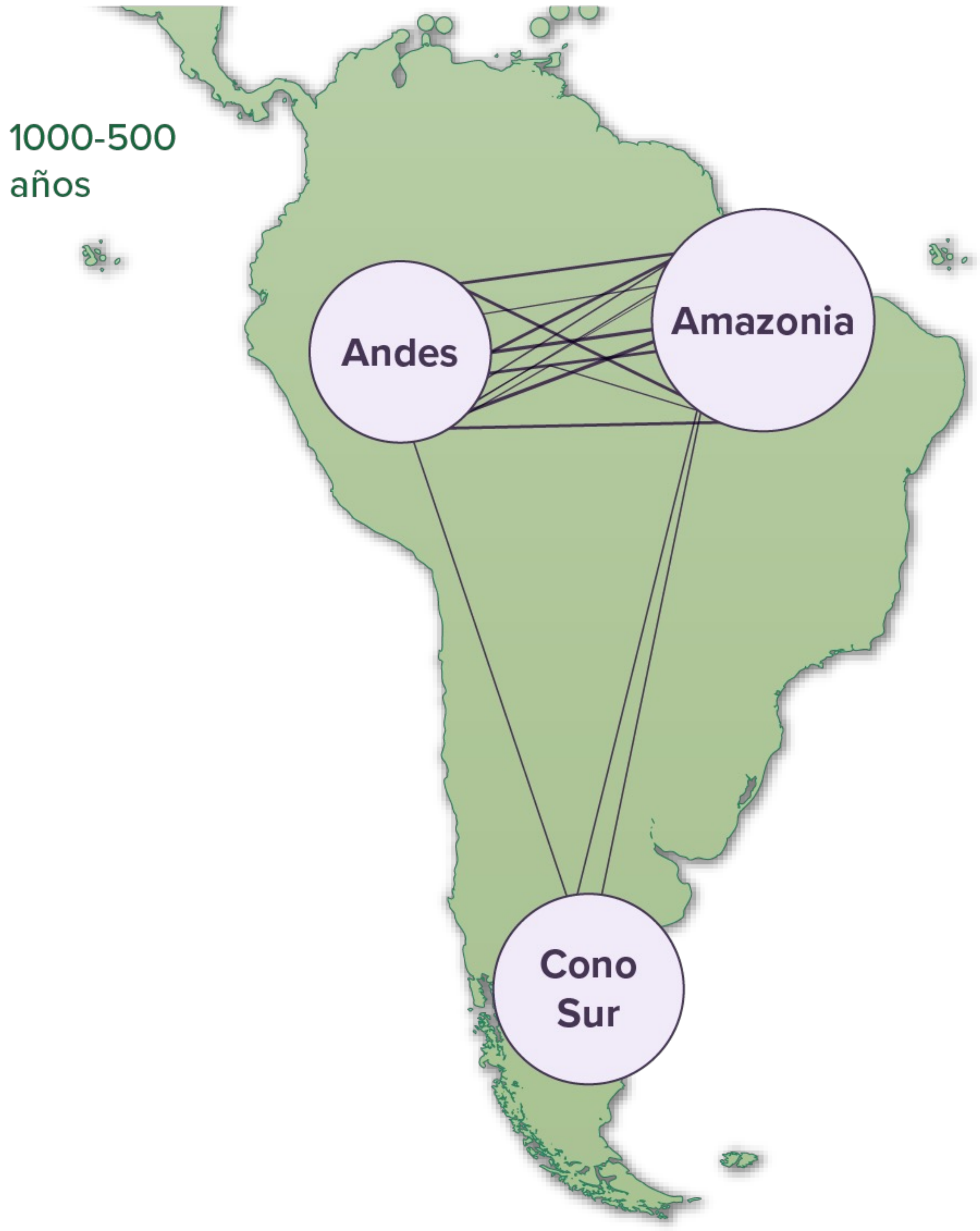

- Obervamos de nuevo las tres grandes áreas del contiente

- El cono sur presenta también algunas conexiones como:

    - Mapuche con los Andes
    - Pehuenche con el gran Chaco
    - Huilliche con el sur de la Patagonia

- Aunque diríamos que es una región más aislada

Contacto con los Andes

- Gracias a la genética sabemos que el contacto es anterior al Imperio Inca.
- No tienen un mismo origen (ni genético, ni lingüístico)
  - Pero vemos conexiones genéticas
  - Y prestamos de palabras

**ampin /ampin/. (De hampi)**	Medicina o remedio	
**awka /au̯ka/ (De awqa)**	Enemigo	
**michi /miči/. (De michi)**	Gato	
**challwa /čaʎwa/. (De challwa)**	Pez o pescado	
**kuchi /kuči/. (De khuchi)**	Puerco, cerdo o marrano	

Contacto con los Andes

- Batalla del Maule, 1485
- Deriva en una influencia mayor del imperio Inca en el norte de Chile
- La red caminera del Tahuantinsuyo

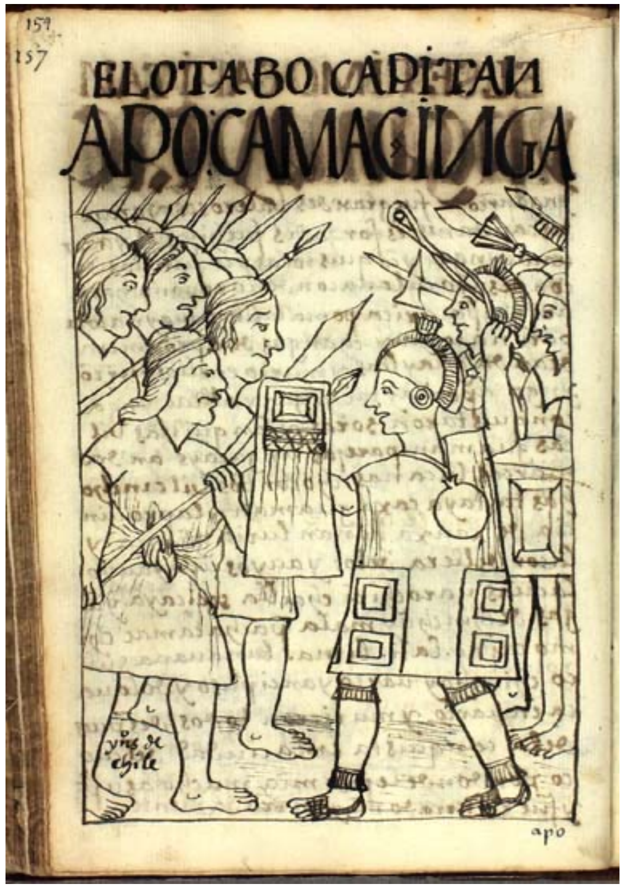

11

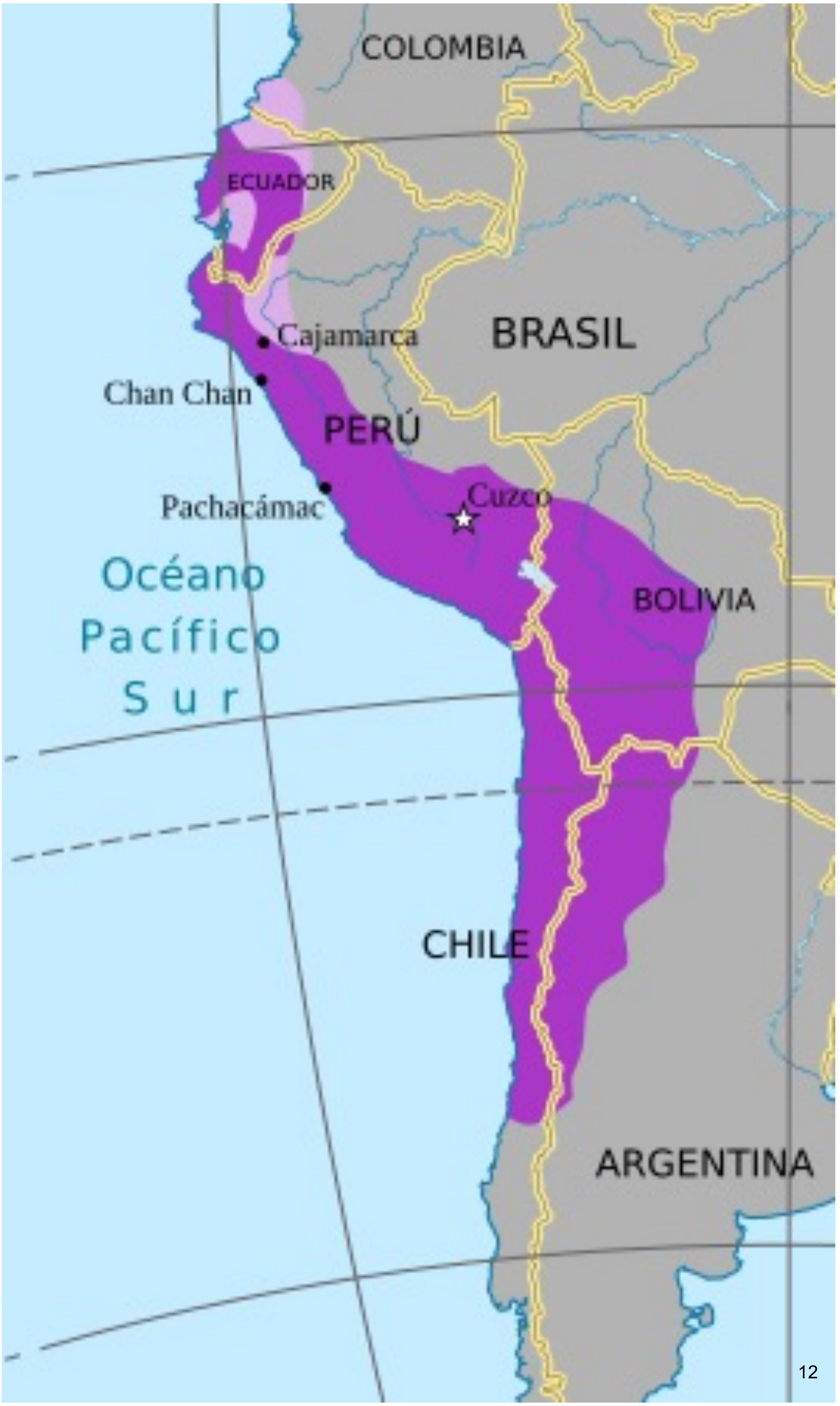

Imperio Incaico/ Tawantinsuyo

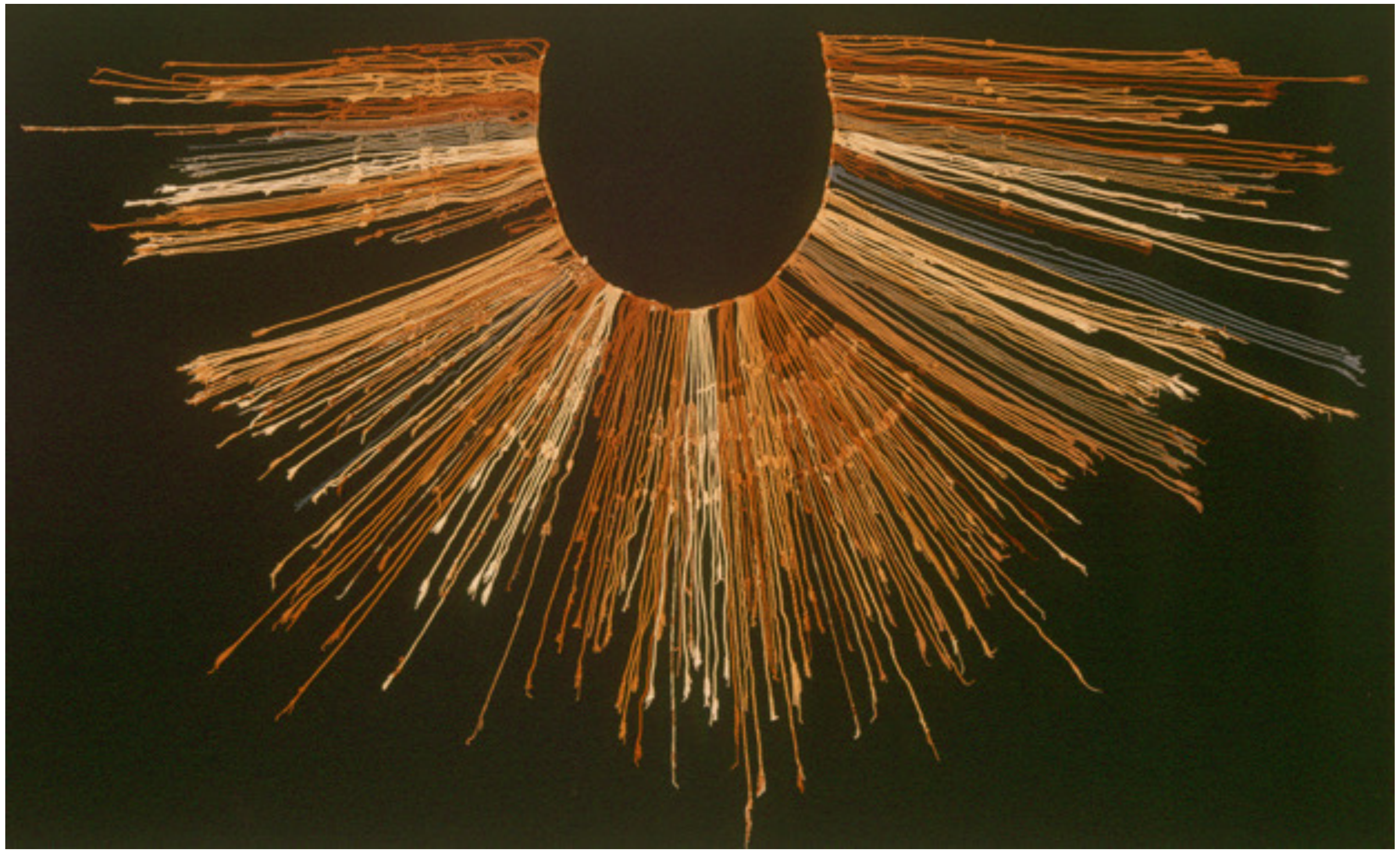

13

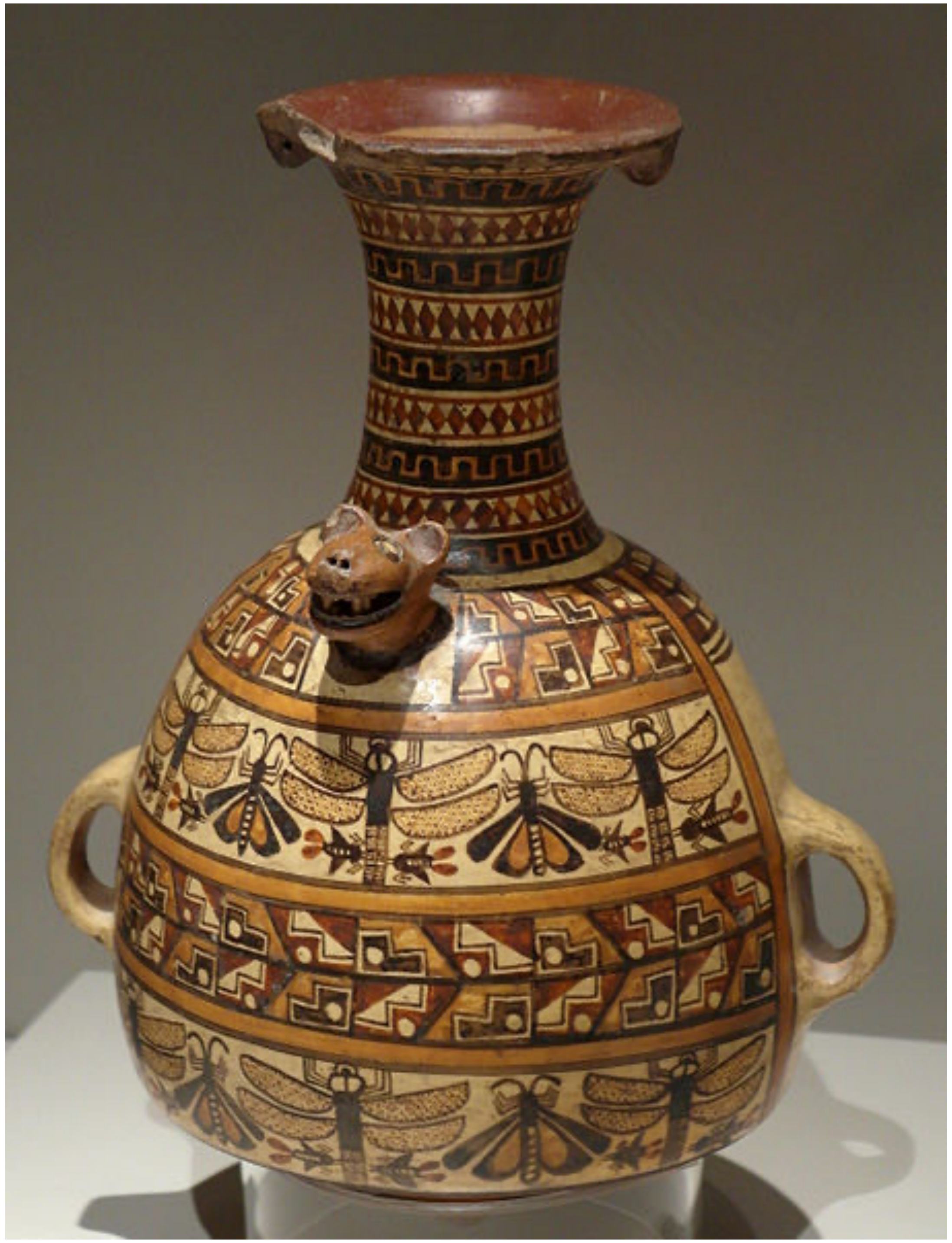

14

Relación entre los pueblos Mapuche

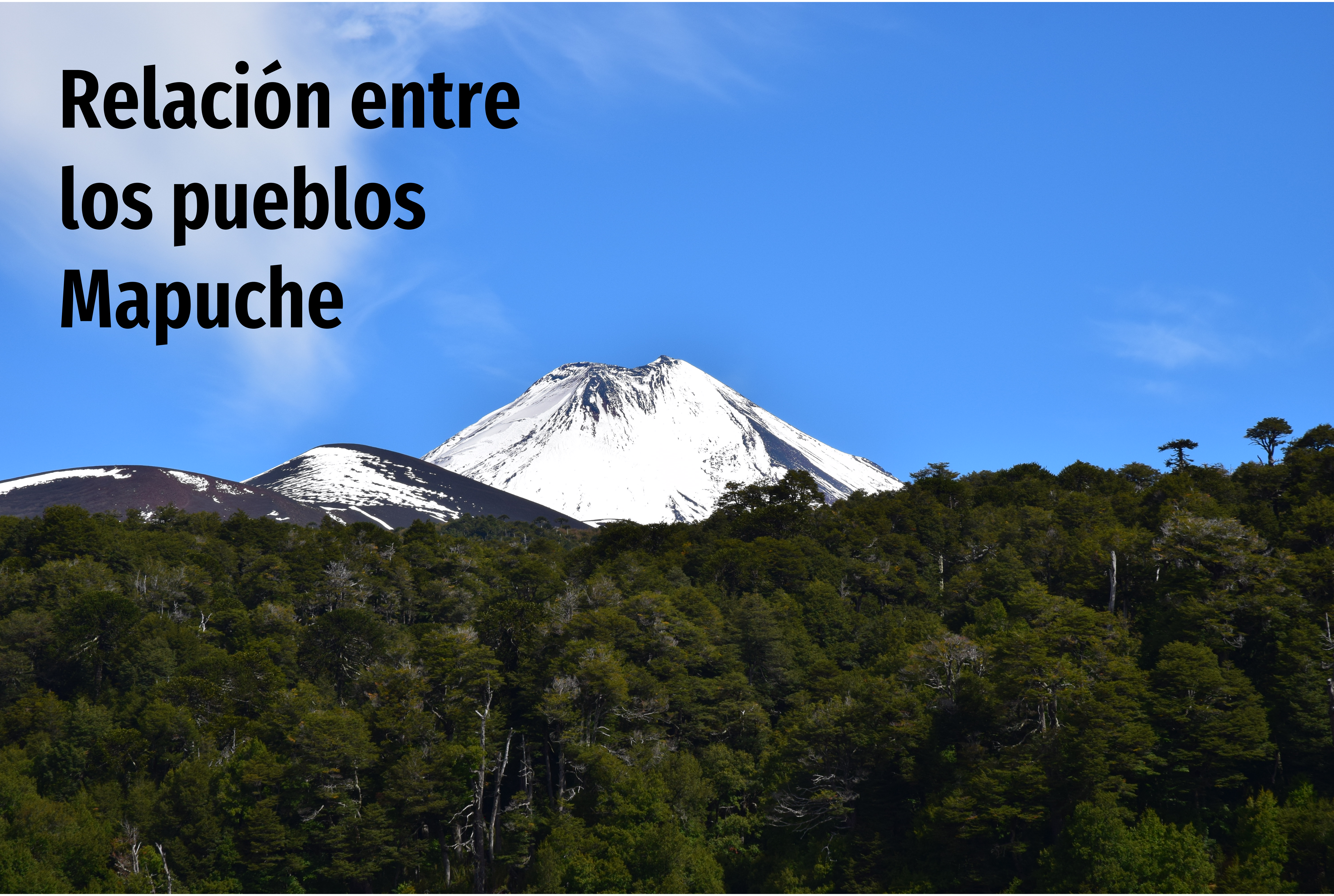

Credit: Epifanía Arango-Isaza

Comparten la misma historia genética

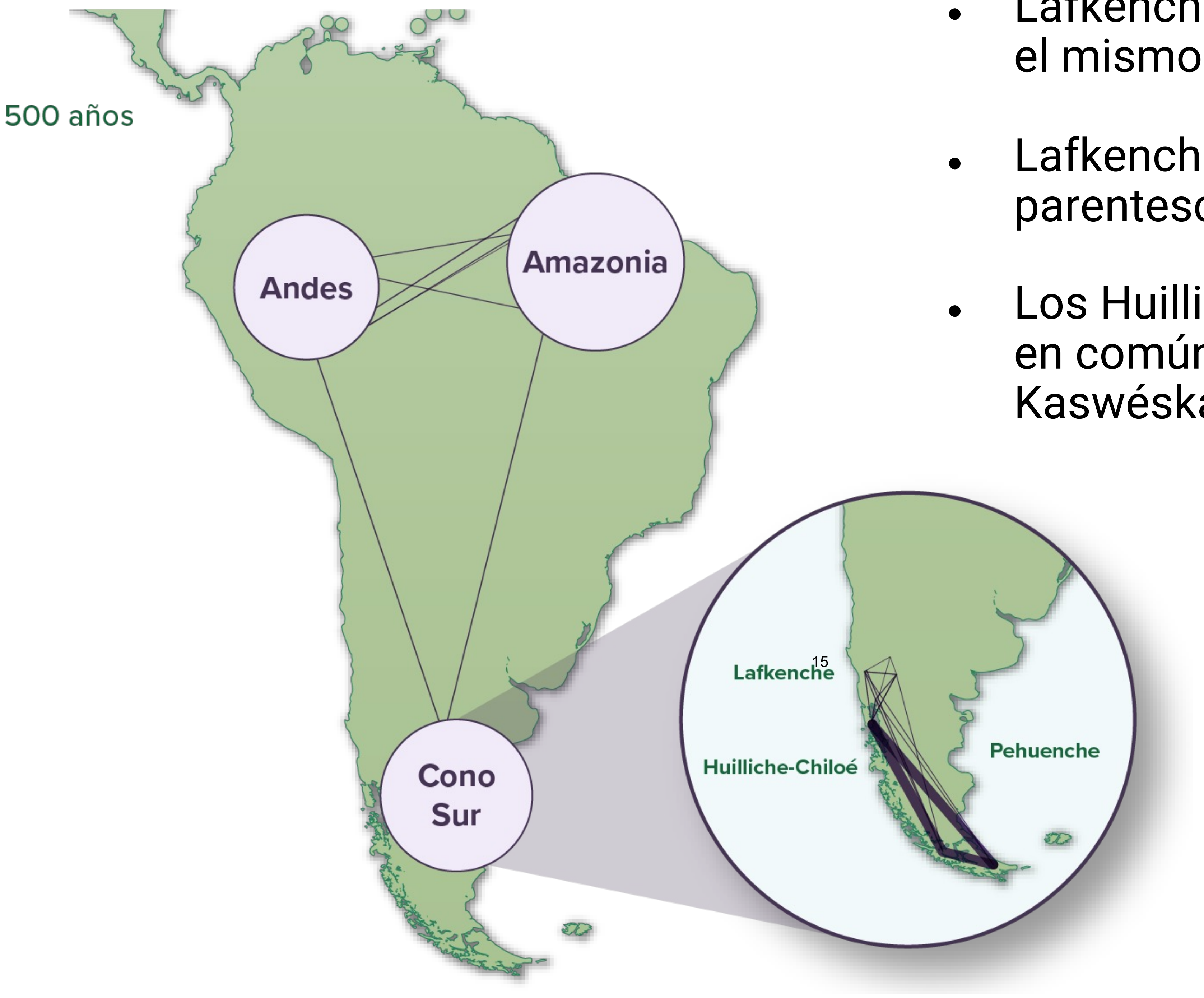

- Lafkenche, Pehuenche y Huilliche tienen el mismo linaje genético
- Lafkenche y Pehuenche tienen un fuerte parentesco.
- Los Huilliche , además, tienen ancestros en común con las poblaciones del sur, Kaswéskar y Yagán

Época colonial

Flujo genético colonial

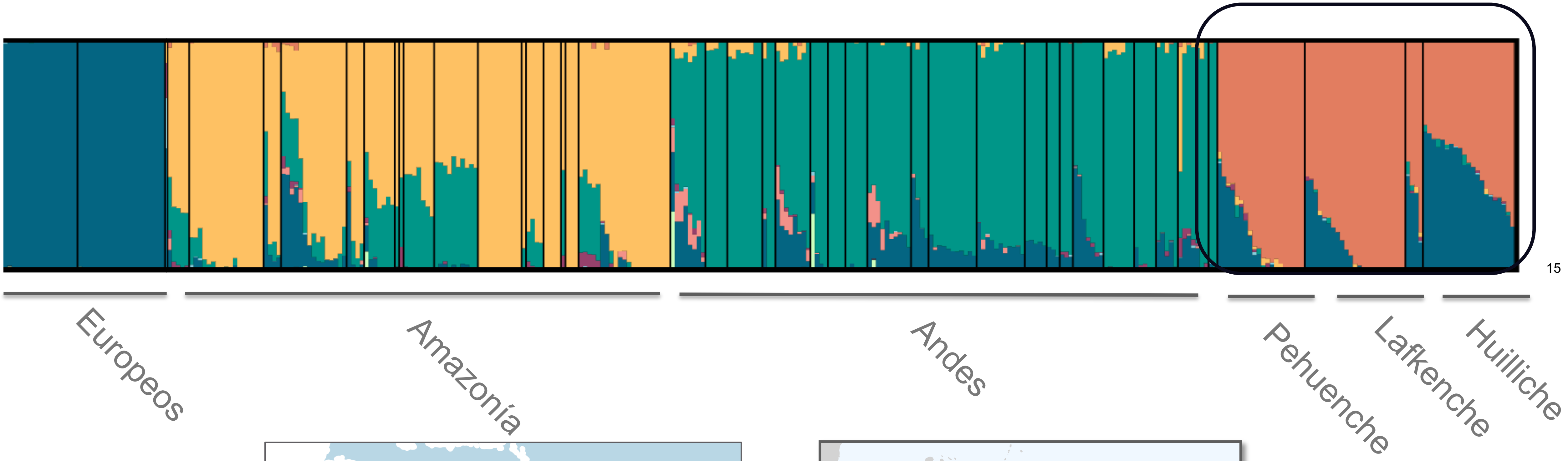

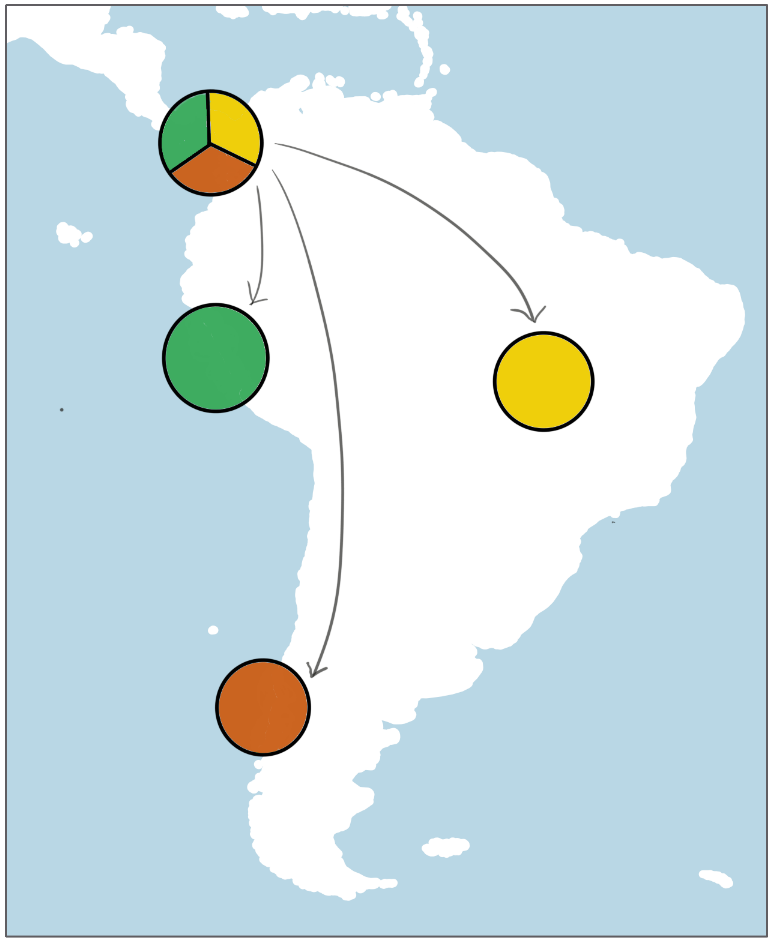

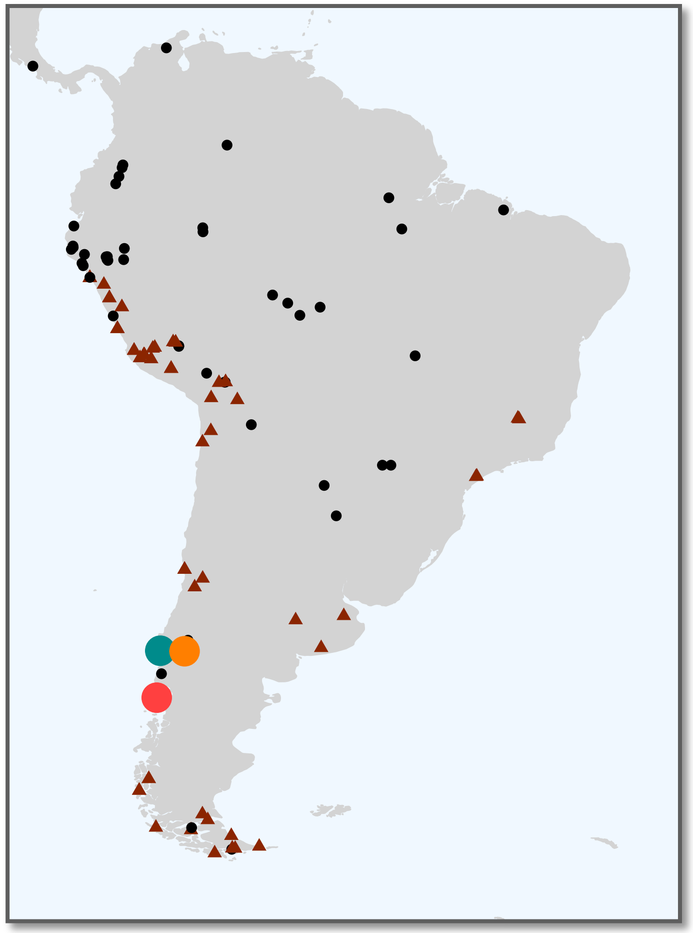

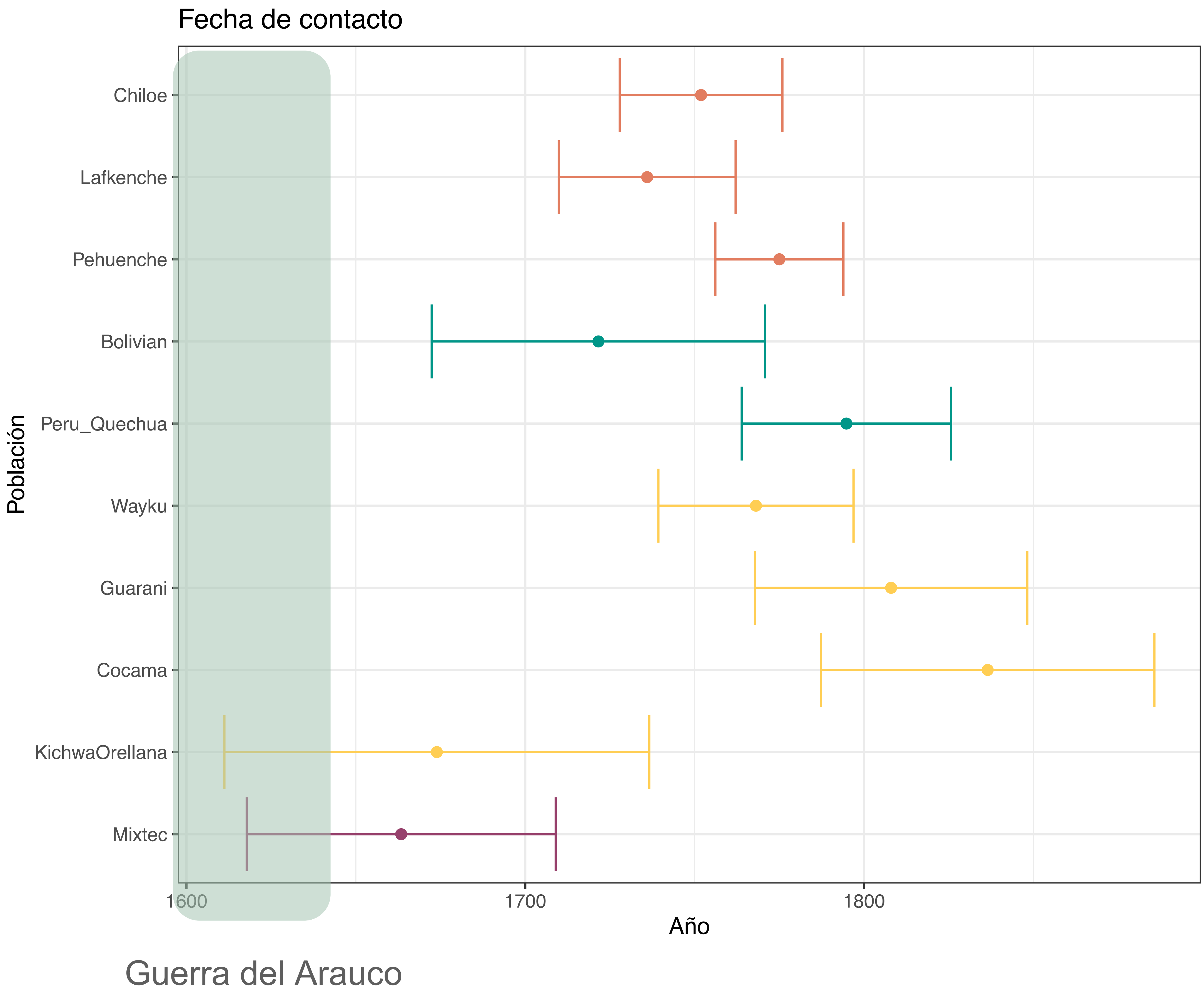
Fecha de contacto
Chiloe
Lafkenche
Pehuenche
Bolivian
Peru_Quechua
Wayku
Guarani
Cocama
KichwaOrellana
Mixtec
Población
1600
1700
1800
Año
Guerra del Arauco

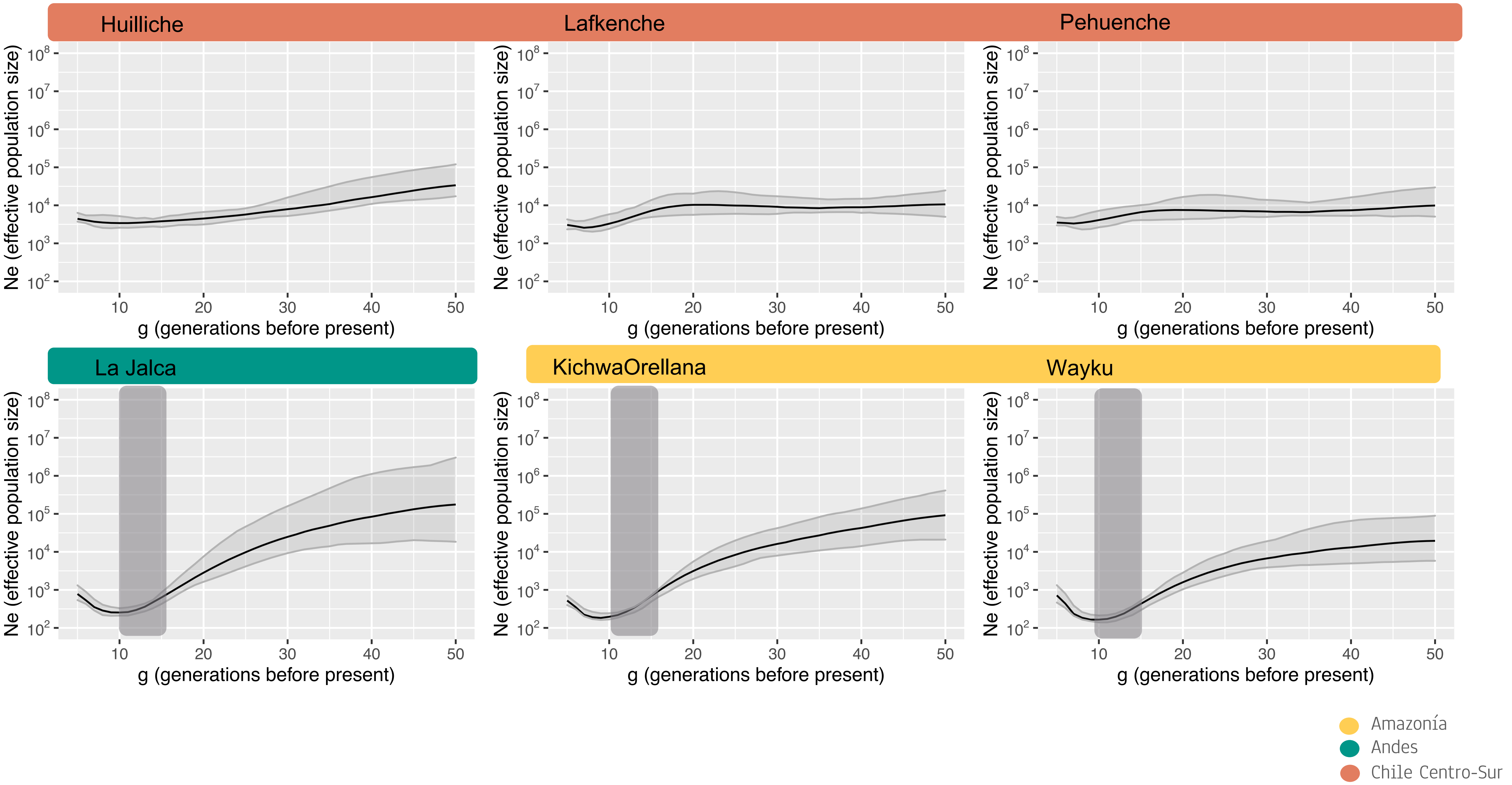
Huilliche
Lafkenche
Pehuenche
La Jalca
KichwaOrellana
Wayku
Ne (effective population size)
g (generations before present)
Amazonía
Andes
Chile Centro-Sur

Conclusiones

- Las poblaciones del Cono Sur albergan uno de los 3 principales linajes genéticos del continente.
- Dentro de el Cono Sur existen 3 grupos genéticos: Pampas Argentinas, Tierra del Fuego y Chile Centro-Sur.
- Las diferentes poblaciones Mapuche estuvieron en contacto cercano durante siglos. Huilliche comparten muchos ancestros con las poblaciones de la Patagonia.
    - Además existen algunos contactos con los Andes y la región del Gran Chaco
- El contacto con los colonizadores afecto a la demografía y la estructura genética de las poblaciones suramericanas.

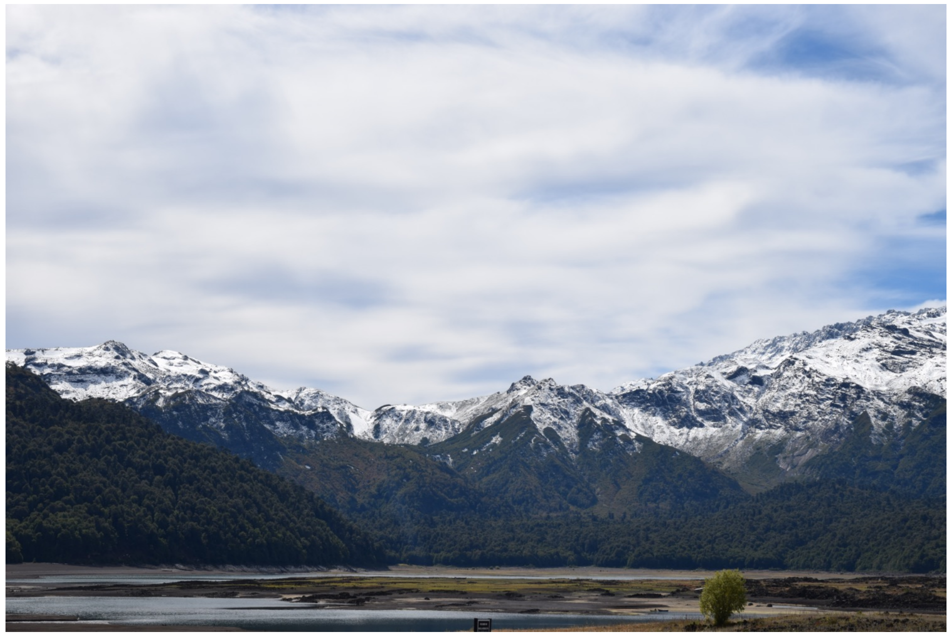

Credit: Epifanía Arango-Isaza

Etapas del proyecto (2018)

01

Diseño

Chiara Barbieri (genetista) y Paul Heggarty (lingüista e historiador) en el Max Planck de Historia Humana (Jena, Alemania)

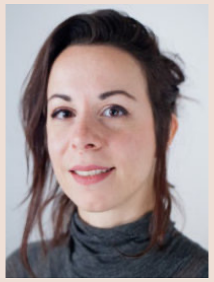

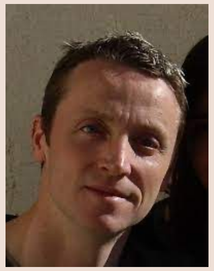

02

Colaboración con la Universidad Católica de Chile

Scott Sadowsky (lingüística) y Felipe Martínez (antropología). El proyecto se titula "**Rasgos genéticos y lingüísticos de la prehistoria poblacional de Chile**".

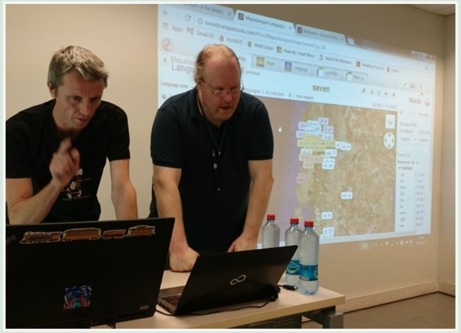

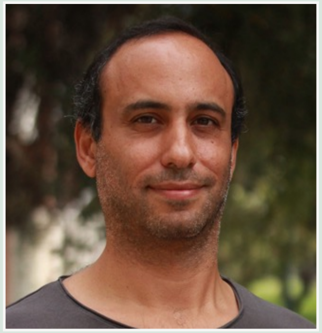

03

Aprobación del comité ético de la PUCC

Proyecto 171009001, Acta 1520863561038

Etapas del proyecto (2019)

04

Cambio de sede

Chiara empieza a trabajar en la Universidad de Zúrich, pero sigue su colaboración en Alemania

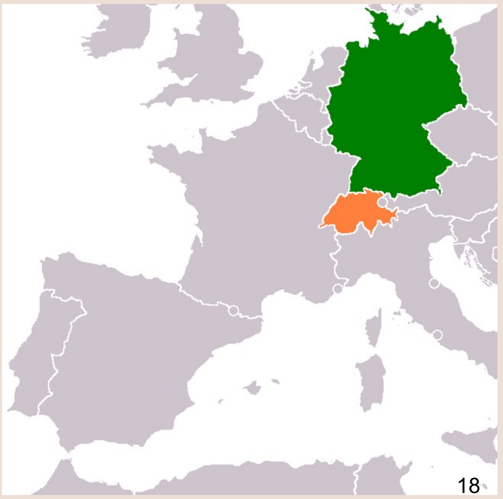

05

Financiación

Chiara consigue fondos del Instituto Max Planck para la Ciencia de la Historia Humana y la Universidad de Zúrich para viajar a Chile y recolectar las muestras.

06

Viaje a Chile

Chiara y María José Aninao (lingüísta) visitan representantes culturales, lonkos y habitantes de la Araucanía y Chiloé. Recogen muestras de saliva de 67 participantes voluntarios.

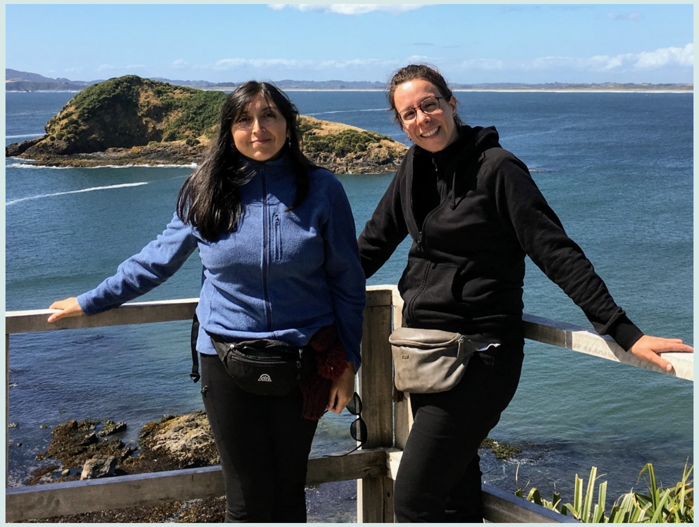

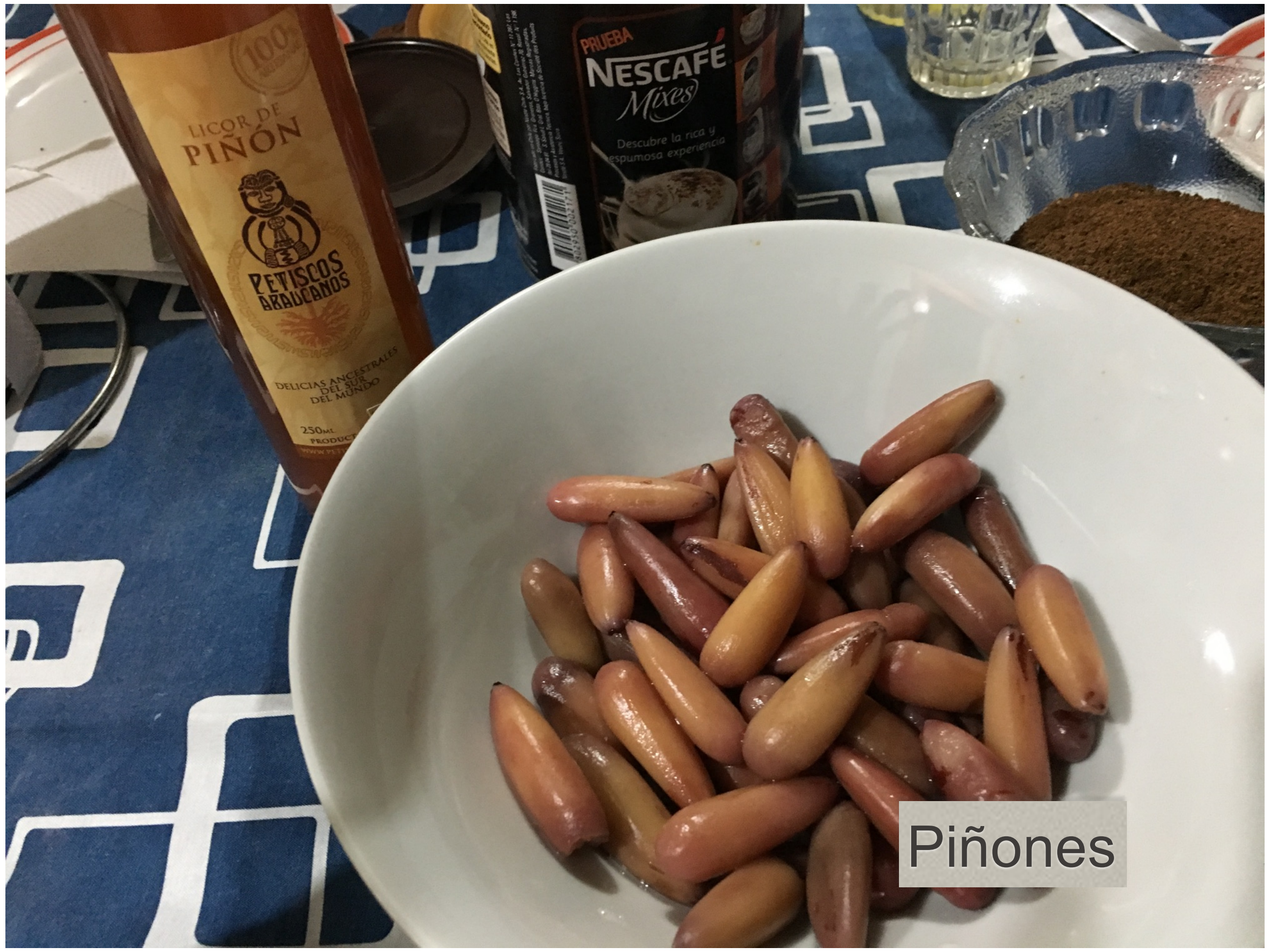

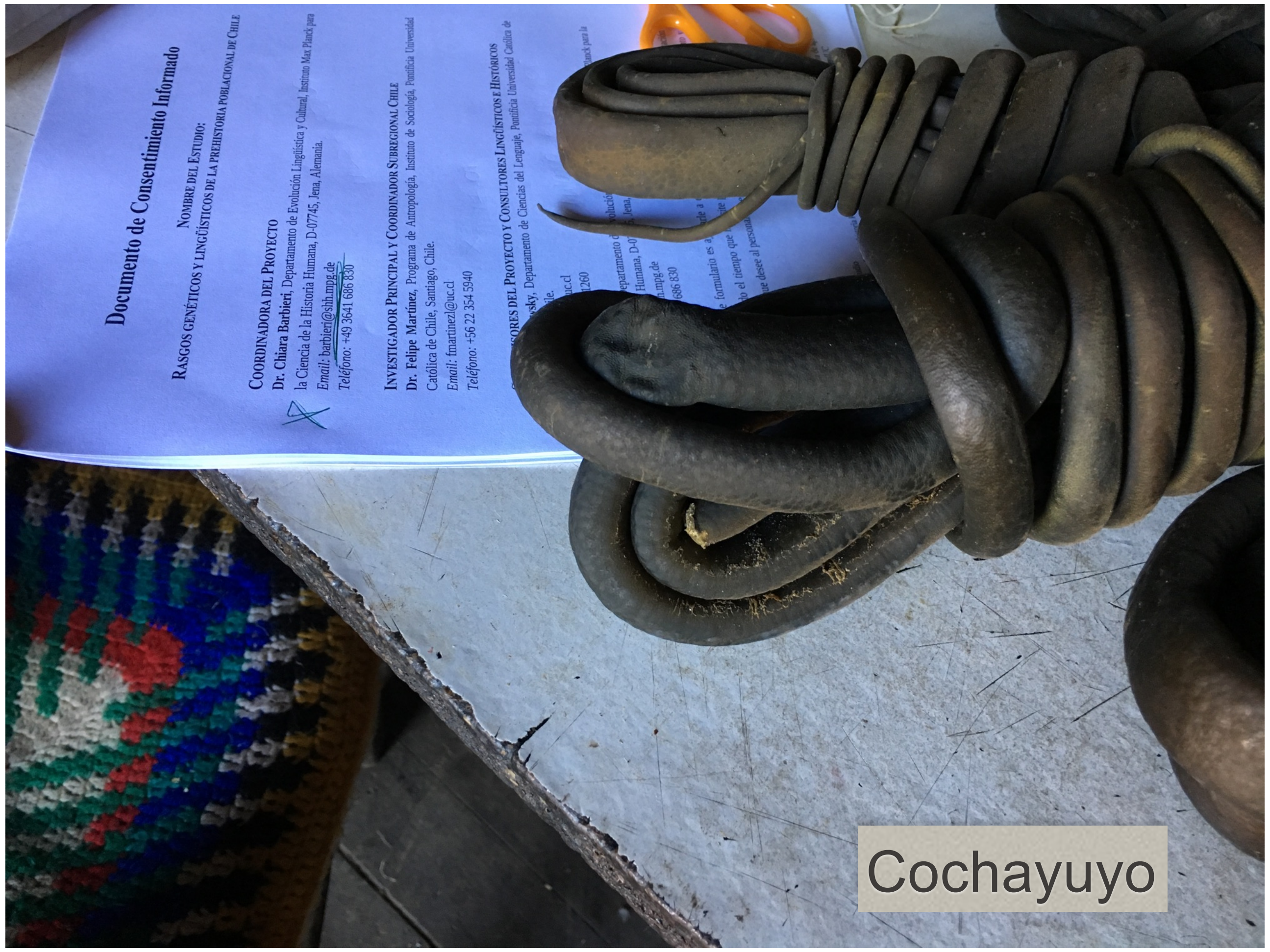

Credit:Chiara Barbieri

Etapas del proyecto (2019-2020)

07

Secuenciación

EL ADN se lleva a Alemania y Chiara hace la extracción.
En septiembre 2019 se envía a secuenciar a ATLAS BIOLAB.
El dinero viene de una beca que Chiara obtiene de la Fundación Wenner Gren para estudios antropológicos

08

Estudiante de doctorado

En septiembre Epifanía se une al laboratorio en Zúrich desde España.

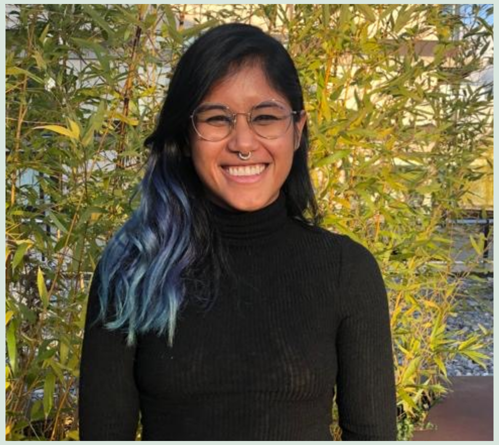

09

Los datos están listos

Empieza el análisis genético por Epifanía con supervisión de Chiara (2020-2021).
Además reciben apoyo del Profesor Kentaro Shimizu y otros genetistas como Marco Capodiferro (U.Pavía) y Cosimo Posth (U. de Tübingen)

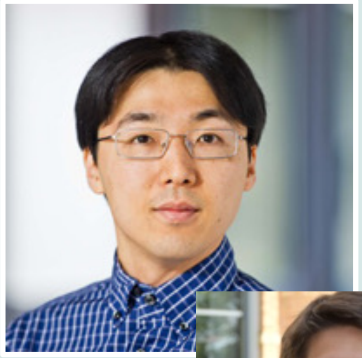

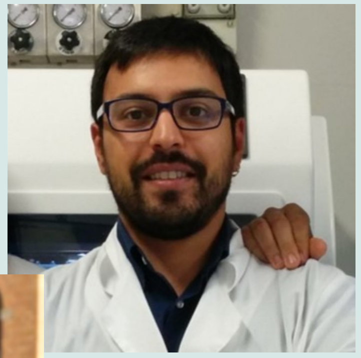

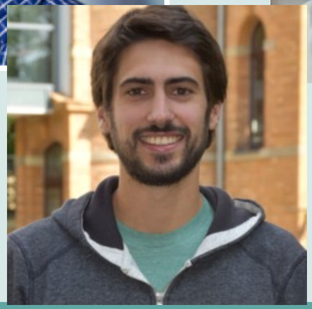

Etapas del proyecto (2021-2022)

10

Retrasos por la pandemia

Diferentes problemas logísticos

11

Búsqueda de financiación para el viaje

Epifanía -> beca de movilidad de la Universidad de Zúrich
Chiara -> recursos del Centro de Estudios Latinoamericanos de Zúrich y del proyecto NCCR "Evolving Language" de la Academia Nacional Suiza para grabar un documental de esta parte del proyecto

12

Organización del viaje

Chiara, Epifanía y María José planean el viaje

Referencias

1. Modified from Barbieri, C., Blasi, D. E., Arango-Isaza, E., Sotiropoulos, A. G., Hammarström, H., Wichmann, S., et al. (2022). A global analysis of matches and mismatches between human genetic and linguistic histories. *PNAS* 119 (47) e2122084119. doi: 10.1073/pnas.2122084119.
2. Image created by Abizar Lakdawalla. https://en.wikipedia.org/w/index.php?title=File:Radioactive_Fluorescent_Seq.jpg [Accessed August 16, 2023].
3. Artist unknown (2005). Hands at the Cuevas de las Manos upon Río Pinturas, near the town of Perito Moreno in Santa Cruz Province, Argentina. Available at: https://commons.wikimedia.org/wiki/File:SantaCruz-CuevaManos-P2210651b.jpg [Accessed November 20, 2023].
4. Archivo:Glyptodon (Riha2000).jpg - Wikipedia, la enciclopedia libre. Available at: https://commons.wikimedia.org/wiki/File:Glyptodon_(Riha2000).jpg [Accessed August 16, 2023].
5. Industry (2018). Museo conserva cráneo de mamífero gigante en Oruro | Museos de Bolivia. *Museo conserva cráneo de mamífero gigante en Oruro | Museos de Bolivia*. Available at: https://museosbolivia.blogspot.com/2018/03/museo-conserva-craneo-de-mamifero.html [Accessed August 16, 2023].
6. La noche de los gigantes (III). La Megafauna sudamericana. *La noche de los gigantes (III). La Megafauna sudamericana*. Available at: http://cronicasdefauna.blogspot.com/2017/11/la-noche-de-los-gigantes-iii-la.html [Accessed August 16, 2023].
7. AGryg (2020). English: Four Corners Potatoes starting to sprout July 2020, with red kedney bean (0.73 inch) for scale. Available at: https://commons.wikimedia.org/wiki/File:Four_Corners_Potatoes_Solanum_jamesii.jpg [Accessed November 20, 2023].
8. User:B.navez (1999).Durvillea kelp(Durvillea antartica) coastal belt of floating straps. Available at: https://commons.wikimedia.org/wiki/File:Durvillea_antarctica.JPG [Accessed November 20, 2023].
9. Pyropia (2023). *Wikipedia, la enciclopedia libre*. Available at: https://es.wikipedia.org/w/index.php?title=Pyropia&oldid=152567351 [Accessed August 16, 2023].
10. Gracilaria2.JPG (2272×1704) (n.d.). Available at: https://upload.wikimedia.org/wikipedia/commons/7/7a/Gracilaria2.JPG [Accessed August 16, 2023].
11. Ayala, F. G. P. de. 157 [159] EL OTABO CAPITÁN, APO CAMAC INGA / yndios de Chile / El octavo capitán, Camac Ynga, apu. Available at: https://commons.wikimedia.org/wiki/File:POMA0159.jpg [Accessed November 20, 2023].
12. rowanwindwhistler, E. I. I. jpg: P. america es svg: M. work: (2021). Expansión del Imperio incaico en tiempos de Huayna Cápac (1493-1525). Available at: https://commons.wikimedia.org/wiki/File:Expansion_Imperio_Inca4.svg [Accessed November 20, 2023].
13. enWiki, C. A. nur hochgeladen aus (2007). Deutsch: Farbbild eines Quipu der Inkas vom Larco Museum. Available at: https://commons.wikimedia.org/wiki/File:Inca_Quipu.jpg [Accessed November 20, 2023].
14. Rowanwindwhistler (2015). Español: Urpu para la chicha, inca. Available at: https://commons.wikimedia.org/wiki/File:UrpuParaLaChicaInca.jpg [Accessed November 20, 2023].
