# Supplementary material for: Bridging the gap: returning genetic results to indigenous communities in Latin America
Source: Front Genet. 2023 Nov 28;14:1304974. doi: 10.3389/fgene.2023.1304974 (PMC10715051; doi:10.3389/fgene.2023.1304974)
Supplement: Supplementary file 2 [file Presentation2.pdf]

Rasgos genéticos y lingüísticos de la prehistoria de Chile

Introducción y antecedentes

La genética ha experimentado un rápido desarrollo en el último siglo, desde el descubrimiento del ADN (ácido desoxirribonucleico) hasta la actualidad. El ADN es una molécula, lo que quiere decir que es una partícula muy pequeña casi invisible a la vista humana. Esta molécula almacena la información necesaria para la vida no sólo en los humanos, sino también en bacterias, árboles o incluso gatos. Puedes pensar en el ADN como un libro que contiene la información para la vida, con capítulos que abarcan desde el crecimiento, la regulación hormonal hasta el aparato digestivo... ¡y un sinfín de datos que nos permiten estar vivos!

El lenguaje que utiliza el ADN es complejo y solo tiene cuatro componentes, que identificamos con cuatro letras: A, G, T, C. Sorprendentemente, todas las instrucciones necesarias para la vida, desde la infancia hasta la edad adulta, están escritas o codificadas con estas cuatro letras. Para saber lo que está escrito en este “libro” necesitamos un proceso llamado secuenciación. Este proceso nos permite conocer el orden exacto de las letras (A, G, T y C) que componen el ADN.

A pesar de que pueda parecer increíble, el libro que contiene la información para que seas como eres no es muy diferente del libro que contiene la información para que un ratón sea un ratón. De hecho, aproximadamente el 70% de la información genética entre humanos y ratones es idéntica (Mouse Genome Sequencing Consortium et al., 2002). En el caso de los chimpancés, esta cifra puede llegar a ser de incluso el 98% (Suntsova and Buzdin, 2020). ¿Y qué ocurre entre nosotros, los seres humanos?

La diferencia entre tú y cualquier otra persona en el mundo es solo de un 0.1% (Lander et al., 2001; Venter et al., 2001; International Human Genome Sequencing Consortium, 2004). A pesar de que nuestra apariencia exterior pueda variar, rasgos como el color de los ojos o la forma de tu nariz representan solo una pequeña fracción de nuestra información genética. En resumen:

- **El 99.9% de nuestro ADN es idéntico al de cualquier otro ser humano.**
- **Solo nos diferencia un 0.1%**

¿Y qué sucede con ese 0.1% de diferencia? ¿Cómo se distribuye en las diversas poblaciones del mundo?

Los seres humanos somos una especie relativamente “joven”, con tan solo 300,000 años (Richter et al., 2017). Si comparamos esto con otros animales como algunas medusas que llevan 500 millones de años en la tierra (Dunn et al., 2008),nuestro paso en el planeta puede considerarse un breve suspiro. Durante este “corto” tiempo, las diferentes poblaciones humanas no han tenido tiempo de separarse genéticamente unas de otras, especialmente teniendo en cuenta las migraciones y otros movimientos que evitan la formación de poblaciones completamente aisladas. Como resultado, las poblaciones humanas son muy similares entre sí, y la mayor parte de la variación genética no se encuentra entre poblaciones si no entre individuos de la misma población (Lewontin, 1972; Hunley et al., 2016; Novembre, 2022).

Vamos a analizar esto paso a paso:

- De este 0.1% mencionado, la mayor parte se refiere a diferencias a nivel individual en lugar de diferencias entre las poblaciones.
- Podríamos decir que aproximadamente el 0.015% de las diferencias genéticas serían específicas de las diferentes poblaciones humanas, mientras que el 0.085% restante (y la mayor parte) se relaciona con las características individuales de cada persona (Figura 1).

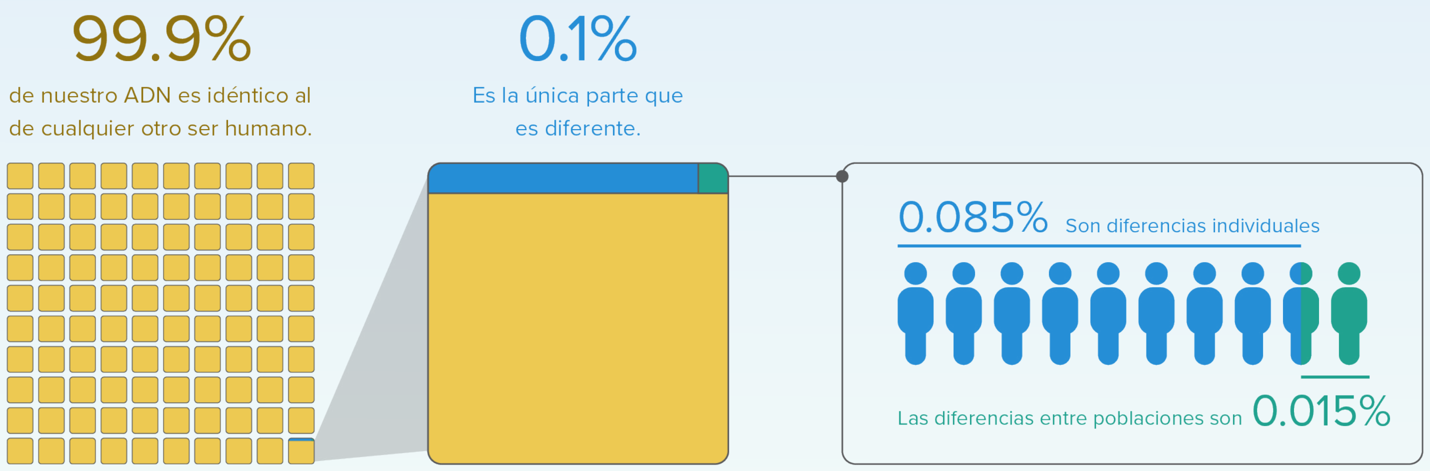

Figura 1. Ilustración de la distribución de la diversidad genética entre Individuos Humanos. El 99.9% del material genético es compartido por todos los seres humanos y solo un 0.1% de variación que contribuye a la singularidad de cada individuo. Dentro de esta variación, la mayoría está asociada con la diversidad genética a nivel individual, mientras que una fracción minoritaria (0.015%) está relacionada con las variaciones entre diferentes poblaciones.

La diferencia genética entre diferentes poblaciones es realmente mínima en comparación con el resto de nuestro ADN. A pesar de ser un porcentaje pequeño, existe un campo de estudio llamado genética de poblaciones humanas que se enfoca en ese 0.015%. Pero, ¿por qué?

Este pequeño porcentaje, aunque parezca insignificante, proporciona información valiosa sobre los movimientos migratorios de los seres humanos. La genética de poblaciones es un campo relativamente nuevo que se ha desarrollado gracias a los avances tecnológicos, pero ha revolucionado el entendimiento de la historia de los humanos como especie.

Gracias a la genética de poblaciones, conocemos con más detalle las rutas migratorias que siguieron nuestros ancestros desde África hasta el resto del planeta. Aquí tienes el camino que siguieron nuestros ancestros por todo el planeta tierra (Figura 2). ¡También otras cosas fascinantes como que muchos de los seres humanos tenemosentre 1.8-2.6 % de ADN procedente de los neandertales (Sankararaman et al., 2014; Prüfer et al., 2021)!

Para llevar a cabo todos estos estudios genéticos, es necesario secuenciar el ADN, lo que implica obtener las combinaciones de letras que están en el libro que mencionamos anteriormente. En un pasado no tan lejano, este proceso era costoso, pero gracias a los avances tecnológicos, ahora podemos obtener la secuencia del ADN de más individuos a un precio más accesible. De hecho, la ciencia ha avanzado tanto que no solo podemos obtener la secuencia de ADN de individuos contemporáneos como tú o como yo, sino también de restos humanos encontrados en yacimientos arqueológicos.

En los últimos 10 años ha habido una revolución en nuestra comprensión de la historia humana. Sin embargo, todavía existen regiones del mundo que no han sido estudiadas de manera equitativa. Por ejemplo, el caso de América, donde todavía existen muchas incertidumbres sobre conexiones entre poblaciones, y preguntas fascinantes por resolver. Con el anhelo de conocer más sobre la historia de las poblaciones de América, surge este estudio con un enfoque en el Cono Sur (que incluye Chile, Argentina y Uruguay). Este proyecto multidisciplinar sobre historia humana prehispánica surgió de una colaboración entre la Universidad Pontificia de Santiago (Chile) y la Universidad de Zúrich (Suiza), junto con otras instituciones internacionales.

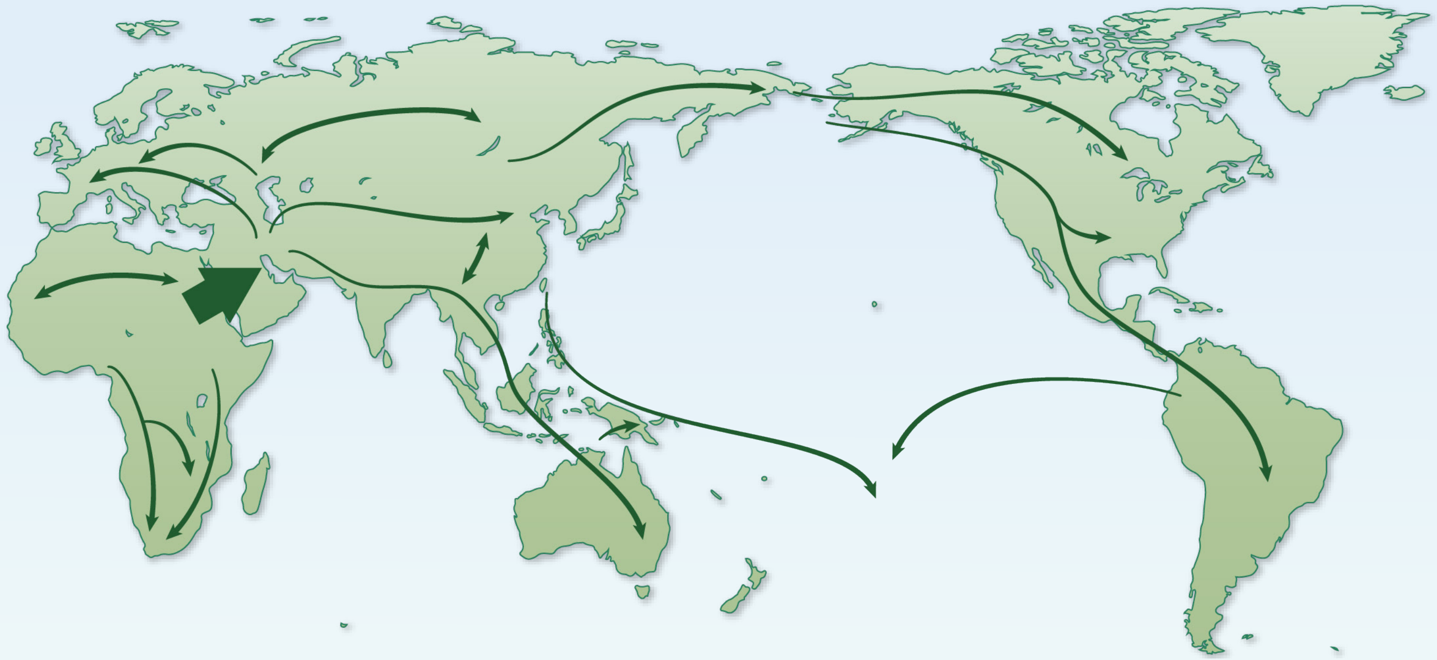

Figura 2. Reconstrucción a través de la genética de poblaciones de los movimientos humanos más significativos.

El Cono Sur es una amplia región con diferentes perfiles ecológicos (costa, montaña Andina, pampas), y presencia humana antigua y culturalmente variada. Es notable que esta región no cuenta con suficiente representación en los estudios de genética: por esto el estudio del ADN local podría ayudar en la reconstrucción prehistórica. En el Cono Sur, Chile alberga uno de los grupos indígenas más numerosos de América del Sur: los mapuches. En esta misma zona se encuentra un yacimiento arqueológico que evidencia la presencia humana más antigua de todo el continente (Dillehay et al., 2008). Es un lugar fascinante para comprender la historia de las poblaciones en América del Sur.

Los mapuches son el pueblo indígena mayoritario en Chile, y son una parte imprescindible de la historia del país. En mapudungun (la lengua de los mapuches), mapuche significa “gente de la tierra”. A la llegada de los españoles en el siglo XVI, habitaban un amplio territorio que ocupaba desde los valles de lo que hoy es Chile central hasta el archipiélago de Chiloé (Zúñiga, 2006). Actualmente los mapuches viven tanto en Chile como en Argentina. Entre los mapuches existen diferentes identidades territoriales: los lafkenches (gente del mar) que habitan la costa del Pacífico, los pehuenches (gente del árbol de la araucaria) en los Andes, los huilliches (gente del sur) desde el Toltén hasta la isla de Chiloé, y los picunches (gente del norte) que eran más numerosos en el pasado. A pesar de su importancia, la historia genética del pueblo mapuche todavía no está suficientemente clara.

A principio de 2019, la doctora Chiara Barbieri (genetista) junto con María José Aninao (lingüista) emprendieron un viaje por el sur de Chile. Durante su visita a distintas regiones habitadas por diversas comunidades mapuche y personas de ascendencia mapuche, llevaron a cabo una búsqueda de participantes para un estudio genético. Un total de 67 voluntarios donaron muestras de saliva de forma anónima. Las muestras fueron enviadas a un laboratorio en Alemania donde se extrajo el ADN para su posterior secuenciación. Una vez finalizada la secuenciación, los datos genéticos fueron analizados de forma anónima por Epifanía Arango como parte de su proyecto de doctorado, con el apoyo de Chiara Barbieri (UZH), Kentaro Shimizu (UZH), Marco Capodiferro (U.Dublin), Simon Aeschbacher (UZH) y Cosimo Posth (U. Tubingen). Además del grupo de genetistas otros colegas procedentes de otras disciplinas, como la lingüística (María José Aninao, Paul Heggarty y Scott Sadowsky), la arqueología (Roberto Campbell) y la antropología (Felipe I. Martínez), trabajaron juntos para contextualizar los resultados genéticos.

En este informe, no solo presentaremos los resultados del estudio, sino que también intentaremos contextualizar nuestros hallazgos con conocimientos procedentes de otros estudios anteriores (las referencias a estos artículos se encuentran entre paréntesis, y lamentablemente la mayoría de la información científica se publica en inglés). Antes de empezar con el reporte, debo advertir algo que a los científicos no nos gusta mencionar con frecuencia: la ciencia no posee todas las respuestas y es solo una parte del conocimiento.

El conocimiento es vasto e incluye otras formas de comprender el mundo, como la experiencia, la tradición, las leyendas y mitos, entre otros. Estos tipos de conocimiento no son mutuamente excluyentes, sino que pueden complementarse para obtener una visión más amplia de la realidad. La ciencia es simplemente una de las formas de adquirir conocimiento sobre el mundo (Haverkort, 2013; Escobar, 2016).

Otro punto importante es que afirmar que una población es más avanzada o evolucionada que otra contradice los principios de la biología y es, además, incorrecto. Cada población humana ha evolucionado o se ha desarrollado de manera única en respuesta al entorno y circunstancias históricas, lo que ha dado lugar a la diversidad cultural actual. No se puede establecer una escala lineal de progreso o de superioridad entre las poblaciones humanas, ya que todas tienen su propio conjunto de conocimientos, tradiciones y formas de vida valiosas.

Reporte del proyecto científico

Para comprender el proyecto, voy a tener que explicar primero los elementos más relevantes sobre la historia de América, ¡y resulta que esta historia se remonta a miles de años en el pasado! Para ayudarte a entender la escala temporal de la que estamos hablando, aquí te proporcionamos un diagrama ilustrativo (Figura 3). Además, es importante entender que la información que vamos a presentar en este informe se basa en las investigaciones realizadas hasta el momento. Esto significa que, si se secuencian nuevas muestras o se descubren otros yacimientos arqueológicos, toda esta información podría cambiar.

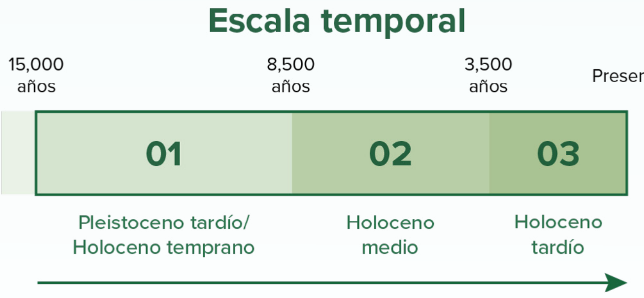

Figura 3. Escala Temporal

Según los descubrimientos actuales, los seres humanos llegaron a América a través del estrecho de Bering, que conecta Asia y América (Politis et al., 2008). Aunque el momento exacto en que cruzaron aún es desconocido, gracias a estimaciones basadas en la genética, sabemos que la población ancestral americana se separó de la población asiática hace aproximadamente 23,000 años (Raghavan et al., 2015). En aquel entonces, América era muy diferente a lo que conocemos hoy en día, ya que gran parte de América del Norte estaba cubierta por densos glaciares debido a la Edad de Hielo (Politis et al., 2008).

Conforme estos glaciares comenzaron a derretirse, las poblaciones pudieron avanzar hacia el sur, lo que llevó a la diversificación en dos poblaciones ancestrales: la población ancestral de América del Norte y la población ancestral de América del Sur (Posth et al., 2018). Como su nombre indica, la población ancestral de América del Norte ocupó principalmente este territorio y dio origen a diversas poblaciones indígenas como Cree, Algonquin o Ojibwa (Figura 4).

Por otro lado, la población ancestral de América del Sur inició una migración hacia el sur, pasando por Centroamérica y llegando hasta el extremo sur de Chile (Posth et al., 2018). En cuanto a estas poblaciones que habitaban el continente durante el Holoceno temprano, aún tenemos mucho por descubrir. Eran grupos pequeños dedicados a la caza y recolección, pero la información disponible sobre ellos es limitada (Dillehay, 1999).

No solamente hubo una migración desde América del Norte hacia Sur América, sino varias (Posth et al., 2018). Aunque aún no podemos determinar con exactitud cuándo ocurrieron, todas estas migraciones contribuyeron a la formación de un perfil genético típico de América del Sur que en el Holoceno medio dio lugar a la aparición de tres linajes genéticos principales, cada uno característico de regiones eco-geográficas diferentes: Andes, Amazonía y Cono Sur.

A medida que las condiciones climáticas mejoran y se vuelven más cálidas, se crearon ambientes propicios para sostener poblaciones más grandes, lo que a su vez impulsó el desarrollo progresivo de la domesticación tanto de plantas como animales (Politis et al., 2008). Este fue un momento crucial que definió la estructura genética de las poblaciones hasta la actualidad.

Con el transcurso del tiempo, se desarrollaron diversas culturas y civilizaciones en América del Sur, algunas de las cuales alcanzaron un alto grado de complejidad institucional, como la civilización Inca. Pero no solo

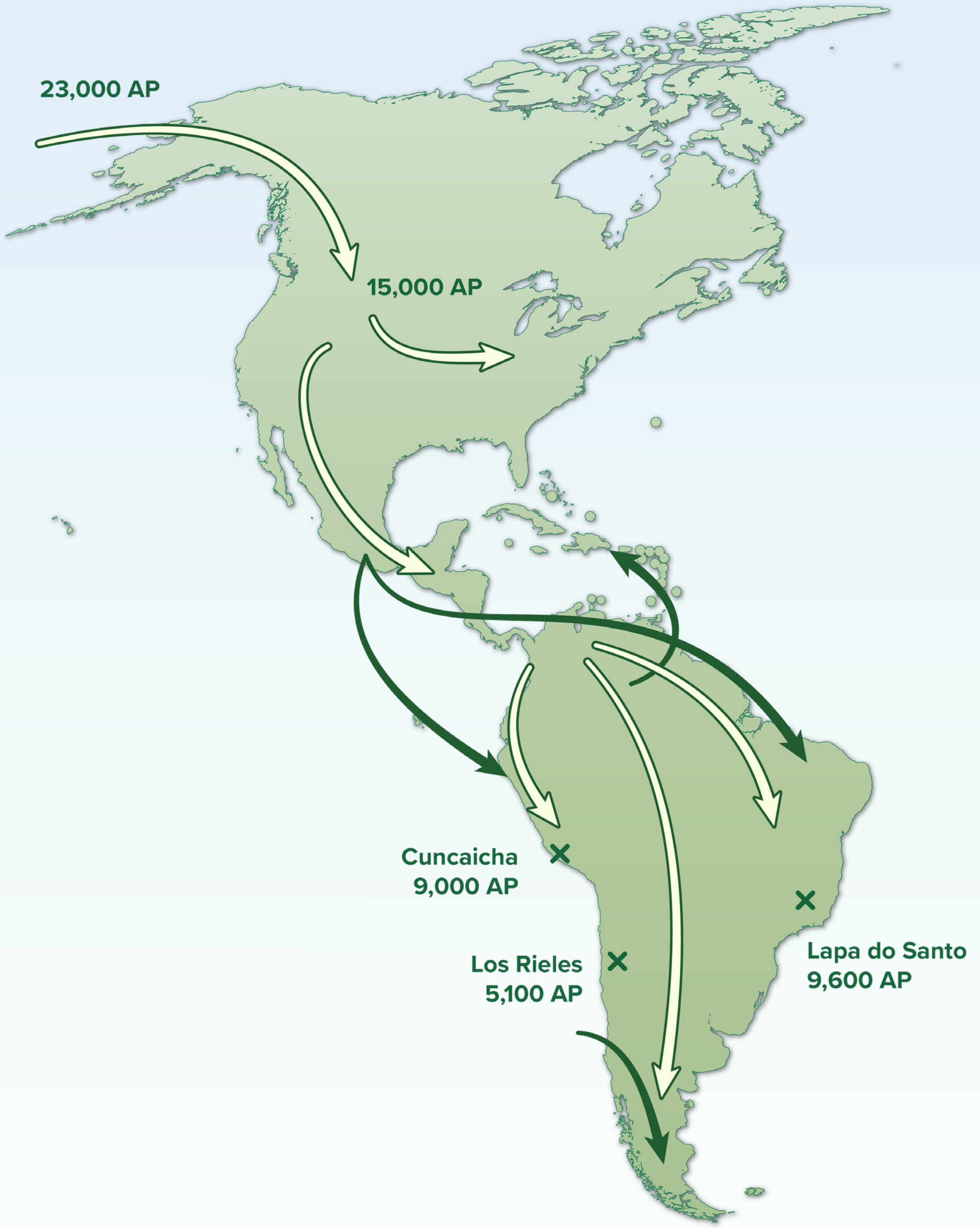

Figura 4. Reconstrucción a través de la genética de poblaciones de los movimientos humanos más significativos en América (AP: años antes del presente).

se produjo una diversidad cultural, sino también una diversidad lingüística. ¿Sabías que América del Sur se encuentra entre los lugares con mayor diversidad lingüística del mundo? Se han detectado al menos 576 lenguas indígenas de las cuales 404 siguen vivas (van Gijn et al., 2017). Esto se refiere a las numerosas lenguas indígenas que aún existen en la región y que representan un hermoso legado de la diversidad cultural del continente.

Historia del Cono Sur

En la región del Cono Sur (que incluye Chile, Argentina y Uruguay), se encuentran importantes yacimientos arqueológicos que nos permiten entender mejor la historia de esta área. El yacimiento más antiguo de todo el continente que prueba presencia humana se encuentra en esta región, con una antigüedad de 14,600 años (Dillehay et al., 2015). Además, se ha obtenido la muestra de ADN antiguo más antigua del continente, con una edad aproximada de 12,000 años (Posth et al., 2018).

Sin embargo, la información disponible sobre las poblaciones del Holoceno temprano en esta zona es limitada. Sabemos que eran grupos pequeños adaptados al uso de recursos marinos, pero se requiere más investigación para obtener más detalles sobre estas poblaciones.

Al igual que en otras zonas de América del Sur, en el Holoceno medio aparecen los linajes genéticos principales del Cono Sur. Sin embargo, estos linajes no son homogéneos, sino que se divide en tres sub-linajes que se desarrollan también durante el Holoceno medio debido a la diversificación de las poblaciones. Estos grupos habitaban las Pampas Argentinas, la Patagonia y la Zona Centro-Sur de Chile, que se caracterizan por su geografía diversa y variada (Figura 5).

Aunque contamos con restos genéticos y arqueológicos de estos grupos, no tenemos información sobre cómo se autodenominaban, su cultura o el idioma que utilizaban. Por lo tanto, los consideramos ancestros genéticos, sin especificar otras denominaciones culturales. Las poblaciones que habitaban la zona Centro-Sur de Chile son ancestros genéticos de las poblaciones mapuche actuales, mientras que las poblaciones que habitaban la Patagonia son ancestros genéticos de grupos como Yámana, Kawéskar, Selknam o Aonikenk (Nakatsuka et al., 2020).

Durante esta época, se produjeron importantes migraciones desde la zona de Chile Centro-Sur (ancestros genéticos de las poblaciones mapuche) hacia la Patagonia o zona austral. Desconocemos la naturaleza de estas migraciones, o si involucraron intercambios culturales o lingüísticos, pero seguramente dejaron un importante legado genético.

En el Holoceno tardío estas migraciones hacia el sur continuaron hasta tiempos muy recientes. Fue en este período cuando se desarrollaron las diferentes culturas que existen en la región en la actualidad. Por ejemplo, encontramos registros cerámicos de los antepasados de la cultura mapuche a partir de los primeros siglos de nuestra era (Adán et al., 2016).

En resumen, los ancestros genéticos de las poblaciones mapuche han habitado el área de Chile Centro-Sur desde hace al menos 5,000 años y han participado en varios procesos migratorios con la zona austral y posiblemente con las Pampas Argentinas. Esta ascendencia propia del centro sur de Chile se origina en continuidad con una ola migratoria temprana con un aislamiento parcial con otras zonas del continente como la Amazonia o los Andes (Figura 5).

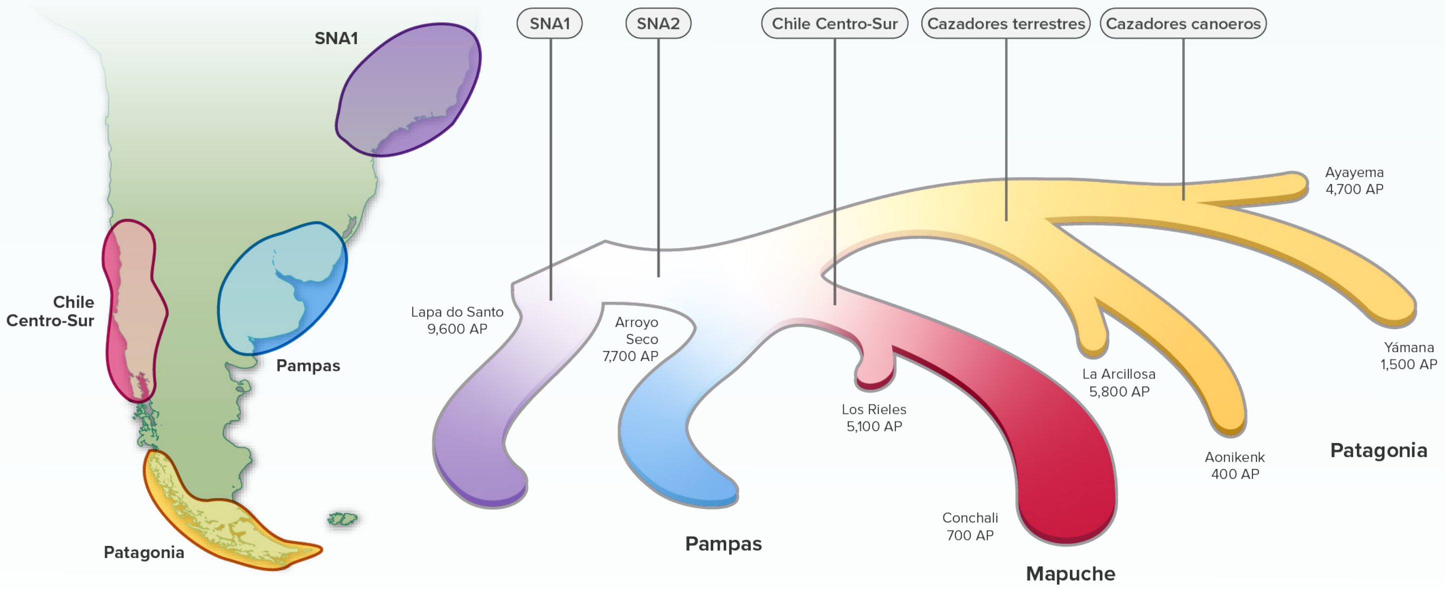

Figura 5. Representación gráfica de las conexiones entre variadas poblaciones del Cono Sur y sus posiciones geográficas. La sigla "SNA" denota la ascendencia Sur Nativo Americana (AP: años antes del presente). Figura rediseñada a partir de Arango-Isaza et al., 2023.

Historia Reciente

Los marcos temporales utilizados en genética y en arqueología son diferentes a los que estamos acostumbrados en nuestra vida diaria. En esta sección, nos gustaría brindarte más información sobre la historia reciente de las poblaciones del Cono Sur.
Como ya mencionamos anteriormente, existen tres linajes genéticos principales en Sur América, presentes en los Andes, la Amazonia y el Cono Sur. Los Andes y la Amazonía están más interconectados entre sí, y se han detectado importantes contactos genéticos entre ellas, mientras que el Cono Sur está más aislado, aunque no completamente. Hemos identificado la presencia de conexiones con la región del Gran Chaco y con los Andes centrales (Figura 6.A).

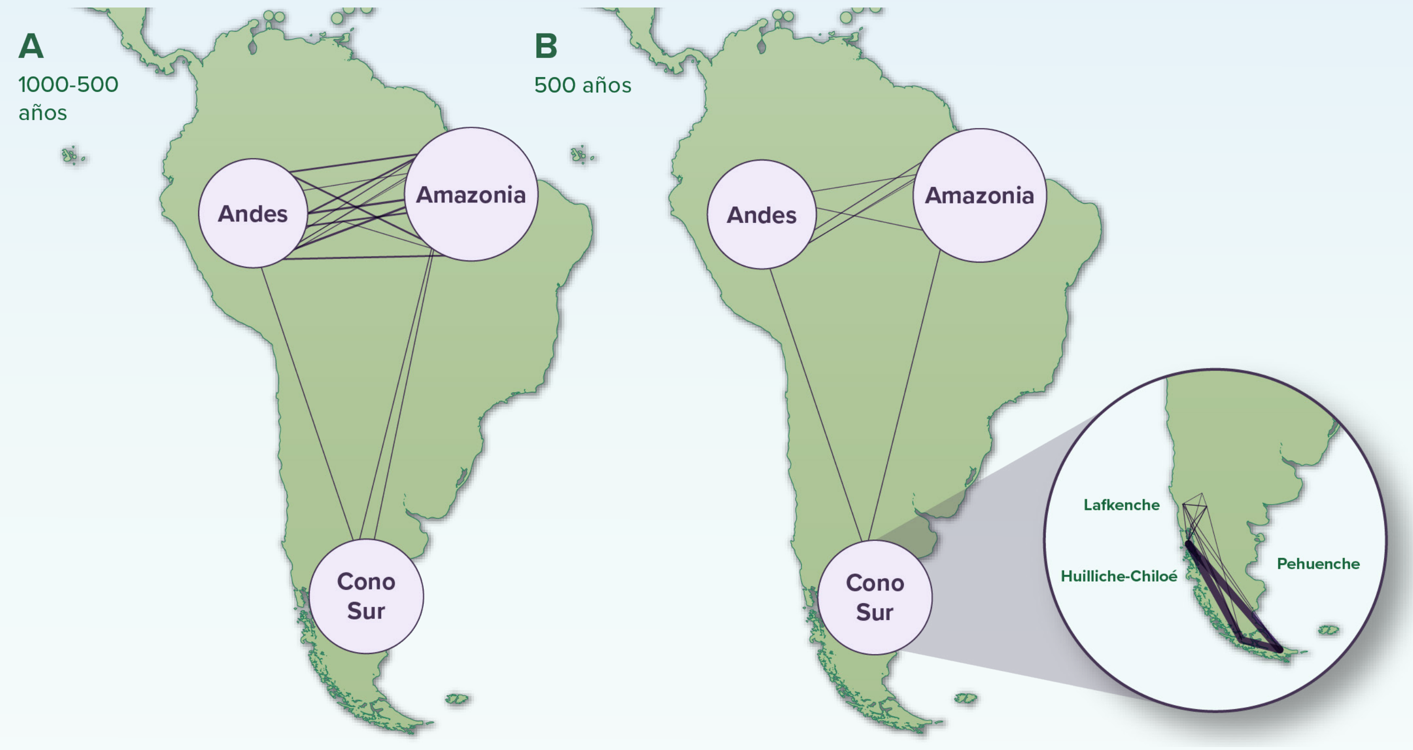

Figura 6. Red de interacción genética entre poblaciones sudamericanas. El primer mapa presenta interacciones que tuvieron lugar entre hace A) 1000 y 500 años, mientras que B) el segundo mapa muestra eventos más recientes, ocurridos en un período inferior a 500 años. Figura rediseñada a partir de Arango-Isaza et al., 2023.

La conexión con los Andes precede incluso al imperio incaico en 1450, y no solo implica un legado genético, sino también el intercambio de cultivos, rasgos culturales y palabras. Por ejemplo, la palabra "pez", que en quechua es "chalwa", fue prestada al mapudungun. Ahora, ambas lenguas utilizan la misma palabra debido a este préstamo. Además de esas conexiones con otras regiones, encontramos patrones interesantes dentro de la distribución de linajes genéticos en el Cono Sur.

Además, observamos que las diferentes identidades territoriales mapuche están emparentadas entre sí. Aunque los huilliches de la isla de Chiloé tienen una conexión persistente entre las poblaciones mapuche y las poblaciones más australes (Figura 6.B). A través de conversaciones con colegas de diferentes campos y los propios habitantes de la isla, hemos concluido que esta conexión se puede explicar con la presencia de un grupo llamado Chono, que pertenece al grupo genético de las poblaciones patagónicas. Aparentemente este grupo habitaba el golfo de Corcovado y el sur de la isla de Chiloé donde probablemente ocurrió el intercambio genético. Otra evidencia de la presencia chono, aparte de la arqueología, son los topónimos (nombres de lugares) de origen Chono que encontramos en la isla, que no provienen del mapudungun, tal como Achao, que es el nombre de una isla cerca de Chiloé.

Por último, hablaremos sobre el impacto que tuvo la llegada de los colonizadores españoles a Chile a partir de 1520. Esta llegada provocó grandes cambios demográficos y culturales en las poblaciones mapuche, quienes se organizaron para proteger sus tierras e identidad. Durante todo el periodo del imperio español, se produjo un cambio drástico en su forma de vida.
La introducción de ganado y el impacto de la guerra provocó un cambio en su subsistencia. Además, se produjo una integración con otras poblaciones, como

los tehuelches o aonikenk de las pampas argentinas, que antes estaban más aisladas, en un intento de resistir tanto a nivel poblacional como cultural. Este conflicto bélico entre españoles y población indígena se conoce como la Guerra del Arauco, que duró más de cien años, aproximadamente desde 1550 hasta 1650, aunque los historiadores no están completamente de acuerdo en las fechas exactas.

A pesar de las grandes pérdidas de vidas y las dificultades en la lucha, las poblaciones mapuches lograron proteger parte de su territorio, lo que condujo a la independencia del imperio español, un logro sin precedentes en Sudamérica. En nuestro estudio, observamos que las tres poblaciones Mapuche involucradas en este estudio mostraron evidencia de flujo genético colonial. Este contacto genético fue más notable alrededor del año 1750, después de la guerra, lo que sugiere interacciones genéticas significativas con los europeos. Además, es importante destacar que el flujo genético entre los colonos y las poblaciones mapuche no debe interpretarse como una medida de superioridad o inferioridad. Más bien, refleja las complejas interacciones y mezclas culturales que se produjeron durante ese período histórico. Estas interacciones genéticas son un testimonio de la resiliencia y adaptabilidad del pueblo mapuche, quienes han conservado su identidad cultural a pesar de la influencia colonial.

Es fundamental reconocer y valorar la rica diversidad cultural de los mapuches, así como su papel en la historia de Chile y Sudamérica. El estudio de la historia mapuche no solo nos brinda una comprensión más profunda de su pasado, sino que también contribuye a promover el respeto y la igualdad entre todas las poblaciones, rompiendo con narrativas discriminatorias o de supremacía.

Asimismo, es recomendable explorar diferentes fuentes y perspectivas para obtener una visión más completa y contextualizada de la historia mapuche. Los aportes de historiadores como José Bengoa, así como de investigadores y académicos mapuches, son esenciales para comprender la complejidad de esta historia y el legado de resistencia y lucha por la autodeterminación de este pueblo (Comunidad de Historia Mapuche Ta iñ Fijke Zipa Rakizuameluwün, 2012; Nahuelpan, 2013; Quemenado et al., 2019; Sadowsky y Aninao, 2019). Nuestro proyecto se ha centrado principalmente en la investigación de la prehistoria, de la cual apenas había registros disponibles desde la genética. Esperamos que hayas disfrutado de este viaje de aprendizaje sobre los pueblos originarios de América del Sur y su fascinante historia. Antes de concluir este informe, queremos hacer una reflexión final: la ascendencia genética no es equivalente a la identidad. La identidad se construye a través de elementos variables como la cultura, el sentimiento de pertenencia y otros factores, y no está determinada por la biología o al menos no únicamente. Los linajes genéticos y culturales señalan aspectos diferentes de las personas, aunque en algunos casos pueden estar relacionados.

Este proyecto representa solo una pequeña contribución, ya que aún queda mucho más por investigar y descubrir. De hecho, este proyecto ha planteado más preguntas que esperamos que la comunidad científica pueda ir respondiendo gradualmente. Asimismo, deseamos que, en el futuro, la ciencia se enfoque cada vez más en valorar la diversidad y reconocer la importancia de los pueblos originarios.

Es fundamental seguir promoviendo el respeto, la inclusión y el diálogo intercultural en el estudio de los pueblos originarios, reconociendo su sabiduría ancestral y su contribución invaluable a la historia y la identidad de nuestras sociedades. A través de una colaboración respetuosa y basada en la reciprocidad, podremos avanzar hacia una comprensión más profunda y holística de nuestro pasado y construir un futuro más equitativo y enriquecedor para todas las culturas.

Realizado por Epifanía Arango Isaza,
Universidad de Zúrich, Suiza.
Contacto: epifaniarango@gmail.com

Science Graphic Design

Diseño gráfico por Science Graphic Design

Bibliografía

- Arango-Isaza, E., Capodiferro, M. R., Aninao, M. J., Babiker, H., Aeschbacher, S., Achilli, A., et al. (2023). The genetic history of the Southern Andes from present-day Mapuche ancestry. Current Biology. doi: 10.1016/j.cub.2023.05.013.
- Comunidad de Historia Mapuche Ta iñ Fijke Zipa Rakizuameluwün (2012). Ta iñ fijke xipa rakizuameluwün. Historia, colonialismo y resistencia desde el país Mapuche. Temuco Chile: Ediciones Comunidad de Historia Mapuche.
- Dillehay, T. D. (1999). The late Pleistocene cultures of South America. Evol. Anthropol. Issues News Rev. 7, 206–216. doi: 10.1002/(SICI)1520-6505(1999)7:6<206::AID-EVAN5>3.0.CO;2-G.
- Dillehay, T. D., Ocampo, C., Saavedra, J., Sawakuchi, A. O., Vega, R. M., Pino, M., et al. (2015). New Archaeological Evidence for an Early Human Presence at Monte Verde, Chile. PLOS ONE 10, e0141923. doi: 10.1371/journal.pone.0141923.
- Dunn, C. W., Hejnol, A., Matus, D. Q., Pang, K., Browne, W. E., Smith, S. A., et al. (2008). Broad phylogenomic sampling improves resolution of the animal tree of life. Nature 452, 745–749. doi: 10.1038/nature06614.
- Escobar, A. (2016). Sentipensar con la Tierra: Las Luchas Territoriales y la Dimensión Ontológica de las Epistemologías del Sur. AIBR Rev. Antropol. Iberoam. 11, 11–32. doi: 10.11156/aibr.110102.
- Haverkort, B. ed. (2013). Hacia el diálogo intercientífico: construyendo desde la pluralidad de visiones de mundo, valores y métodos en diferentes comunidades de conocimiento. Primera edición. La Paz, Bolivia: Plural Editores : Facultad de Ciencias Agrícolas, Pecuarias, Forestales y Veterinarias, UMSS.
- Hunley, K. L., Cabana, G. S., and Long, J. C. (2016). The apportionment of human diversity revisited. Am. J. Phys. Anthropol. 160, 561–569. doi: 10.1002/ajpa.22899.
- International Human Genome Sequencing Consortium (2004). Finishing the euchromatic sequence of the human genome. Nature 431, 931–945. doi: 10.1038/nature03001.
- Lander, E. S., Linton, L. M., Birren, B., Nusbaum, C., Zody, M. C., Baldwin, J., et al. (2001). Initial sequencing and analysis of the human genome. Nature 409, 860–921. doi: 10.1038/35057062.
- Lewontin, R. C. (1972). “The Apportionment of Human Diversity,” in Evolutionary Biology: Volume 6, eds. T. Dobzhansky, M. K. Hecht, and W. C. Steere (New York, NY: Springer US), 381–398. doi: 10.1007/978-1-4684-9063-3_14.
- Mouse Genome Sequencing Consortium, Waterston, R. H., Lindblad-Toh, K., Birney, E., Rogers, J., Abril, J. F., et al. (2002). Initial sequencing and comparative analysis of the mouse genome. Nature 420, 520–562. doi: 10.1038/nature01262.
- Nahuelpan, H. (2013). Las “zonas grises” de las historias mapuche. Colonialismo internalizado, marginalidad y políticas de la memoria. Rev Hist Soc.
- Nakatsuka, N., Luisi, P., Motti, J. M. B., Salemme, M., Santiago, F., D’Angelo del Campo, M. D., et al. (2020). Ancient genomes in South Patagonia reveal population movements associated with technological shifts and geography. Nat. Commun. 11, 3868. doi: 10.1038/s41467-020-17656-w.
- Novembre, J. (2022). The background and legacy of Lewontin’s apportionment of human genetic diversity. Philos. Trans. R. Soc. B Biol. Sci. 377, 20200406. doi: 10.1098/rstb.2020.0406.
- Politis, G. G., Prates, L., and Pérez, S. I. (2008). El poblamiento de América: arqueología y bioantropología de los primeros americanos. EUDEBA, Buenos Aires. Ciudad de Buenos Aires: Editorial Universitaria de Buenos Aires.
- Posth, C., Nakatsuka, N., Lazaridis, I., Skoglund, P., Mallick, S., Lamnidis, T. C., et al. (2018). Reconstructing the Deep Population History of Central and South America. Cell 175, 1185-1197.e22. doi: 10.1016/j.cell.2018.10.027.
- Prüfer, K., Posth, C., Yu, H., Stoessel, A., Spyrou, M. A., Deviese, T., et al. (2021). A genome sequence from a modern human skull over 45,000 years old from Zlatý kůň in Czechia. Nat. Ecol. Evol. 5, 820–825. doi: 10.1038/s41559-021-01443-x.
- Quemenado, P. M., Nahuelquir, F., Paillal, J. M., Montalva, M. C., and Levil, R. (2019). ¡Allkütunge, wingka! ¡Ka kiñechi!: ensayos sobre historias mapuche. Ediciones Comunidad de Historia Mapuche.
- Raghavan, M., Steinrücken, M., Harris, K., Schiffels, S., Rasmussen, S., DeGiorgio, M., et al. (2015). Genomic evidence for the Pleistocene and recent population history of Native Americans. Science 349. doi: 10.1126/science.aab3884.
- Richter, D., Grün, R., Joannes-Boyau, R., Steele, T. E., Amani, F., Rué, M., et al. (2017). The age of the hominin fossils from Jebel Irhoud, Morocco, and the origins of the Middle Stone Age. Nature 546, 293–296. doi: 10.1038/nature22335.
- Sadowsky, S., and Aninao, M. J. (2019). “Internal migration and ethnicity in Santiago,” in The Routledge Handbook of Spanish in the Global City (Routledge).
- Sankararaman, S., Mallick, S., Dannemann, M., Prüfer, K., Kelso, J., Pääbo, S., et al. (2014). The genomic landscape of Neanderthal ancestry in present-day humans. Nature 507, 354–357. doi: 10.1038/nature12961.
- Suntsova, M. V., and Buzdin, A. A. (2020). Differences between human and chimpanzee genomes and their implications in gene expression, protein functions and biochemical properties of the two species. BMC Genomics 21, 535. doi: 10.1186/s12864-020-06962-8.
- van Gijn, R., Hammarström, H., van de Kerke, S., Krasnoukhova, O., and Muysken, P. (2017). “Linguistic Areas, Linguistic Convergence and River Systems in South America,” in The Cambridge Handbook of Areal Linguistics Cambridge Handbooks in Language and Linguistics., ed. R. Hickey (Cambridge: Cambridge University Press), 964–996. doi: 10.1017/9781107279872.034.
- Venter, J. C., Adams, M. D., Myers, E. W., Li, P. W., Mural, R. J., Sutton, G. G., et al. (2001). The Sequence of the Human Genome. Science 291, 1304–1351. doi: 10.1126/science.1058040.
- Zúñiga, F. (2006). Mapudungun: el habla mapuche: introducción a la lengua mapuche, con notas comparativas y un CD. Santiago de Chile: Centro de Estudios Públicos.
